# Effectiveness of the novel 3D PreemieScanner for preterm infants’ growth monitoring confirmed in a simulated setting

**DOI:** 10.3389/fmedt.2025.1607538

**Published:** 2025-08-01

**Authors:** Ronald H. J. van Gils, Onno K. Helder, René F. Kornelisse, Timothy M. S. Singowikromo, Irwin Reiss, Jenny Dankelman

**Affiliations:** ^1^ Department of Neonatal and Pediatric Intensive Care, Division of Neonatology, Erasmus MC University Medical Center, Rotterdam, Netherlands; ^2^ Department of Create4Care, Erasmus MC University Medical Center, Rotterdam, Netherlands; ^3^ Department of Biomechanical Engineering, Faculty of Mechanical Engineering, Delft University of Technology, Delft, Netherlands; ^4^ Research Centre Innovations in Care, Rotterdam University of Applied Sciences, Rotterdam, Netherlands; ^5^ Institute of Engineering & Applied Science, Rotterdam University of Applied Sciences, Rotterdam, Netherlands

**Keywords:** growth monitoring, neonatal intensive care unit (NICU), preterm infants, extremely low birth weight infants (ELBW infants), 3D scanning and measuring, head circumference (HC), cranial volume (CrV), neonatal stress reduction

## Abstract

**Introduction:**

Preterm infants’ growth is typically monitored through weight, body length (BL) and head circumference (HC). However, 3D cranial volume (CrV) is considered a more accurate indicator of brain growth than 2D HC. The PreemieScanner is a novel 3D measuring device that simultaneously measures BL, HC and CrV. Its clinical usability was tested in a simulated NICU setting.

**Materials and methods:**

Three extremely low birth weight (ELBW; BW < 1,000 gram) dolls with Optiflow breathing systems, (tubes positioned either at the front or back of the head) were used. Nurses conducted scan sessions and marked anatomical landmarks on 3D PreemieScanner images. As control, nurses measured HC manually with a standard measuring tape. Key outcomes were: (1) Measurement success rate, (2) Precision—percentage within clinically allowed limits, ±0.4 cm for BL, ±0.3 cm for HC, ±12 ml for CrV, and 3) accuracy—mean or median measurement error (MME) relative to the ground truth.

**Results:**

Thirty-five scan sessions resulted in 100% successful measurements for BL and HC; 80% for CrV. BL MME −3.3% (*p* < 0.001); 40% (42/105) within precision limits. HC MME (Optiflow-front) 0.0% (*p* = 0.63); 89% (51/57) within limits. HC MME (Optiflow-back) −0.4% (*p* = 0.91). 93% (43/46) within limits. MME HC measuring tape, (Optiflow-front) −0.8% (*p* < 0.001), 88% (50/57) within limits, and MME (Optiflow-back) −1.1% (*p* < 0.001), 83% (40/48) within limits. MME CrV (Optiflow-front) −1.8% (*p* = 0.01), 86% (31/36) within limits, MME CrV (Optiflow-back) −1.3% (*p* < 0.001), 98% (45/46) within limits.

**Conclusions:**

The PreemieScanner is a reliable, comprehensive device for measuring BL, HC and CrV in ELBW infants. It integrates smoothly into routine care with minimal disturbance. HC measurements demonstrated higher accuracy and precision than traditional tape method. CrV measurements, with 93% within precision limits, can be regarded as acceptable, enabling development of CrV growth reference charts, enhancing clinical growth monitoring.

## 1 Introduction

Growth monitoring is a worldwide acknowledged cornerstone of neonatal intensive care. For optimal short- and long-term health outcomes, preterm infants' growth velocity should align with standardized growth reference charts. Impaired growth velocity has been associated with conditions such as necrotizing enterocolitis, bronchopulmonary dysplasia, and malabsorption (1–5).

In neonatal intensive care units (NICU), the growth of preterm infants is typically monitored through daily weight checks, and weekly measurements of body length (BL) and head circumference (HC) (6). However, measuring HC and BL measurements inside the incubator with instruments using tools like calipers or measuring tapes can cause considerable stress to the infant (7–9). Such stress is associated with suboptimal brain development (10, 11) and other negative health effects (12–16). Acknowledging the harmful impact of neonatal stress, NICUs have increasingly adopted individualized care programs focused on stress prevention, which have become a standard of care (17–19).

Three-dimensional cranial volume (CrV) measurements are considered a more accurate and clinically reliable indicator of brain growth in preterm infants compared to 2D HC measurements (20–24). However, unlike the widely accepted growth reference charts for preterm HC, there are no established CrV growth reference data for preterm infants (25–27). This gap is largely due to the technical and practical challenges of obtaining stress-free measurements in ventilated infants within incubators. Vermeulen et al. presented the first CrV growth reference charts for preterm infants born at 34–42 weeks gestational age (GA) and emphasized the need for measuring techniques applicable to younger infants (28). Additionally, Dieks et al. presented longitudinal HC and CrV data for infants born between 28 and 40 weeks GA (29).

**Figure 1:**
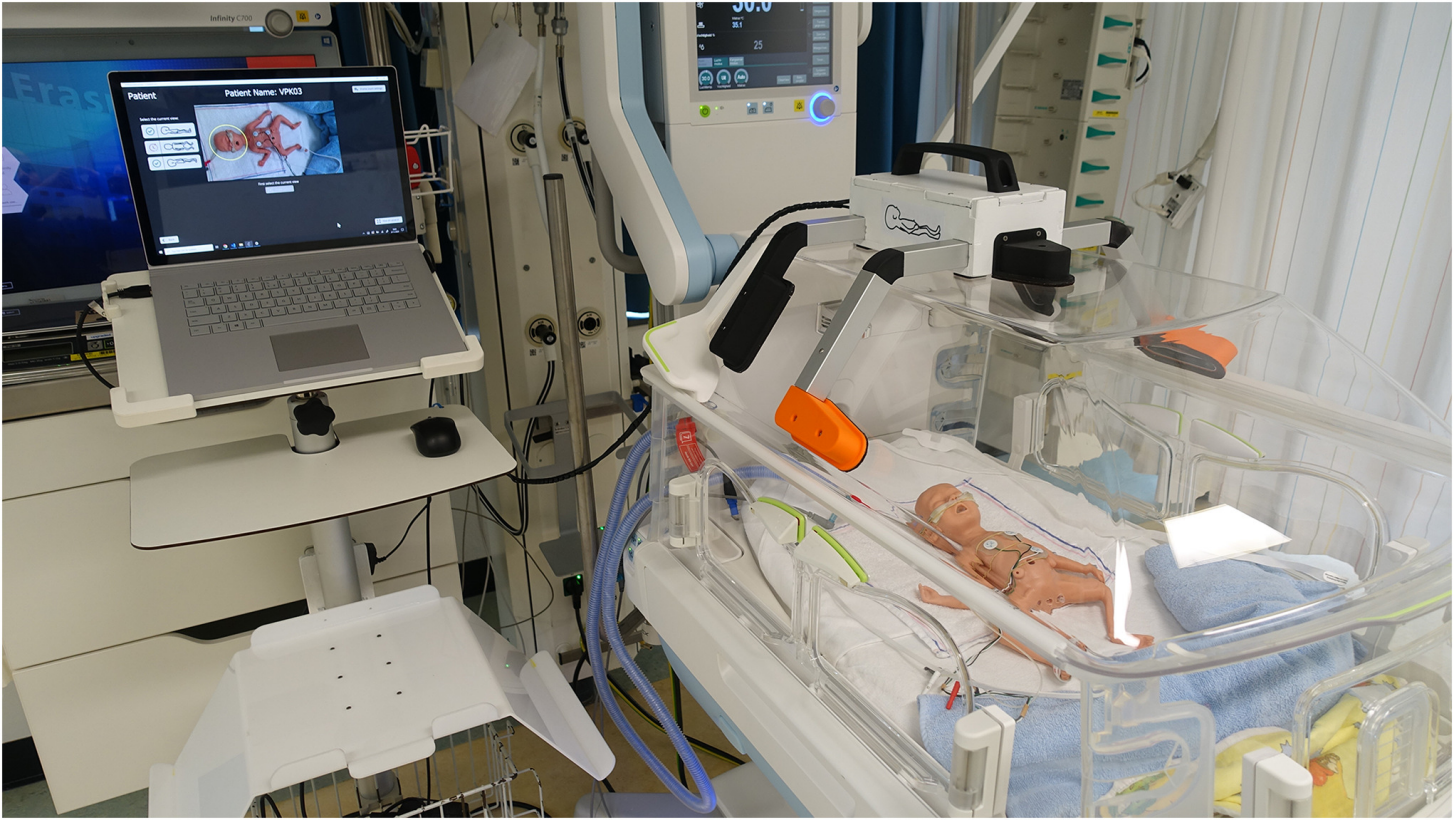
The PreemieScanner, a portable device containing five 3D cameras, is placed on the incubator's transparent cover and operated via a connected laptop. Activating the “Create Capture” button on the laptop touchscreen captures a 3D image, constructed from the five overlapping 3D raw camera images. Initial capture time of the individual cameras is approximately 30 ms, during which the infant should lie completely still. Total capture time, including construction of the assembled 3D image, is approximately 5 s Demo video available on YouTube: PreemieScanner 2024.

A reliable, stress-free technique for CrV measurements in preterm infants within incubators is needed for routine care. Ideally, an all-in-one growth monitoring device should measure BL, HC and CrV without causing stress, even for extremely low birth weight (ELBW; BW < 1,000 gram) infants. To address this need, the PreemieScanner, a 3D scanner, has been developed (Figure 1). Previously, the MONITOR3D scanner was developed and validated for measuring HC and CrV of ELBW infants within incubators (30). However, MONITOR3D had limitations: it could not measure BL, and only 45% of scans resulted in successful measurements—insufficient for clinical implementation. Additionally, its accuracy and precision needed improvement to be applicable to a broader preterm population. The PreemieScanner aims to overcome these limitations. This study evaluate its clinical usability in a simulated neonatal intensive care unit (NICU) setting by assessing: (1) the percentage of successful measurements from PreemieScanner scan sessions, and (2) the percentage of measurements within clinically allowed precision limits.

## 2 Materials and methods

In June 2,024, a validation study in a simulated setting was conducted at the NICU of the Erasmus MC—Sophia Children's Hospital, which admits approximately 600 neonates annually. It comprises four open baby sub-units, each with eight beds. At the time, the clinical staff included 17 neonatologists, 9 residents, 91 nurses, 16 nurse trainees, 22 nursing assistants, and 9 nurse practitioners.

### 2.1 Research ethic committee approval

The Clinical Technological Research—Human Research Ethics Committee (KTO-HREC) of Delft University of Technology approved the study (Application number KTO-HREC23002). All collected data were stored anonymously.

### 2.2 The PreemieScanner

The PreemieScanner used in this study is an in-hospital developed medical device currently in the prototyping stage and not commercially available. This portable scanner comprises five eye-safe 3D cameras (Intel RealSense, Santa Clara, California, USA) mounted in fixed positions within a frame. This frame is designed to fit precisely over the transparent cover of a Babyleo TN500 incubator (Dräger, Lübeck, Germany), enabling positioning of the 3D cameras around the infant's head and body (Figure 1).

For a BL measurement, a single 3D capture is sufficient, provided it offers an unobstructed view on the crown, crotch, knee, and heel. This capture should ideally be taken during diaper change, ensuring the infant remains still for less than a second. Immediately after the capture, nurses can resume their routine care, while the actual BL measurement can be calculated afterward.

For HC and CrV measurements, three captures are needed, each taken with the head in a different position (front, left, right). These captures generate overlapping 3D images that collectively cover all areas of the head. The three, overlapping 3D images are “merged” by 3D postprocessing to one 3D image that represents the complete, 360 degrees around, image of the head.

To minimize stress caused by head repositioning, captures are synchronized with the protocolized head position changes that are part of NICU standard care. These position changes, performed every three to four hours, help prevent skull deformations (31, 32). The approach of synchronizing capture moments with routine care is detailed in a previous study (30).

### 2.3 Simulated NICU care setting

**Figure 2:**
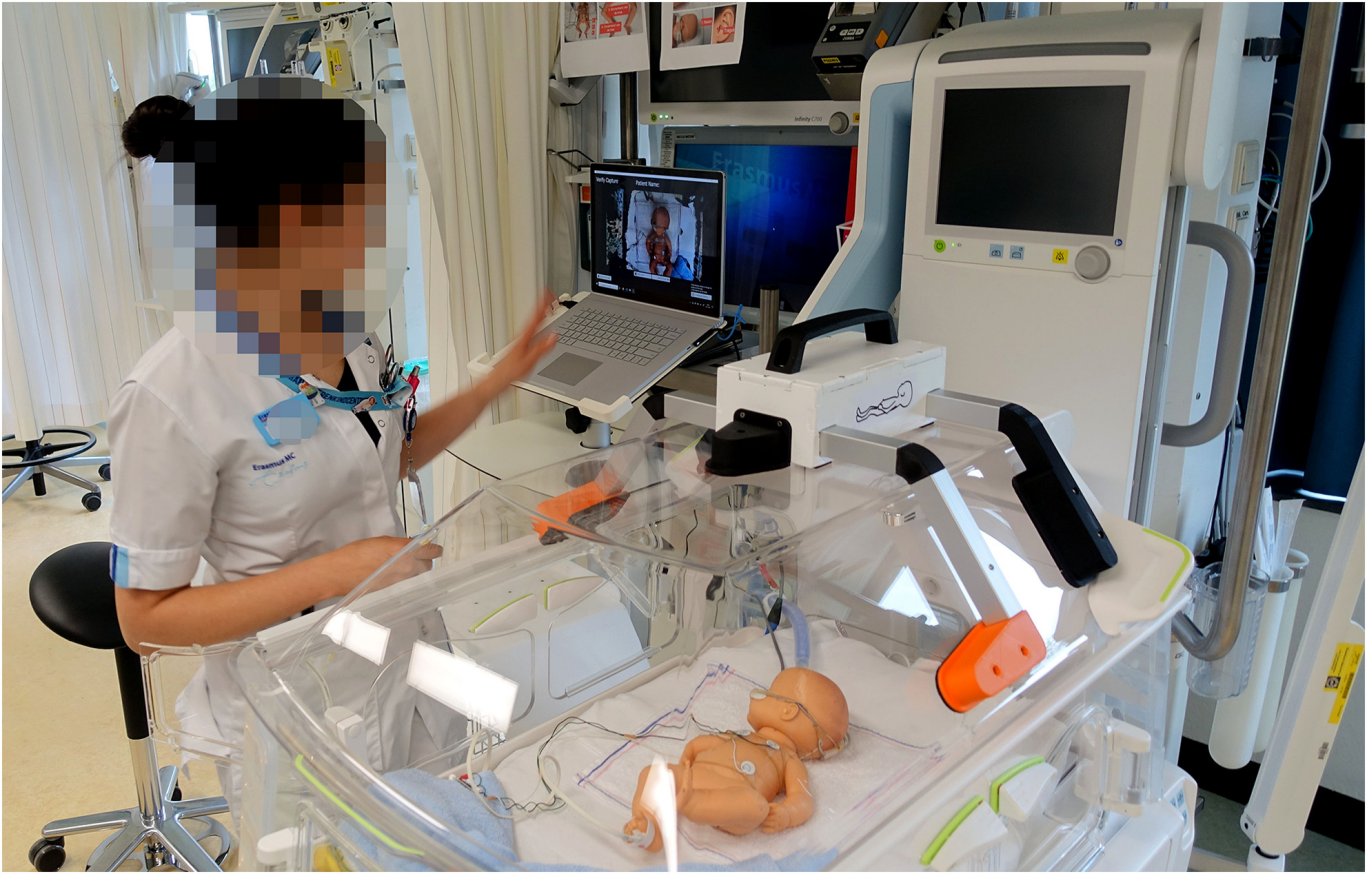
Scan session in a NICU bed space. The nurse operated the scanner using a laptop, controlled either via a wireless computer mouse or the built-in touchscreen.

The validation took place in an NICU incubator bedspace, where prepared dolls were placed in an incubator to simulate ELBW infants (birth weight < 1,000 gram). After having been shown an instructional video, NICU nurses individually conducted scan sessions using the PreemieScanner scanner, with a preterm doll placed in an incubator (Figure 2) to mimic a NICU routine care moment. Two researchers were present to guide the nurse throughout the scan session.

**Figure 3:**
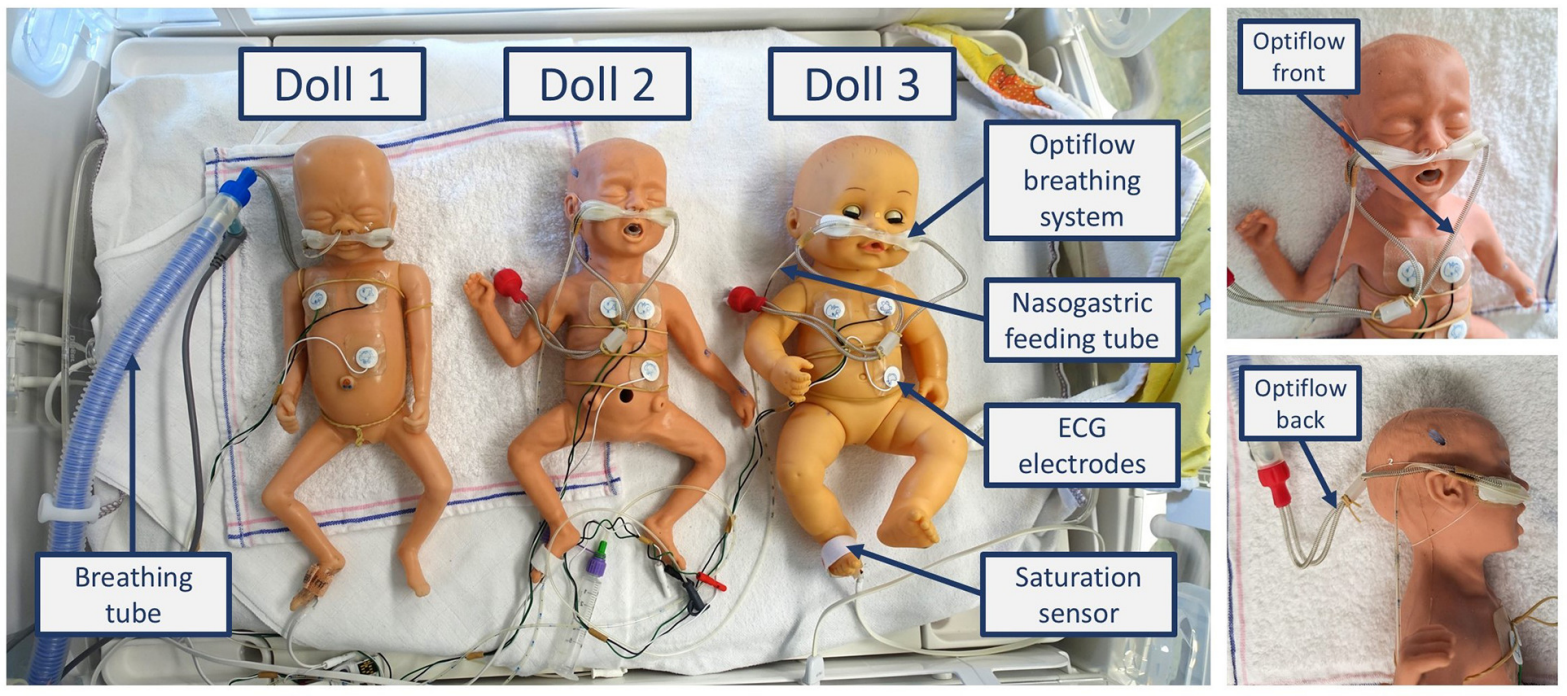
The three prepared dolls. Each doll was equipped with an Optiflow breathing system (Optiflow Junior 2 Nasal Interface and WigglewiNG, Fisher & Paykel Healthcare, Auckland, New Zealand), ECG electrodes (ConMed 1741-003 ECG Neotrode), a nasogastric feeding tube (Vygon UK Ltd, Swindon, UK 1311.05), and a saturation sensor (Masimo Corporation, Irvine, CA, USA).

Three dolls were used (Figure 3), each representing a slightly different ELBW infant, weighing approximately 600–800 grams and measuring 32–35 cm in length. Two of the dolls were preterm simulation dolls (doll 1: SOMSO Premature Baby type MS61, Female, SOMSO MODELLE GmbH, Coburg, Germany; doll 2: Nasco Premature baby, type LF01206U), while the third was a baby doll (doll 3: Citi Toy, type BD034B). Each doll was equipped with a respiratory support system (Optiflow, Fisher & Paykel Healthcare, Auckland, New Zealand) and a feeding tube, both secured to the nose and forehead with commonly used tape. To replicate a real NICU care situation, the doll was positioned in a supportive 'snuggle' with a rolled-up towel around the legs. The Optiflow tubes were prepared in two configurations to align with typical NICU practices, depending on the infant's position: tubes placed at the front of the face (Optiflow-front) or at the back of the head (Optiflow-back). For the HC and CrV measurements, the two tube positions (Optiflow-front and Optiflow-back) were analyzed separately to assess the effect of tube placement on 3D head measurements.

## 3 Data collection

**Figure 4:**
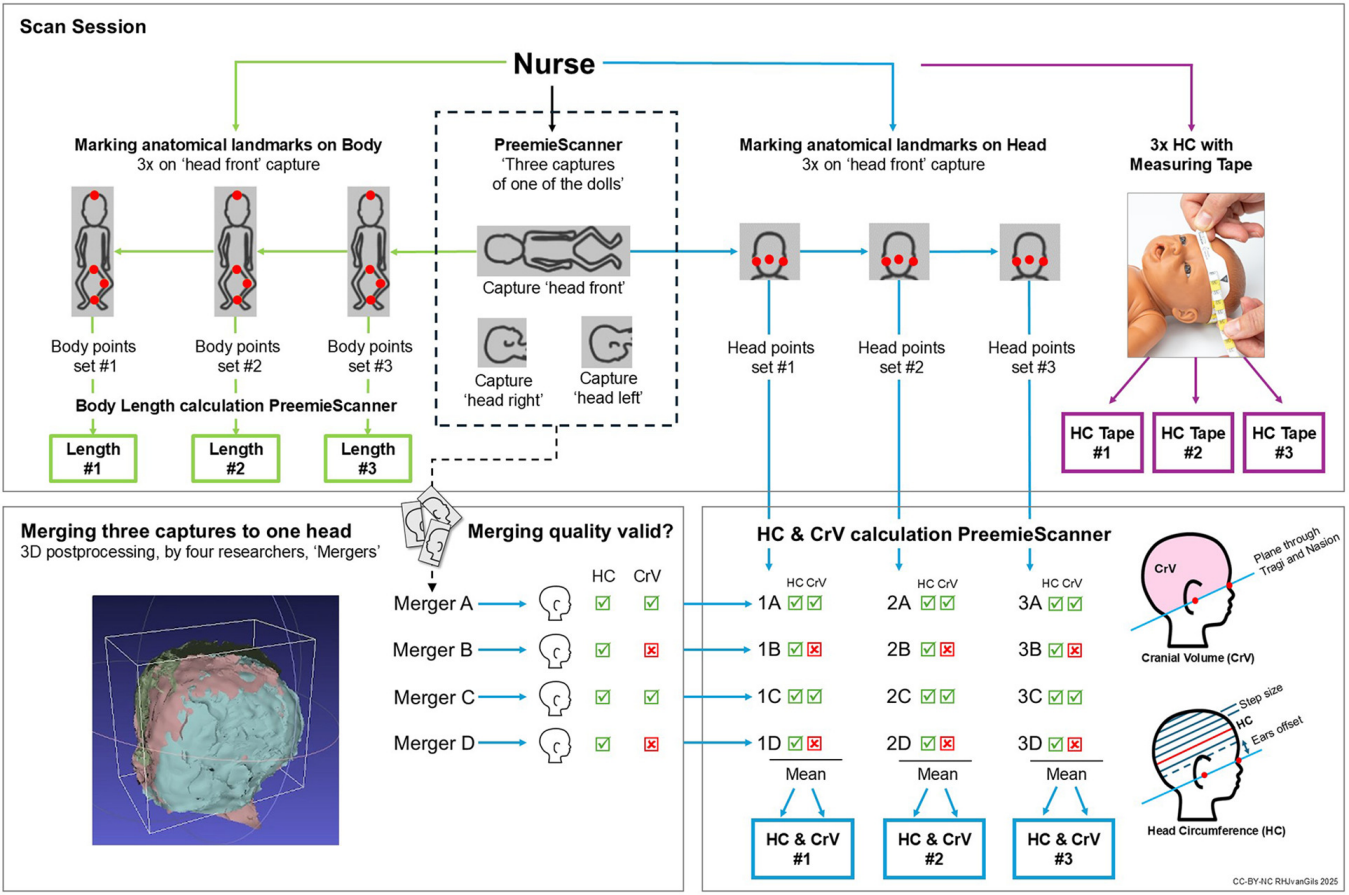
Data collection flowchart. In a scan session, each nurse individually conducted three 3D captures of a doll. After the first capture was taken—with the doll's head facing forward, the nurse marked anatomical landmarks on the 3D image on the laptop screen: on the body for BL, and on the head for HC/CrV. This marking process was repeated three times—first for BL, then for HC/CrV –resulting in three consecutive PreemieScanner measurements of BL, HC and CrV. After marking the landmarks on the first capture, two more captures were taken, with the head facing to the right and to the left, repositioning the doll in between captures. No landmarks were marked on the second and third capture. At the end of the scan session, the nurse measured the doll's HC three consecutive times using a standard measuring tape.

Figure 4 illustrates the data collection process for BL, HC and CrV measurements through a flowchart. of the process involved four steps, described in detail in the following paragraphs: (1) Scan sessions, (2) Merging three captures into one head, (3) Visual assessment of merging quality, (4) Calculating BL, HC, and CrV. All raw 3D data and additional information are accessible in the online dataset (33). The readme file of the dataset is available as Supplementary Material.

### 3.1 Scan sessions

Each nurse conducted three 3D captures of one of the three dolls, marked anatomical landmarks on the 3D image on the laptop screen, and measured the doll's HC using a standard measuring tape. Doll type and Optiflow method were randomly assigned across scan sessions and nurses, aiming for balanced sample sizes.

**Figure 5:**
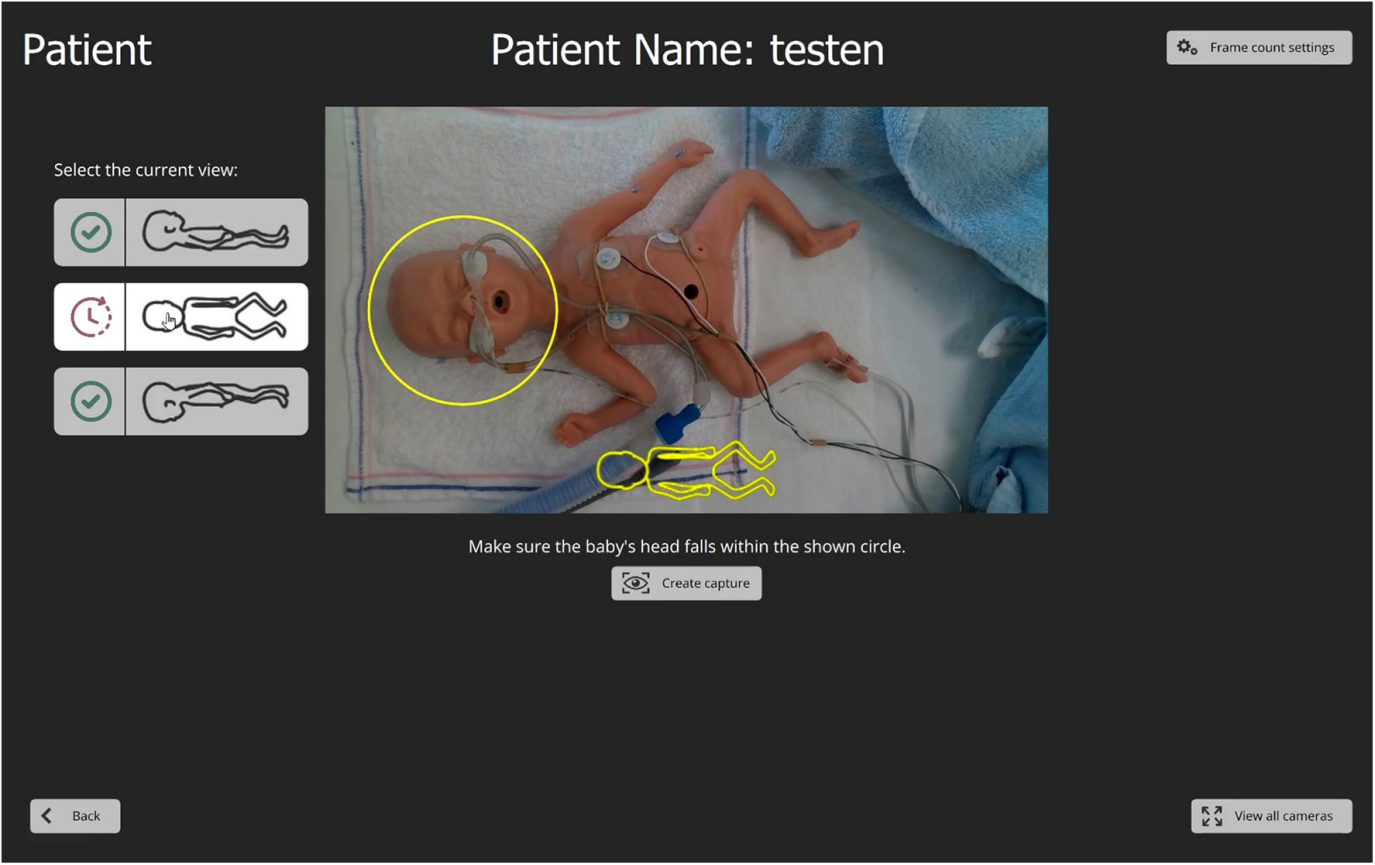
The PreemieScanner graphical user interface. The interface is displayed on a touchscreen laptop. A “live” camera preview helps the nurse align the scanner with the head, ensuring proper positioning within a designated circle by gently sliding the scanner over the incubator cover. Before capturing, the nurse selects the head's orientation using graphical head contours buttons that match the camera preview. Once the “Create capture” button is pressed, a 3D file of the capture is automatically saved to the laptop, labelled with a suffix indicating the head's position (front, left, right).

**Figure 6:**
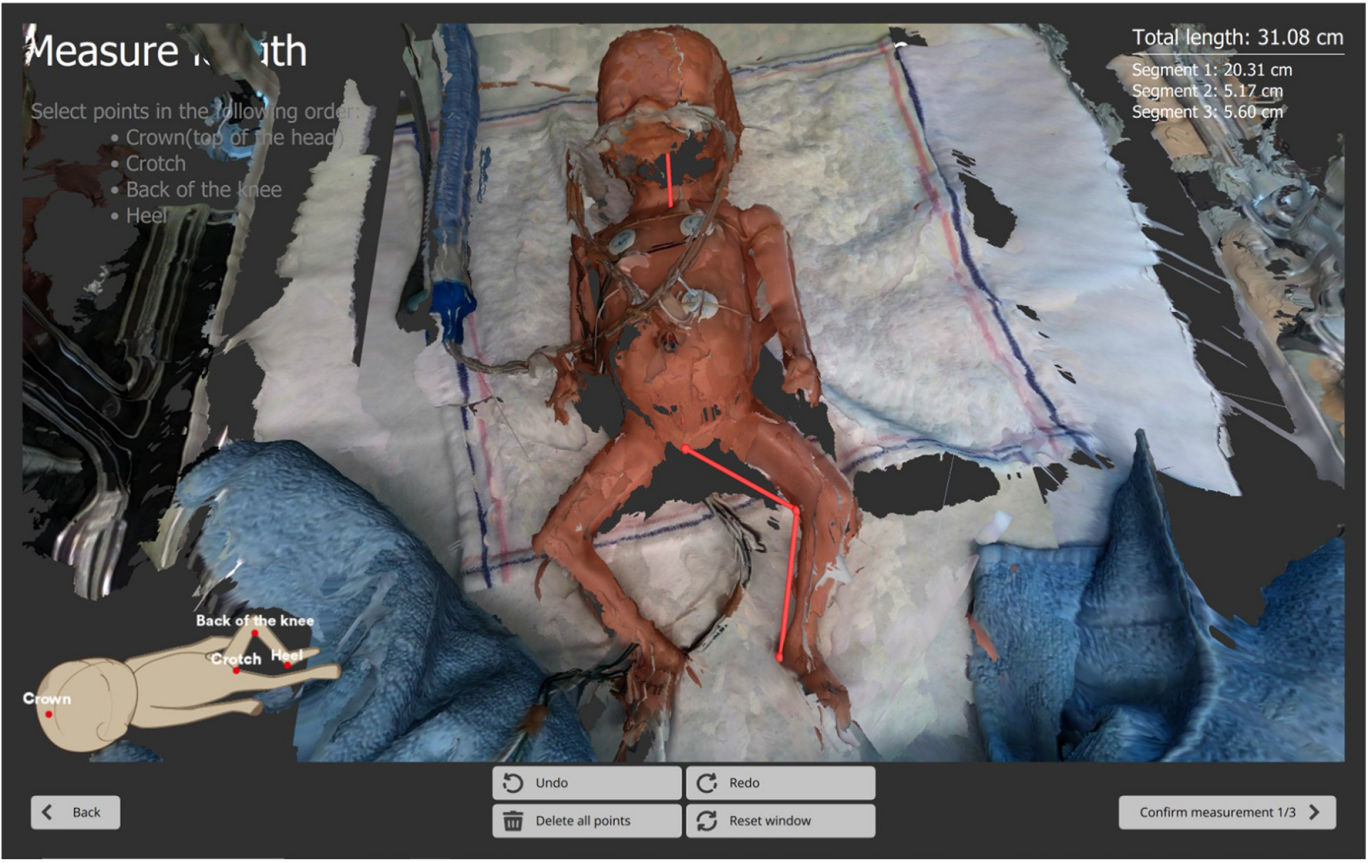
Screenshot of PreemieScanner laptop screen. Body length measurement is performed by marking anatomical landmarks on the 3D image obtained from the initial capture: crown-crotch-knee-heel. These points are marked by double-clicking, using the laptop's mouse or touch screen. Marked points appear as red dots, and the segments connecting them are highlighted with red lines. Once all points are marked, the total body length is instantly calculated and displayed in the top-right corner of the laptop screen as the sum of the lengths of three segments: crown-crotch, crotch-knee, knee-heel.

**Figure 7:**
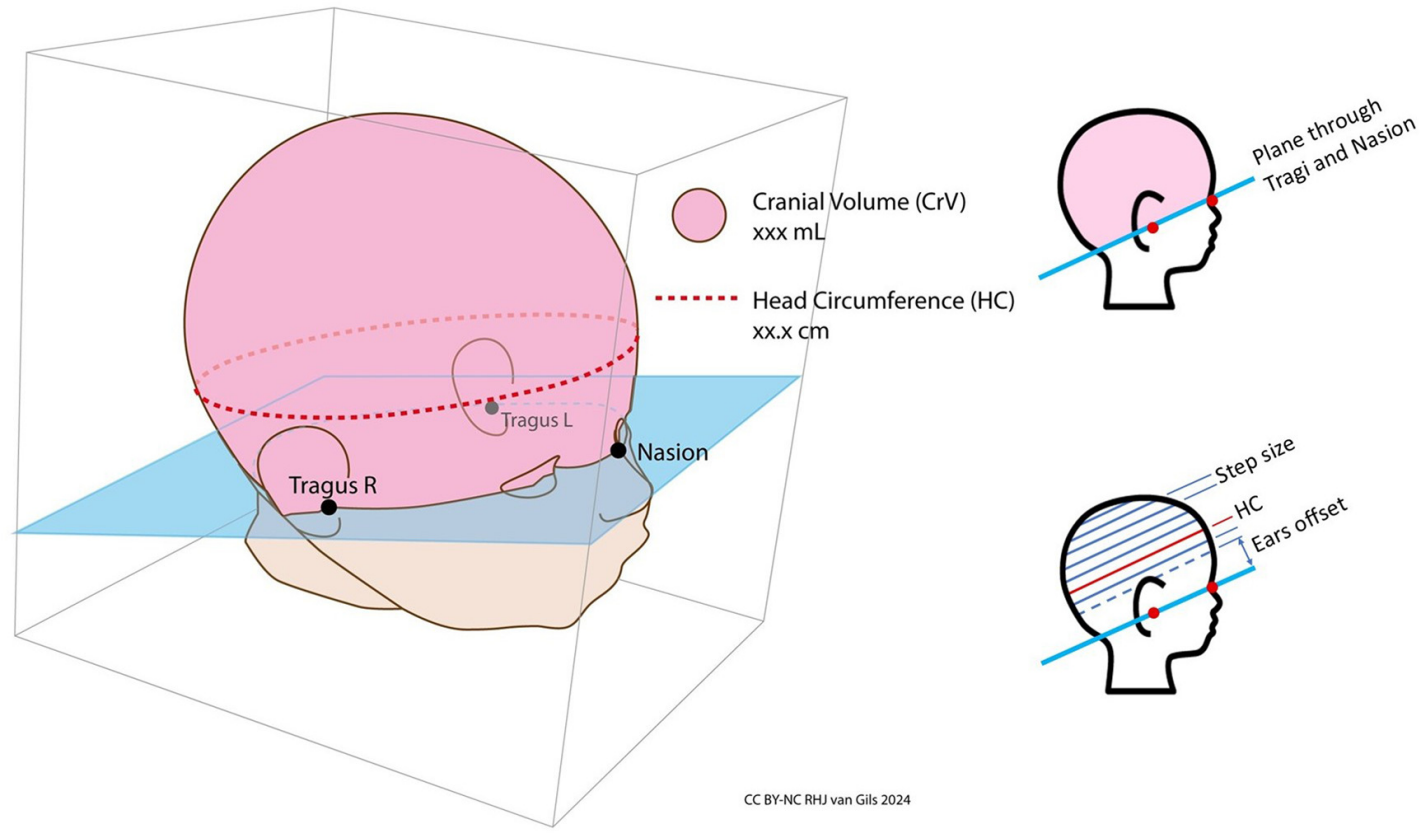
Cranial volume and head circumference. Cranial volume (CrV) is defined as the volume above the plane passing through the left tragus, right tragus, and nasion. Head circumference (HC) is measured at the largest section parallel to this plane. The software calculates HC by slicing the volume above the plane at 1 mm intervals and computing the surface area of each slice. Slicing begins slightly above the ears to exclude their additional volume, ensuring the correct, largest section is measured.

The first 3D capture, known as the “head front capture”, was taken with the head facing forward (Figure 5). To ensure an unobstructed view of the crotch, the diaper was removed for this first capture. Immediately after, and only on the first capture, the nurse manually marked anatomical points on the 3D image displayed on the laptop screen to derive BL, HC and CrV (Figure 6). For BL, four points were marked: crown, crotch, inside of the knee, and heel. For HC and CrV, three points on the head were marked: the left tragus (ear), nasion (near the nose), and right tragus (ear) (Figure 7). This marking process was repeated three consecutive times on the same 3D image, resulting in three sets of paired points for BL, and three sets for HC/CrV, aligning with standard practices of taking three measurements and calculating the mean. Each set of points was saved in a separate data file.

For the second and third captures, the nurse adjusted the head position to the left or right, closely mirroring routine care practices.

**Figure 8:**
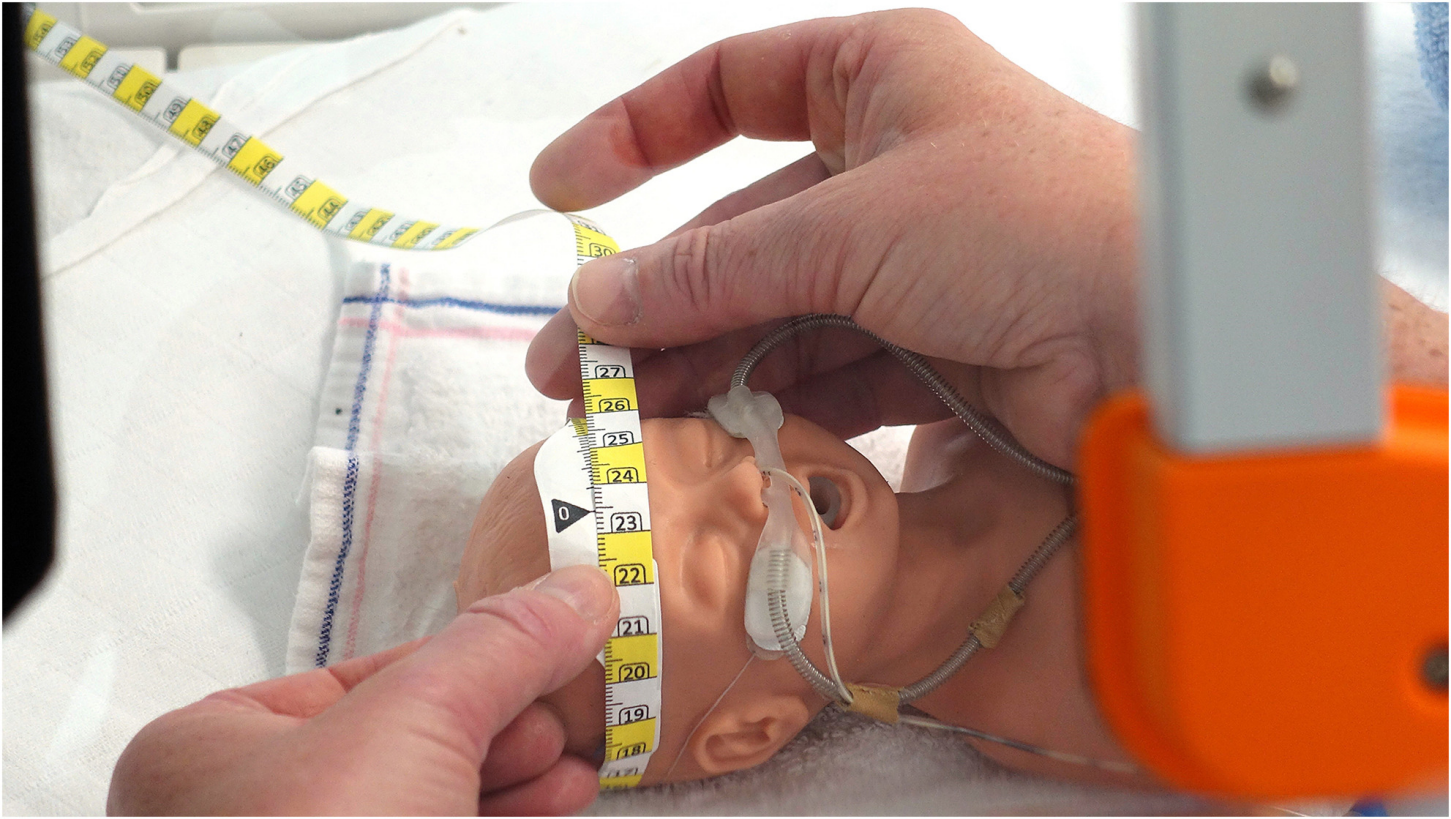
Head circumference measuring tape. The measuring tape currently utilized for measuring head circumference in the NICU participating in this study is the HappyMeasure tape. Made from Tyvek plastic paper, this tape was specifically developed by the Erasmus MC department of Create4Care, Rotterdam, The Netherlands for in-hospital use.

After completing the three captures, the nurse measured HC using the standard measuring tape of the NICU involved in this study, following the current HC measurement protocol (Figure 8). As no HC measurements had yet been obtained from the PreemieScanner, the nurses were not expected to be influenced by a HC value calculated by the PreemieScanner.

### 3.2 Merging three captures to one head

For each scan session, a 3D image of the complete head was constructed by merging the three captures into one. This 3D postprocessing process, as well as the methods to derive BL, HC/CrV values from the 3D images, are detailed in the readme file of the dataset (33) and illustrated in the demo video available on YouTube. The dataset readme file is available in Supplementary Materials Data Sheet 1.

The merging of all scan sessions was carried out collectively by four researchers. To minimize interrater variability, they participated in joint instructional and practice sessions prior to the merging process. For each scan session, all four researchers independently attempted to merge the head, resulting in four merged versions per session—one from each researcher, in Figure 4 shown as merger A, B, C, D. This approach aimed to mitigate potential bias arising from individual influence on the merging process.

### 3.3 Visual assessment of merging quality

Next step was visual assessment of the quality of the four resulting merged heads (A,B,C,D). To validly derive HC or CrV, the quality of at least one of the merges (A,B,C,D) needed to be deemed valid. This assessment was conducted collaboratively by visual examination of the generated Poisson reconstruction. This Poisson reconstruction was automatically performed by the calculation script. Poisson reconstructions showing significant deformations—typically caused by insufficient 3D data –that could clearly influence HC or CrV were considered invalid for either HC, CrV, or both measurements. The assessment was done separately for HC and CrV, because if a deformation was, for example, on top of the head, this would influence only CrV, and not HC.

### 3.4 Calculation of BL, HC and CrV

BL, HC and CrV were calculated using the PreemieScanner software's built-in script, based on the anatomical landmarks marked on the 3D image by the nurses (Figures 6, 7).

For BL, the script measures the 3D distances between points. This method allows accurate measurement, even with legs curled up, eliminating the need to straighten them. Each BL measurement is saved as data file containing the marked anatomical points as 3D (x, y, z) coordinates, along with the total BL as the sum of segment lengths.

To calculate HC and CrV, a merged head is imported in the custom-developed software via the laptop's Windows file browser. One of the three point sets, marked by the nurse during the scan session, is then imported. Calculated HC and CrV is then shown on the laptop screen and saved as in a file. In this way, HC and CrV were measured separately for all the merges that were deemed valid. A merge could be valid for HC, CrV or both. For one point set, this resulted in a set of one to four HC and/or CrV values. One, in case just one, e.g., “merge A” was deemed valid, to a maximum of four values, in case A,B,C,D were all valid. Finally, the mean was taken as resulting HC or CrV measurement of that particular scan session and point set.

Anatomical landmarks for HC and CrV were marked on the 3D image from the first capture, rather than on the fully merged head, for two main reasons. First, the full-color detail of a single capture provided greater morphological clarity compared to the merged head, where three full-color 3D images blended into a “camouflage look”, making the tragus and nasion harder to distinguish. Second, requiring nurses to mark points on the merged head after the merging process would have necessitated their return later, a challenge given their demanding schedules.

### 3.5 Data analysis

Assessment of clinical usability of the PreemieScanner is twofold: (1) can the PreemieScanner measure the weekly growth of preterm infants with sufficient clinical accuracy and precision? To answer this, we need to define the smallest weekly growth (or: weekly increase) the device should reliably detect. The smallest weekly growth values for BL, HC and CrV can be translated to clinically allowed precision limits the device should comply with. (2) Is the PreemieScanner feasible to fit in NICU routine care? Feasibility is quantified by the percentage of scan sessions leading to usable measurement values. The success rate of conducting a scan session should be high enough to justify implementation in clinical practice.

#### 3.5.1 Success rate of scan sessions

The success rate of scans sessions was determined as the percentage of successful sessions. A successful session led to a BL, HC, or CrV measurement. For BL, measurement results were instantly shown during the scan session, when the nurse marked the anatomical landmarks to derive BL. For HC or CrV, a successful measurement depended on the visual assessment of merging quality, where at least one of four merged heads should be deemed valid for either HC or CrV calculation. In case none of four merges were valid, that scan session was counted as unsuccessful for either HC, CrV or both. As described earlier, visual assessment of merging quality was done separately for HC and CrV: a merge could be valid for HC but invalid for CrV.

#### 3.5.2 Accuracy and precision

For this study, accuracy and precision were evaluated using the measurement error (ME), defined as the deviation of a measured value from the ground truth (GT) values. The ground truth values represent the fixed, true dimensions of the dolls. The method for establishing GT is described in a later section. Accuracy reflects trueness—the closeness of the measured values to the true value of the object measured. Precision, also referred to as reliability, refers to the consistency of a measurement method, indicating how closely repeated measurements of the same object agree (34). A study into taxonomy of measuring instruments defines reliability as “the degree to which the measurement is free from measurement error” (35, 36), which implies both agreement with the true value and between measurements. In Supplementary Materials Data Sheet 2—Accuracy and precision, fictive data plots illustrate the interpretation of accuracy and precision from the MEs.

Accuracy is expressed as the mean ME if the distribution is normal or the median ME if not. Precision is reflected by the spread of ME, measured by the error interval. The overall accuracy of the PreemieScanner for BL, HC and CrV was determined by the combined overall mean or median ME for all three dolls. Precision, reflecting the spread of the MEs, was quantified using the minimum-maximum (min-max) range and 95% interval of that range, which captures 95% of observed MEs, excluding extreme outliers. The spread of MEs of repeated measurements can be regarded as the test-retest reliability. For non-normally distributed MEs, the 95% interval was defined as Q2.5—Q97.5, while for normally distributed MEs, it was expressed as the mean +/− 1.96*SD, covering 95% of the data. The consistency of measurements between nurses, the interrater variability and with the three measurements of one nurses—the intrarater variability, can be quantified by the intraclass correlation coefficient (ICC). Data analysis of nurses' inter- and intrarater ICC is presented in the Supplementary Materials Data Sheet 2—Nurses' Inter- and Intrarater variability, as this does not directly contributes to our main question.

Metrics can be expressed either in absolute measurement units (cm for BL and HC, ml for CrV), or as percentage of the GT value. The percentage approach is preferred when measuring objects of different sizes, in our case three dolls with slightly different sizes, as an ME of 0.1 cm has different implications for an object of 10 cm vs. one of 100 cm. Therefore, in our analysis, all measured values were converted to proportional values, expressed as percentages relative to the GT values of each individual doll.

#### 3.5.3 Ground truth values

The GT values were obtained from precise 3D scans of the dolls' heads, captured using a medical-grade scanner known for its high accuracy (3dMD head system, purchased in 2020 from 3dMD, Atlanta, USA, with a manufacturer-claimed shape accuracy of 0.2 mm). One 3dMD capture provides a 360-degree round 3D image of the doll. BL, HC and CrV were measured from these scans using the same calculation scripts describe above. To establish the GT values, anatomical 3D points were identified based on multiple points sets marked by four different raters. Each raters marked three sets of anatomical 3D points for each doll for both BL and HC/CrV, resulting in x, y, z coordinates for each 3D point. The average x, y, z coordinates across all sets were calculated and used to determine GT values for BL, HC and CrV for each doll. All data used to establish GT values are available in the online dataset (33).

#### 3.5.4 Clinically allowed precision limits

The clinically allowed precision limits were set to match the smallest weekly growth increases observed in a P50 preterm infant for BL, HC, and CrV. This approach resulted in precision limits of +/− 0.4 cm for BL, +/− 0.3 cm for HC, and +/− 12 ml for CrV. These limits are applied as margins below and above the median or mean ME for each doll. For instance, if the medium or mean HC ME is 1.0 cm, a clinically acceptable ME should fall within the range of 0.7–1.3 cm, given the precision limit of 0.3 cm. We translated these absolute precision limits (in cm, ml) into proportional limits (%) relative to each doll's GT value.

The ability to detect clinical relevant change over time—with sufficient reliability, is also referred to as the responsiveness of a device (34, 35). Responsiveness relates to the smallest clinical relevant change the instrument should detect. For the PreemieScanner scanner, this threshold is determined by the smallest expected weekly growth increase in BL, HC and CrV, as the scanner is intended for weekly growth measurements (27, 37, 38). These smallest weekly increases are translated into clinically allowed precision limits.

To determine these smallest weekly increases, we extracted growth data from multiple data sources, including growth reference charts and published studies (25–29, 39–42), as summarized in Table 1. Detailed tables of the extracted weekly growth data are available in the Supplementary Materials Data Sheet 3—BL_HC_CrV weekly increase. The precision limits were established by calculating the mean of the smallest growth values across all data sources, for infants with GA up to 40 weeks. Table 2 presents these precision limits as +/− intervals, indicating the range of the clinically allowed ME: MEs falling within these limits are considered clinically acceptable for weekly, longitudinal growth monitoring.

**Table 1:** P50 minimum values for weekly increase of BL, HC and CrV. Data extracted from growth data of multiple data sources, in P50 preterm infants up to 40 weeks GA.

Data Source	BL [cm]	BL proportional [%]	HC [cm]	HC proportional [%]	CrV [ml]	CrV proportional [%]
Altigani et al. (41)	1.0	2.1	0.6	1.7	–	–
Babson (42)	0.6	1.4	0.5	1.5	–	–
Dieks et al. (29)	–	–	0.7	2.0	32.0	6.5
Horemuzova et al. (39)	0.6	1.6	0.5	1.7	–	–
RCPCH (26)	0.7	1.4	0.4	1.0	–	–
Riddle et al. (40)	1.0	2.1	0.5	1.6	–	–
Vermeulen et al. (28)	–	–	–	–	16.5	4.0
Villar et al. (27)	1.3	3.1	0.9	3.1	–	–
Young et al. (25)	0.8	2.6	0.6	2.6	–	–
Mean minimum weekly increase, over all data sources^a^	0.8	2.0	0.6	1.9	24.3	5.3
Translation to “+/−” precision limit:^b^	0.4	1.0	0.3	1.0	12	2.6

BL, body length; HC, head circumference; CrV, cranial volume; GA, gestational age.

^a^ Mean of the values above.

^b^ Mean minimum weekly increase values divided by 2, as the +/- precision limit, representing an interval around the true (ground truth) value in which a measured value should lie to be clinically useful.

**Table 2:** Minimal preterm P50 values for weekly increase of body length, head circumference and cranial volume, translated to precision limits. Because the three dolls had slightly different sizes, to allow an even comparison between the MEs per doll, we translated the absolute precision limits to proportional limits, relative to the GT value of the specific doll.

Body size parameter	Minimal absolute weekly increase	Absolute precision limits +/−	Ground truth doll 1	Proportional limits +/− doll 1	Ground truth doll 2	Proportional limits +/− doll 2	Ground truth doll 3	Proportional limits +/− doll 3
Body length	0.8 cm	+/**−** 0.4 cm	31.9 cm	+/**−** 1.3%	33.4 cm	+/**−** 1.2%	31.4 cm	+/**−** 1.3%
Head circumference	0.6 cm	+/**−** 0.3 cm	25.2 cm	+/**−** 1.2%	24.1 cm	+/**−** 1.2%	27.8 cm	+/**−** 1.1%
Cranial volume	24 ml	+/**−** 12 ml	221.2 ml	+/**−** 5.4%	193.4 ml	+/**−** 6.2%	307.0 ml	+/**−** 3.9%

In a NICU, preterm growth is typically monitored by plotting weekly measured values of weight, BL, and HC against gestational age. In Supplementary Materials Data Sheet 2—Accuracy and Precision, fictive HC plots illustrate the relation between measured growth and actual growth. These measured growth curves are compared to reference growth charts, which display percentile-based growth patterns for weight, BL, and HC (26, 27). If the error interval of the instrument exceeds the actual growth increase between two measurements, there is a risk of inaccurately observing ’shrinkage' when normal growth is occurring. To prevent this, the precision of the measuring instrument must have an error interval that is smaller than, or at most, equal to the smallest expected weekly growth increase.

### 3.6 Statistical analysis

Data were categorized based on the type of measurement (BL, HC, CrV) and the specific doll measured (Doll 1, 2 or 3). For HC and CrV, data were further divided based on the placement of the Optiflow tube (front or back). Optiflow tube placement had no impact on BL measurements. Additionally, HC data were analyzed separately by the measurement method used: PreemieScanner or measuring tape. Normality of distributions was assessed using the Shapiro–Wilk normality test, complemented by point plots.

Differences between the overall mean or median ME and the GT—essentially testing for an ME of zero—were evaluated using one sample *T*-tests for normally distributed data and Wilcoxon rank-sum tests for non-normally distributed data. To visually depict accuracy and precision, we generated point plots illustrating MEs for each measurement type. Because the three dolls slightly varied in size, we expressed the data proportionally relative to each doll's GT value. This approach ensured that the precision limit interval appeared proportional to the size of each doll, enabling a fair visual comparison across all measurements.

In the Supplementary Materials Data Sheet 2, consistency of measurements within one nurse is presented by the intra class correlation (ICC) for intrarater variability/reliability, and agreement between different nurses by the ICC for interrater variability/reliability.

In addition, the statistic metric 'smallest detectable change' (SDC) was used as estimate of the minimum change the device can interpret as a real difference, beyond measurement error. In other words, the minimum change that must occur for an observer to be confident (with 95% certainty) that the change is not due to measurement error alone. The SDC, expressed in cm or ml can be directly compared to the minimal absolute weekly increases: 0.8 cm for BL, 0.6 cm HC and 24 ml CrV. If SDC is smaller than the minimal weekly increase, the PreemieScanner can reliably measure weekly growth, the smaller the SDC, the better the PreemieScanner performs. In statistics, SDC is defined as SDC = 1.96*√2*SEM, with SEM as the Standard Error of Measurement. SEM, in statistics defined as SEM = SD*sqrt(1−ICC), or SD*sqrt(1−correlation) is a metric for understanding the spread of errors, typically in comparing one device to another, in other words precision of measurements. However, as we, in our case, comparing measurements from a device to a fixed ground truth (with, theoretically, no measurement error), SEM is equal to the standard deviation of the MEs, as the entire variability in the deviation to the ground truth (GT) is attributed to the device, in other words SEM = SD(ME).

Statistical analyses were performed using R Statistical Software Version 4.4 (R Foundation of Statistical Computing, Vienna, Austria), with significance set at *p* < 0.05 (two-tailed). R-scripts and data tables used for statistical analyses can be found in Supplementary Materials Data Sheets 4–6.

## 4 Results

### 4.1 Success rate of scan sessions

**Figure 9:**
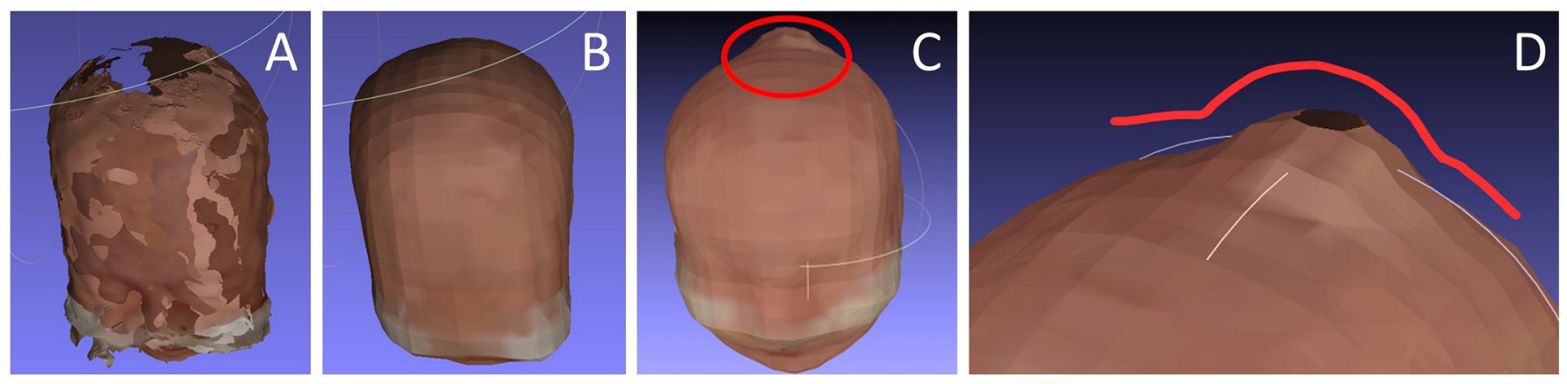
Examples of merged heads. **(A)** Result after the manual merging process, showing areas with missing 3D data; **(B)** The same head after Poisson reconstruction performed by the calculation script; **(C)** An example of a failed Poisson reconstruction with severe, unrealistic deformations due to excessive missing 3D data. **(D)** Close-up view of the deformation in C.

Thirty-five nurse performed a scan session. The 35 scan sessions resulted in a 100% success rate for BL and HC measurements and an 80% success rate (28/35) for CrV measurements. Seven sessions were unsuccessful in measuring CrV. In these seven sessions, all of four merged heads were deemed invalid for CrV calculation. This was due to missing 3D data, leading to significant deformations in the Poisson reconstruction process, resulting in failed or unrealistic CrV calculations. Figure 9 presents examples of merged head scans, including a failed reconstruction.

### 4.2 Measurements within precision limits

Table 3 summarizes the number of measurements with MEs falling within the established precision limits for all dolls combined, for Optflow front. These counts and proportions were calculated relative to the precision limits adjusted for each doll's medium or mean ME. For BL with PreemieScanner, overall, all dolls and Optiflow front/back combined, 40% of MEs were within precision limits. For HC with PreemiesScanner, this overall percentage is 91%, and for HC with measuring tape 86%. CrV with PreemieScanner with 93% has the highest overall percentage of MEs within limits. Table 4 provides similar data broken down for each individual doll. Detailed statistic tables are available in Supplementary Materials Data Sheet 7—Statistics Tables. In the data plots presented in the following sections, the precision limits are visually indicated, allowing for easy identification of measurements that fall within these limits.

**Table 3:** Measurements with ME within precision limits & smallest detectable change (SDC), for all dolls combined. Measurements with ME within limits are considered clinically useful for weekly growth monitoring. The more measurements with MEs falling within limits, the better. Body length (BL), head circumference (HC) and cranial volume (CrV) measurements, conducted with PreemieScanner and standard measuring tape. Grouped by Optiflow tubes guided to front or back, and front/back combined (overall performance). For body length no split-up is made for Optiflow Front/Back, as Optiflow method does not influence length measurements. The metric SDC is the smallest change a device can reliably detect. In our case, it should be smaller than the smallest expected weekly increase.

All dolls combined	Body length PrSc					HC PrSc					HC Tape					CrV PrSc				
	within limits	n	%	SDC^a^ [cm]	SDC^b^ in % WI	within limits	n	%	SDC [cm]	SDC in % WI	within limits	n	%	SDC [cm]	SDC in % WI	within limits	n	%	SDC [ml]	SDC in % WI
Optiflow front	NA	NA	NA	2.3	293%	51	57	89%	0.5	89%	50	57	88%	0.6	96%	31	36	86%	30.2	126%
Optiflow back	NA	NA	NA	4.0	495%	43	46	93%	0.9	147%	40	48	83%	0.9	148%	45	46	98%	15.4	64%
Overall	42	105	40%	3.2	399%	94	103	91%	0.7	118%	90	105	86%	0.7	124%	76	82	93%	22.9	96%

HC, head circumference; PrSc, PreemieScanner; CrV, cranial volume; Tape, measuring tape; WI, weekly increase.

^a^ SDC, Smallest Detectable Change = 1.96*sqrt(2)*SEM, SEM = Standard Error of Measurement = SD(ME) when ME is relative to fixed ground truth. Lower values are better.

^b^ SDC in % of expected smallest weekly increase (0.8 cm for BL, 0.6 cm for HC, 24 ml for CrV). Percentage lower than 100% = clinically useful. Lower values are better.

**Table 4:** Body length (BL), head circumference (HC) and cranial volume (CrV) measurements, conducted using PreemieScanner and currently used measuring tape. Results are grouped by the body size parameter (BL, HC, CrV), doll, measuring device, Optiflow tubes guided to back or front of the head. Results are expressed in the proportional measurement errors (ME), calculated relative to the ground truth (GT) of each doll. Accuracy is expressed as the mean or median deviation from the GT, depending on normality of the ME distribution. Precision can be interpreted by the min-max interval, the total range of MEs, and the 95% reference interval, covering 95% of MEs, so excluding 5% outliers. Measurements with ME within limits are considered clinically useful for weekly growth monitoring: the more measurements with MEs falling within limits, the better device's precision.

Measurement			Precision limits		Accuracy	Corrected precision limits^b^		ME Range			Precision by 95% ME Reference Interval			MEs within corrected precision limits^c^		
Body size parameter, Device, Optiflow method	Doll	Normality^a^	lower limit [%]	upper limit [%]	Median/mean deviation from GT [%]	lower limit [%]	upper limit [%]	min	max	min-max interval [abs %]	Q2.5	Q97.5	95% interval [abs %]	Within corrected limits	n	Proportion within limits [%|
BL, PrSc	1	Yes (*p* = 0.55)	−1.3	1.3	−3.8	−5.1	−2.6	−6.9	0.0	6.9	−7.1	−0.5	6.6	19	27	70
	2	No (*p* = 0.02)	−1.2	1.2	−1.2	−2.4	0.0	−4.2	5.4	9.6	−3.8	5.2	9.0	12	39	31
	3	No (*p* < 0.001)	−1.3	1.3	−5.1	−6.4	−3.8	−8.6	14.0	22.6	−8,0	9.5	17.5	11	39	28
HC, Tape, Optiflow Front	1	No (*p* < 0.001)	−1.2	1.2	−0.8	−2.0	0.4	−2.0	0.0	2.0	−1.7	0.0	1.7	26	27	96
	2	Yes (*p* = 0.13)	−1.2	1.2	−1.8	−3.0	−0.5	−4.6	0.4	5.0	−4.8	1.2	6.0	7	12	58
	3	No (*p* = 0.002)	−1.1	1.1	−0.7	−1.8	0.4	−1.1	0.7	1.8	−1.1	0.3	1.3	17	18	94
HC, Tape, Optiflow Back	1	Yes (*p* = 0.05)	−1.2	1.2	−1.9	−3.1	−0.7	−5.6	1.2	6.8	−6.3	2.6	8.9	7	12	58
	2	Yes (*p* = 0.10)	−1.2	1.2	−1.6	−2.8	−0.3	−2.9	0.4	3.3	−3.6	0.5	4.1	12	15	80
	3	No (*p* < 0.001)	−1.1	1.1	−1.1	−2.2	0.0	−1.4	−0.4	1.1	−1.4	−0.5	0.9	21	21	100
HC, PrSc, Optiflow Front	1	No (*p* = 0.009)	−1.2	1.2	0.0	−1.2	1.2	−1.2	1.2	2.4	−1.2	1.2	2.4	21	27	78
	2	No (*p* = 0.02)	−1.2	1.2	0.4	−0.8	1.7	−0.4	0.8	1.2	−0.4	0.8	1.3	12	12	100
	3	No (*p* = 0.002)	−1.1	1.1	−0.7	−1.8	0.4	−1.1	−0.4	0.7	−1.1	−0.4	0.7	18	18	100
HC, PrSc, Optiflow Back	1	No (*p* = 0.003)	−1.2	1.2	0.0	−1.2	1.2	−0.8	0.4	1.2	−0.8	0.4	1.2	12	12	100
	2	No (*p* < 0.001)	−1.2	1.2	1.2	0.0	2.5	0.4	1.7	1.3	0.4	1.5	1.1	13	13	100
	3	No (*p* < 0.001)	−1.1	1.1	−0.7	−1.8	0.4	−3.2	0	3.3	−3.2	0	3.2	18	21	86
CrV, PrSc, Optiflow Front	1	Yes (*p* = 0.29)	−5.4	5.4	−3.4	−8.8	2.0	−7.3	−0.2	7.1	−8.2	1.3	9.5	12	12	100
	2	Yes (*p* = 0.17)	−6.2	6.2	1.5	−4.7	7.7	−1.6	7.7	9.2	−5.6	8.6	14.2	6	6	100
	3	No (*p* = 0.005)	−3.9	3.9	−1.8	−5.7	2.1	−8.1	9.1	17.1	−7.5	8.7	16.2	13	18	72
CrV, PrSc, Optiflow Back	1	Yes (*p* = 0.13)	−5.4	5.4	−1.3	−6.8	4.1	−4.7	3.3	8.0	−6.9	4.2	11.1	12	12	100
	2	Yes (*p* = 0.29)	−6.2	6.2	−0.6	−6.8	5.6	−4.0	3.6	7.6	−4.0	2.9	6.9	13	13	100
	3	No (*p* < 0.001)	−3.9	3.9	−1.7	−5.6	2.2	−7.6	0.6	8.2	−5.8	0.5	6.2	20	21	95

BL, body length; HC, head circumference; CrV, cranial volume; ME, measurement error; PrSc, PreemieScanner; Tape, measuring tape.

^a^ Shapiro–Wilk test for normality.

^b^ Corrected relative to the median/mean deviation.

^c^ Number of measurement errors, out of all measurements, that lie within the corrected precision limits.

### 4.3 Smallest detectable change

Table 3 presents both SDC in absolute values as well as proportion to the expected absolute smallest weekly increases for BL, HC and CrV. In addition to the percentage of measurements with ME within precision limits, the SDC, expressed in cm for BL and HC, and ml for CrV, can be directly compared to the expected smallest weekly increase, as a measure for clinical suitability for measuring weekly growth. The SDC should be smaller than the expected change in order to be (at least) 95% confident that measured increase is actual growth and not only caused by measurement error. For all dolls and Optiflow front and back combined, proportional SDC for BL is 400%, for HC-tape 124% and HC-PrSc 118%, and Crv 96%. If we group by Optiflow method, the Optiflow front has a significantly lower SDC compared to back, for BL (293/495%), HC-tape (89/147%) and HC-PrSc (96/148%). For CrV, Optiflow front scores higher than back (96/148%).

### 4.4 Overall accuracy for BL, HC and CrV

Table 5 presents the overall accuracy—for all three dolls combined, for BL, HC, and CrV, expressed as the mean or median measurement error (MME). MME below zero indicate that measured values, on average, were lower than GT. No significant differences from GT were found for HC with PreemieScanner, with MME 0.0% (*p* = 0.63) for Optiflow front, and −0.4% (*p* = 0.91) for Optiflow back. MME HC with tape did significantly differ from GT: −0.8% (*p* < 0.001) Optiflow front, −1.1% (*p* < 0.001) Optiflow back. BL measurement with PreemieScanner did significantly differed from the GT, with MME Optiflow front/back −3.3% (*p* < 0.001). Lastly, CrV with PreemieScanner significantly differed frm GT with Optiflow front −1.8% (*p* = 0.01) and Optiflow back −1.3% (*p* < 0.001).

**Table 5:** Overall accuracy for body length, head circumference, cranial volume, of measurements conducted with PreemieScanner and standard measuring tape. Grouped by Optiflow tubes guided to front or back. For body length no split-up is made for Optiflow Front/Back, as Optiflow method does not influence length measurements.

Optiflow method	Median or mean measurement error for all dolls combined [%]							
	Length [%]	*p*-value^a^	HC PrSc [%]	*p*-value^a^	HC Tape [%]	*p*-value^a^	CrV [%]	*p*-value
Optiflow Front	−3.3	<0,001	0.0	0,63	−0.8	<0,001	−1.8	0,01
Optiflow Back			−0.4	0,91	−1.1	<0,001	−1.3	<0,001

HC, head circumference; CrV, cranial volume; PrSc, PreemieScanner, Tape, measuring tape.

^a^ *T*-test if normally distributed, Wilcoxon signed rank test if not.

### 4.5 Accuracy and precision of BL measurements

**Figure 10:**
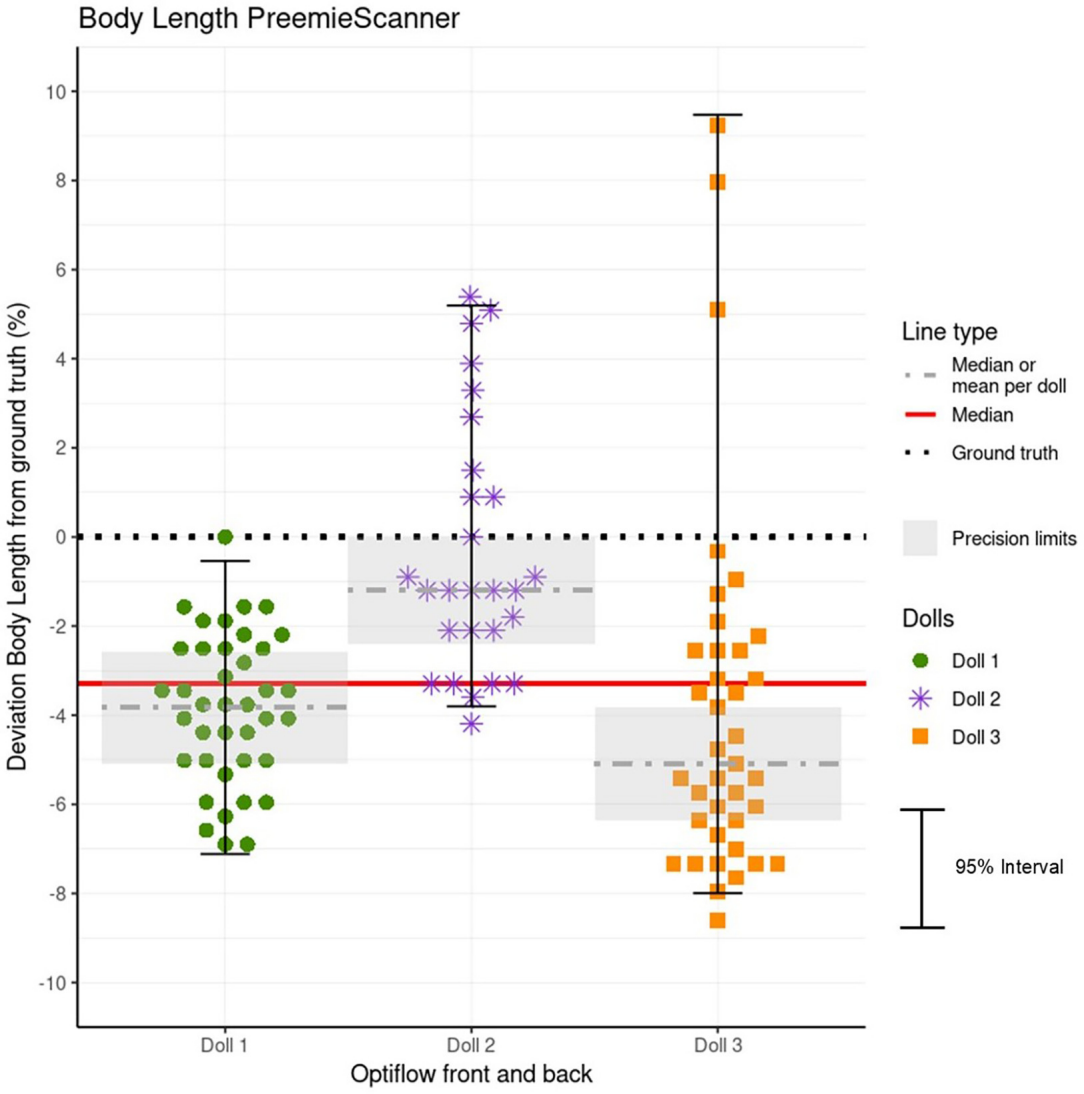
Data plot of BL measurement errors (MEs) for each doll. The MEs represent the deviation of BL measurements from the ground truth (GT) values, expressed as a percentage of the GT. Each doll's MEs are plotted individually, with precision limits corrected relative to the doll's mean or median ME. MEs within these limits, highlighted by a grey box, are clinically acceptable for weekly growth monitoring. Additionally, a red line indicates the overall accuracy represented by the combined mean or median ME for all three dolls. Because the Optiflow attachment method (Front or Back) did not affect BL measurements, data from both methods are combined and presented as a single group.

Statistics for BL measurement errors, grouped by doll are presented in Table 4, with corresponding MEs visualized in Figure 10. Shapiro–Wilk normality tests indicated that only the BL MEs of doll 1 showed normal distribution. For all dolls, BL was consistently underestimated, with the mean or median ME falling below the GT. Largest spread of MEs, so lowest precision, are in doll 3, with a 95% ME interval of 17.5%, resulting in 28% of MEs within limits. The plot shows three outliers in doll 3, resulting in a large ME interval. Measurements of doll 1 showed highest precision, with 95% interval of 6.6% and 70% within limits.

### 4.6 Accuracy and precision of HC measurements

**Figure 11:**
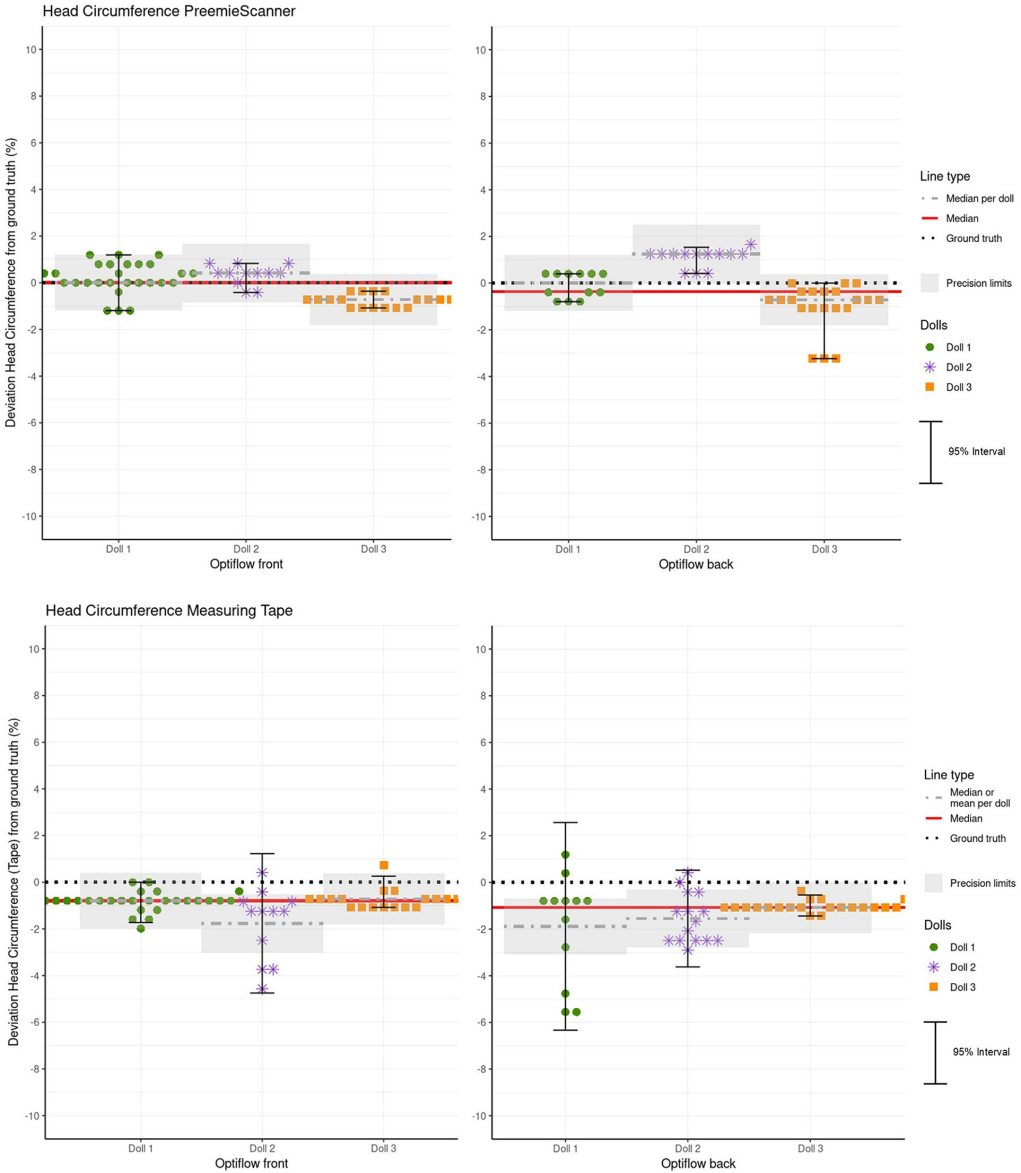
Hc measurement errors (MEs) for both the PreemieScanner and the measuring tape method for each doll, for Optiflow front and back. The plot superimposes the precision limits per doll, corrected relative to the mean or median. MEs within these limits are considered clinically acceptable. Additionally, a red line indicates the overall accuracy represented by the combined mean or median for all three doll.

Table 4 and Figure 11 present the MEs for both PreemieScanner and the measuring tape, across all dolls, separated by Optiflow attachment positions (Front and Back). For PreemieScanner, highest precision is shown in doll 3 for Optiflow front, with 95% ME interval/within limits of 0.7%/100% and for Optiflow back in doll 2 with 1.1%/100%. For tape, highest precision, for both Optiflow front and back, is shown in doll 3, with 95% ME interval/within limits of 1.3%/94% Optiflow front and 0.9%/100% for Optiflow back.

### 4.7 Accuracy and precision of CrV measurements

**Figure 12:**
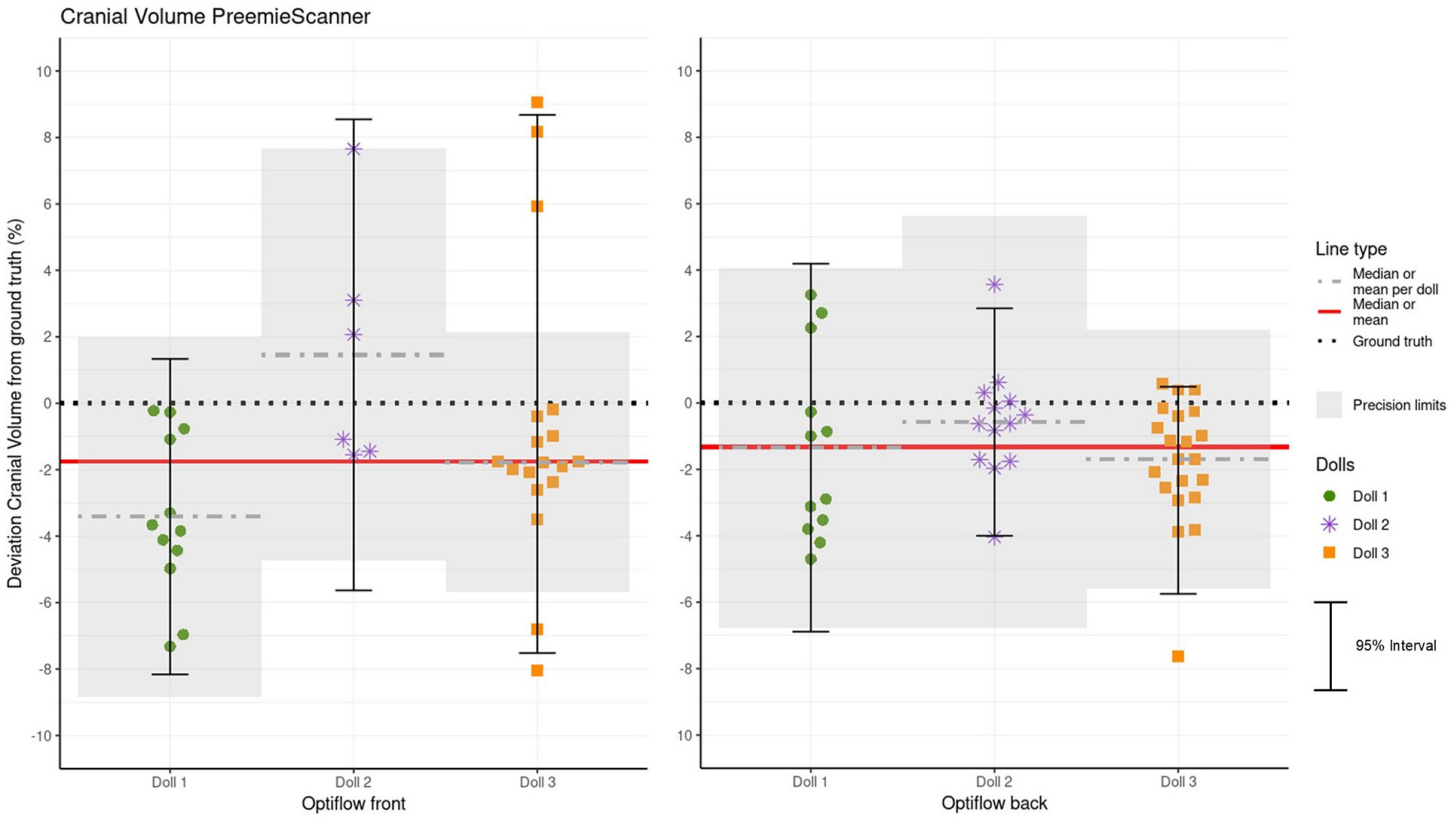
Crv measurement errors (MEs) per doll, separated by Optiflow front and back positions. Precision limits, corrected relative to each doll's mean or median, are superimposed on the plot. MEs within these limits are considered clinically acceptable. Additionally, a red line represents the overall accuracy, indicated by the combined mean or median for all three dolls.

Table 4 presents the statistics for CrV measurement errors, grouped by doll and Optiflow method. Figure 12 visualizes the distribution of these errors. Spread in CrV MEs is relatively larger than in HC MEs. Highest CrV precision is shown in doll 1 for Optiflow front, with 95% ME interval/within limits of 9.5%/100% and for Optiflow back in doll 3 with 6.2%/95%. It should be noted that, although for Optiflow back doll 2 ME range is smaller than doll 3, but due to the statical calculation of the 95% reference intervals based on normality (Q2.5–97.5 vs. +/−1.96*SD), the 95% interval of doll 2 is smaller. Considering MEs within limits, doll 3 scores higher with 100% vs. 95% for doll 2.

Most CrV values measured by the PreemieScanner are below the GT. For all individual rater groups, as well as the combined rater data, the mean CrV measurements deviate significantly from the GT.

## 5 Discussion

The current study evaluated the clinical usability of body length (BL), head circumference (HC), and cranial volume (CrV) measurements using the novel PreemieScanner 3D scanner in a simulated NICU setting. Nurses conducted scan sessions with the PreemieScanner, scanning three prepared dolls, and also measured HC using the currently used measuring tape. Usability was assessed based on two key factors: (1) the success rate of scans, defined as the percentage of scan sessions that resulted in successful calculations of BL, HC, or CrV, and (2) the percentage of measurements falling within clinically acceptable precision limits, which were set according to the smallest expected weekly growth of a P50 preterm infant.

In terms of scan success, integrating PreemieScanner scan sessions with routine care is clinically feasible, with 100% success in measuring BL and HC, and 80% success for CrV. Regarding clinically sufficient precision for weekly measurements, 93% of CrV measurements, 91% of HC measurements, and 40% of BL measurements fell within the defined precision limits. Notably, for HC, the PreemieScanner outperformed the currently used measuring tape in both precision and accuracy. For PreemieScanner HC measurements, in addition to high precision, the mean/median measurements did not significantly differ from the GT, making them highly accurate for clinical use. Detailed findings are discussed by topic below.

### 5.1 Success rate of scan sessions

In our study, the success rate of scan sessions was 100% for measuring BL and HC, and 80% for measuring CrV, all of which we consider sufficient for clinical use. In contrast, the MONITOR3D validation study (30) reported a scan success rate of 45%, which we considered too low for clinical use. For further comparison, Andrews et al. conducted a similar study measuring HC and BL of preterm infants using a 3D camera, reporting a success rate of 76% (43).

The 20% missing CrV measurements were all caused by “invalid 3D merges”. This means that all of four merge attempts resulted in a 3D image with large deformations, not present in the real doll's head, caused by missing 3D data in the capture. Interestingly, these deformations would only result in biased CrV but would not influence HC. In future research, we could calculate CrV from the invalid merges, to see if we can find other 3D postprocessing methods that still can cope with missing 3D data in a valid way, resulting in reliable merged heads. For this study this fall outside our scope.

### 5.2 Measurements within precision limits

Regarding clinically sufficient precision for weekly measurements, 93% of CrV measurements, 91% of HC measurements, and 40% of BL measurements fell within our set precision limits. Unfortunately, this performance cannot be directly compared to that of the PreemieScanner's predecessor, MONITOR3D, as usability in that study was not expressed as “percentage within precision limits”. However, we can compare the performance of PreemieScanner and MONITOR3D using the MME for accuracy and the 95% reference intervals for precision. In these comparisons, all values favor PreemieScanner, particularly for HC. Specifically, for HC, the absolute MME (accuracy) and the 95% interval (precision) as percentages of the GT were 0.6/2.9 for MONITOR3D, compared to 0.2/1.7 PreemieScanner. For CrV, the values were 2.3/10.8 for MONITOR3D, vs. 1.5/10.7 for PreemieScanner.

The neonatologists involved in this study emphasized that, for growth monitoring, the curve of the measured values (including measurement errors) should closely align with the curve of the actual values. Achieving this requires small error intervals, meaning narrow precision limits. The proximity of the measured curve to the true curve is considered more important than the position of the measured curve relative to the true percentile reference curve, which is determined by the accuracy of the instrument. This concept is visualized using fictive plots in Supplementary Materials Data Sheet 2—Accuracy and Precision.

For this study, we set the precision limits of the measuring device to match the smallest anticipated weekly growth. This prevents the odd situation that “shrinkage” is recorded, when in fact normal growth is occurring. Determining these precision limits is somewhat arbitrary, as there are no established clinical or engineering standards for measuring instruments to serve as a reference.

When examining the specified accuracy (expressed as the +/− error margin) of commercially available medical measuring instruments from well-known manufacturers, we observe relatively large error margins. For example, a BL measuring device for infants document lists an accuracy of +/− 1 cm (44), and a neonatal HC measuring tape claims an error margin of +/− 0.5 cm (45).

Our precision limits were based on BL, HC, and CrV weekly growth velocities, aggregated from various data sources identified for this study. The BL and HC growth data, supported by sufficient sources, can be considered representative of a large portion of the preterm population. However, for preterm CrV data, only two sources were identified, with sample sizes of *n* = 26 and *n* = 1,703 (28, 29).

We identified one other study that measured preterm BL and HC using 3D technology and used the error interval as an indicator of clinical usability (43). The authors presented Bland-Altman plots with 95% limits of agreement ranging from +2.1 to −1.8 cm, noting that “ … such a difference is likely to be clinically important, leading to mis-allocation of the correct growth percentile, especially in smaller infants”.

### 5.3 Smallest detectable change

The SDC values, in addition to the percentage of measurements within set precision limits, provide insight in the clinical usability of the device, as estimate of the minimum change the device can confidently observe (with 95% certainty) as a real difference, and not due to measurement error alone. The SDC values were directly compared to the absolute smallest weekly increases, used to set our precision limits: 0.8 cm for BL, 0.6 cm HC, and 24 ml for CrV. The SDCs as proportion of expected smallest weekly growth are in line with the percentages of measurements within limits, which is not remarkable, as the computational approach is similar.

### 5.4 Head circumference measurement compared between measuring tape and PreemieScanner

The PreemieScanner outperforms the measuring tape in both precision and accuracy. Additionally, the PreemieScanner is expected to be less stressful for infants compared to the measuring tape, making it a more preferable option. For the Optiflow Back, the measuring tape's precision is notably lower than Optiflow Front. This outcome was anticipated, as the tubes on the back of the head make it difficult to wrap the tape closely around the head. Remarkably, the PreemieScanner HC Optiflow Back measurements show no significant reduction in precision or accuracy compared to PreemieScanner HC measurements with Optiflow Front. This could be attributed to the Poisson mesh reconstruction in the calculation script, which smooths out extending artefacts, along with the script's definition of the largest section above the ears for HC measurement.

In our method we compared both PreemieScanner and measuring tape to the ground truth. A direct comparison of PreemieScanner to measuring tape, as new technique vs. gold clinical standard, is available in Supplementary Materials Data Sheet 2—Direct comparison of Measuring Tape to PreemieScanner.

### 5.5 Body length

BL measurements had the lowest accuracy and precision, with only 40% falling within precision limits. For all dolls, most measurements were lower than the GT. Suspected cause for low BL accuracy and precision is lacking 3D data, especially in the crotch and feet regions, which can be attributed to the camera positions and their resulting field of view. Additionally, because the feet rest on a soft blanket or matrass, the 3D image tends to blend the feet and the surface, making it hard to distinguish the end of the heel from the blanket. Given the constraints of measuring a curled-up infant lying in the incubator through a transparent cover, achieving an absolute precision limit of +/− 4 mm—relative to a BL of approximately 300 mm—is technically challenging. A systematic device related error may may also have contributed to the inaccuracy, although our technical verification with phantom objects demonstrated high accuracy within 1 mm. A sixth camera, pointed at the crotch, positioned on the angled part of the transparent incubator could add more 3D data, especially of the crotch, knee and heel. Future studies should investigate this. However, adding an extra camera, at that position, would make the device a lot bigger. And compactness and light weight is a critical success factor for practical implementation into standard NICU care.

Neonatologists involved in this study argued that, despite only 40% of BL measurements meeting our precision criteria, the PreemieScanner can already be regarded a preferable technique to the current caliper technique due to its stress-free measurement process. We did not evaluate the caliper technique in our study, which is a limitation. The reason for this omission was using a caliper requires straightening the leg—which could not be realistically simulated with the dolls.

### 5.6 Comparing measurements against ground truth values

Using dolls instead of real patients allowed us to compare measured values against accurately defined GT values, which we consider a strength of this study. Obtaining accurate GT values for real ELWB infants within ethical standards is nearly impossible. For this reason, most studies assessing 3D body size measuring techniques with real patients compare a novel technique with an existing “gold standard” technique (46). However, comparing a novel technique solely to an older one without accurate GT values is debatable—good correlation of the new technique with the older one does not necessarily indicate how close the measurements are to true GT values, of either the new or old technique. In other words, two very inaccurate devices could perfectly correlate with each other. Therefore, our method focused on direct comparison of measurement values relative to the true ground truth values, as this would give the most objective assessment of accuracy and precision. To comply with the approach of comparing a new device to the old, we add the direct comparison of HC measurements done with the standard measuring tape, and the PreemieScanner as a supplement.

Although we could accurately measure the dolls' GT values using an advanced medical scanner, achieving 100% accuracy was impossible. The clinical 3dMD head system scanner we used had a stated accuracy of 0.2 mm, according the manufacturer. Additionally, there was some interrater variability in placing landmarks for BL and HC/CrV measurements. To minimize this, we averaged the (x, y, z) coordinates from 12 sets of landmarks: four raters, each providing three sets.

### 5.7 Integrating scan sessions into routine care

Integrating stress-free, “all-in-one-go” Bl, HC, and CrV measurements into routine care for ELBW infants in incubators is a notable advantage of the PreemieScanner. During the scan sessions, most nurses indicated that the PreemieScanner was expected to be less stressful for infants compared to the HC measuring tape and significantly better than the BL measuring caliper.

### 5.8 CrV as longitudinal preterm growth parameter

A key clinical advantage of the PreemieScanner's measuring technique is the potential to use CrV as a longitudinal growth parameter for ELBW infants. The clinical value of CrV as a growth metric is comprehensively discussed in the study validating the MONITOR3D scanner (30).

### 5.9 Preemiescanner workload and costs

For successful implementation of a new technique such as the PreemieScanner, besides clinical reliable measurements, workload and device costs compared to currently used devices should be regarded. In our study the 3D postprocessing was done by researchers, who needed about 10 min for a merge and calculation session for one doll. Postprocessing preferably should be done by one person only, by nurses specially trained and intercalibrated for the task. A training session to learn to use the scanner could be done in 3–4 h.

Regarding hardware costs, The prototype used for this validation study has a bill of materials of roughly 5,000 euro. However, this does not include working hours for hand building the prototype, developing the software, and other development costs. If a commercial party would elaborate the PreemieScanner into a commercially available medical device, complying with EU CE MDR/FDA certification, the costs might increase.

### 5.10 Future studies

Future studies should aim to validate the PreemieScanner with real NICU patients. Such studies should focus on collecting longitudinal growth data, particularly CrV, from admission to the end of the NICU stay for a cohort of patients. The study design should prioritize patient safety, specifically examining how to plan and execute the three necessary head position changes within routine nursing shifts without disturbing the infant. The study should include adequate nurses' training for performing the scan session, placing landmarks on the 3D image, and 3D postprocessing. To make the device more suitable for such studies, further optimization of its weight, materials, and compactness is recommended. Considerations should include improving the ability to clean the device.

## 6 Conclusions

At the time of writing, the PreemieScanner is the world's first 3D technique capable of measuring BL, HC, and CrV in even the smallest ELBW preterm infants confined to incubators. Scan sessions can be seamlessly integrated into routine nursing care without causing additional disturbances to the infant. The HC measurements demonstrated high accuracy and precision, outperforming the current measuring tape technique in both aspects, with minimal disturbance to the infant. Its usability for CrV—with 93% of measurements falling within precision limits—is promising, especially considering that this technique could serve as the foundation for establishing longitudinal CrV growth reference charts for ELBW infants. This advancement has the potential to significantly enhance growth monitoring for preterm infants worldwide.

## Data Availability

The datasets presented in this study can be found in online repositories. The names of the repository/repositories and accession number(s) can be found below: https://doi.org/10.34894/OMPYOQ.

## References

[1] Ordóñez-Díaz MD Pérez-Navero JL Flores-Rojas K Olza-Meneses J Muñoz-Villanueva MC Aguilera-García CM Prematurity with extrauterine growth restriction increases the risk of higher levels of glucose, low-grade of inflammation and hypertension in prepubertal children. Front Pediatr. (2020) 8:180. 10.3389/fped.2020.00180 32373566 PMC7186313

[2] Hu F Tang Q Wang Y Wu J Ruan H Lu L Analysis of nutrition support in very low-birth-weight infants with extrauterine growth restriction. Nutr Clin Pract. (2019) 34(3):436–43. 10.1002/ncp.10210 30421458 PMC7379204

[3] Christmann V Roeleveld N Visser R Janssen AJ Reuser JJ van Goudoever JB The early postnatal nutritional intake of preterm infants affected neurodevelopmental outcomes differently in boys and girls at 24 months. Acta Paediatr. (2016) 106(2):242–9. 10.1111/apa.13669 27862266 PMC5248638

[4] Ong KK Kennedy K Castañeda-Gutiérrez E Forsyth S Godfrey KM Koletzko B Postnatal growth in preterm infants and later health outcomes: a systematic review. Acta Paediatr. (2015) 104(10):974–86. 10.1111/apa.13128 26179961 PMC5054880

[5] Ehrenkranz RA Dusick AM Vohr BR Wright LL Wrage LA Poole WK. Growth in the neonatal intensive care unit influences neurodevelopmental and growth outcomes of extremely low birth weight infants. Pediatrics. (2006) 117(4):1253–61. 10.1542/peds.2005-1368 16585322

[6] Foote JM Hanrahan K Mulder PJ Nielsen AK Perkhounkova Y Hein M Growth measurement practices from a national survey of neonatal nurses. J Pediatr Nurs. (2020) 52:10–7. 10.1016/j.pedn.2020.02.001 32062375

[7] Villar J Giuliani F Bhutta ZA Bertino E Ohuma EO Ismail LC Postnatal growth standards for preterm infants: the preterm postnatal follow-up study of the INTERGROWTH-21(st) project. Lancet Glob Health. (2015) 3(11):e681–91. 10.1016/S2214-109X(15)00163-1 26475015

[8] Villar J Altman DG Purwar M Noble JA Knight HE Ruyan P The objectives, design and implementation of the INTERGROWTH-21st project. BJOG. (2013) 120(Suppl 2):9–26. v. 10.1111/1471-0528.12047 23678873

[9] Pereira-Da-Silva L Bergmans KIM van Kerkhoven LAS Leal F Virella D Videira-Amaral JM. Reducing discomfort while measuring crown-heel length in neonates. Acta Paediatrica. (2006) 95(6):742–6. 10.1111/j.1651-2227.2006.tb02325.x 16754558

[10] Ranger M Grunau RE. Early repetitive pain in preterm infants in relation to the developing brain. Pain Manag. (2014) 4(1):57–67. 10.2217/pmt.13.61 24641344 PMC3975052

[11] Brummelte S Grunau RE Chau V Poskitt KJ Brant R Vinall J Procedural pain and brain development in premature newborns. Ann Neurol. (2012) 71(3):385–96. 10.1002/ana.22267 22374882 PMC3760843

[12] Ranger M Chau CM Garg A Woodward TS Beg MF Bjornson B Neonatal pain-related stress predicts cortical thickness at age 7 years in children born very preterm. PLoS One. (2013) 8(10):e76702. 10.1371/journal.pone.0076702 24204657 PMC3800011

[13] Grunau RE. Neonatal pain in very preterm infants: long-term effects on brain, neurodevelopment and pain reactivity. Rambam Maimonides Med J. (2013) 4(4):e0025. 10.5041/rmmj.10132 24228168 PMC3820298

[14] Doesburg SM Chau CM Cheung TPL Moiseev A Ribary U Herdman AT Neonatal pain-related stress, functional cortical activity and visual-perceptual abilities in school-age children born at extremely low gestational age. Pain. (2013) 154(10):1946–52. 10.1016/j.pain.2013.04.009 23711638 PMC3778166

[15] Grunau RE Whitfield MF Petrie-Thomas J Synnes AR Cepeda IL Keidar A Neonatal pain, parenting stress and interaction, in relation to cognitive and motor development at 8 and 18 months in preterm infants. Pain. (2009) 143(1-2):138–46. 10.1016/j.pain.2009.02.014 19307058 PMC2836793

[16] Ranger M Zwicker JG Chau CM Park MT Chakravarthy MM Poskitt K Neonatal pain and infection relate to smaller cerebellum in very preterm children at school age. J Pediatr. (2015) 167(2):292–8.e1. 10.1016/j.jpeds.2015.04.055 25987534

[17] Moody C Callahan TJ Aldrich H Gance-Cleveland B Sables-Baus S. Early initiation of newborn individualized developmental care and assessment program (NIDCAP) reduces length of stay: a quality improvement project. J Pediatr Nurs. (2017) 32:59–63. 10.1016/j.pedn.2016.11.001 27923536

[18] Als H Duffy FH McAnulty G Butler SC Lightbody L Kosta S NIDCAP Improves brain function and structure in preterm infants with severe intrauterine growth restriction. J Perinatol. (2012) 32(10):797–803. 10.1038/jp.2011.201 22301525 PMC3461405

[19] VandenBerg KA. Individualized developmental care for high risk newborns in the NICU: a practice guideline. Early Hum Dev. (2007) 83(7):433–42. 10.1016/j.earlhumdev.2007.03.008 17467932

[20] Burkhardt W Schneider D Hahn G Konstantelos D Maas HG Rüdiger M. Non-invasive estimation of brain-volume in infants. Early Hum Dev. (2019) 132:52–7. 10.1016/j.earlhumdev.2019.03.020 30986647

[21] Ifflaender S Rüdiger M Koch A Burkhardt W. Three-dimensional digital capture of head size in neonates—a method evaluation. PLoS One. (2013) 8(4):e61274. 10.1371/journal.pone.0061274 23580107 PMC3620274

[22] Martini M Klausing A Lüchters G Heim N Messing-Jünger M. Head circumference—a useful single parameter for skull volume development in cranial growth analysis? Head Face Med. (2018) 14(1):3. 10.1186/s13005-017-0159-8 29321071 PMC5764008

[23] Meyer-Marcotty P Kunz F Schweitzer T Wachter B Bohm H Wasmuth N Cranial growth in infants-A longitudinal three-dimensional analysis of the first months of life. J Craniomaxillofac Surg. (2018) 46(6):987–93. 10.1016/j.jcms.2018.04.009 29709329

[24] Santander P Quast A Hubbert J Horn S Meyer-Marcotty P Kuster H Three-dimensional head shape acquisition in preterm infants—translating an orthodontic imaging procedure into neonatal care. Early Hum Dev. (2019) 140:104908. 10.1016/j.earlhumdev.2019.104908 31670175

[25] Young A Andrews ET Ashton JJ Pearson F Beattie RM Johnson MJ. Generating longitudinal growth charts from preterm infants fed to current recommendations. Arch Dis Child Fetal Neonatal Ed. (2020) 105(6):646. 10.1136/archdischild-2019-318404 32451355

[26] RCPCH. Uk-who Growth Charts-neonatal and Infant Close Monitoring (nicm) London. UK: Royal College of Paediatrics and Child Health (2019).

[27] Villar J Giuliani F Fenton TR Ohuma EO Ismail LC Kennedy SH. INTERGROWTH-21st very preterm size at birth reference charts. Lancet. (2016) 387(10021):844–5. 10.1016/S0140-6736(16)00384-6 26898853

[28] Vermeulen MJ Burkhardt W Fritze A Roelants J Mense L Willemsen S Reference charts for neonatal cranial volume based on 3D laser scanning to monitor head growth. Front Pediatr. (2021) 9:654112. 10.3389/fped.2021.654112 34123964 PMC8192695

[29] Dieks JK Jünemann L Hensel KO Bergmann C Schmidt S Quast A Stereophotogrammetry can feasibly assess ‘physiological’ longitudinal three-dimensional head development of very preterm infants from birth to term. Sci Rep. (2022) 12(1):8940. 10.1038/s41598-022-12887-x 35624305 PMC9136805

[30] Van Gils RHJ Helder OK Kornelisse RF Reiss I Dankelman J. 3D Scanner measuring preterm infants’ head circumference and cranial volume: validation in a simulated care setting. Front Med Eng. (2024) 2. 10.3389/fmede.2024.146379339925363

[31] Kubik A Welton J Holmes L Struwe L Gonzales K. The PLAY bundle for head deformities: a NICU quality improvement project. J Neonatal Nurs. (2023) 30:263–70. 10.1016/j.jnn.2023.10.006

[32] De Koning M. Protocol: Wisselligging bij neonaat met bewegingsarmoede. In: Rotterdam EMUMC, editor. Rotterdam: Quality Management System, Erasmus MC University Medical Centre Rotterdam. (2020).

[33] van Gils RHJ Helder OK Kornelisse RF Singowikromo TMS Reiss I Dankelman J. 3D PreemieScanner Dataset. V1 ed: DataverseNL. (2025). 10.3389/fmedt.2025.1607538 PMC12354455 40823624

[34] ISO. ISO-5725-1 Accuracy of Measurement Methods and Results—part 1: General Principles and Definitions. 2nd ed. Geneva, Switzerland: International Organization for Standardization (2023).

[35] Mokkink LB Terwee CB Patrick DL Alonso J Stratford PW Knol DL The COSMIN study reached international consensus on taxonomy, terminology, and definitions of measurement properties for health-related patient-reported outcomes. J Clin Epidemiol. (2010) 63(7):737–45. 10.1016/j.jclinepi.2010.02.006 20494804

[36] COSMIN. COSMIN-COnsensus-based Standards for the selection of health Measurement INstruments: Amsterdam Public Health, AMC, VUmc, VU, UvA (founded). (2005). Available online at: https://www.cosmin.nl/ (Accessed June 25, 2025).

[37] Health UDo. UK-WHO growth charts—neonatal and infant close monitoring. (2009).

[38] Greer FR Olsen IE. How fast should the preterm infant grow? Curr Pediatr Rep. (2013) 1(4):240–6. 10.1007/s40124-013-0029-1

[39] Horemuzova E Söder O Hagenäs L. Growth charts for monitoring postnatal growth at NICU of extreme preterm-born infants. Acta Paediatr. (2012) 101(3):292–9. 10.1111/j.1651-2227.2011.02510.x 22040370

[40] Riddle WR DonLevy SC Lafleur BJ Rosenbloom ST Shenai JP. Equations describing percentiles for birth weight, head circumference, and length of preterm infants. J Perinatol. (2006) 26(9):556–61. 10.1038/sj.jp.7211572 16885988

[41] Altigani M Murphy JF Newcombe RG Gray OP. Catch up growth in preterm infants. Acta Paediatr Scand Suppl. (1989) 357:3–19. 10.1111/j.1651-2227.1989.tb11270.x 2487016

[42] Babson SG. Growth of low-birth-weight infants. J Pediatr. (1970) 77(1):11–8. 10.1016/S0022-3476(70)80039-7 5464382

[43] Andrews ET Ashton JJ Pearson F Beattie RM Johnson MJ. Handheld 3D scanning as a minimally invasive measuring technique for neonatal anthropometry. Clin Nutr ESPEN. (2019) 33:279–82. 10.1016/j.clnesp.2019.06.012 31451267

[44] Charder-Medical. USER MANUAL HM80D Digital Infant Stadiometer. Taichung, Taiwan: Charder Electronic Co., Ltd (2024). p. 13.

[45] Seca. Seca 212-Measuring tape for Head Circumference of Babies and Toddlers: Seca Gmbh & co. kg. Hamburg: Hamburg PHG Sönke Vogel GmbH (2025). [seca 212Measuring tape for head circumference of babies and toddlers]. Available online at: https://de.secashop.com/produkte/messsysteme-p%C3%A4diatrie/seca-212/2121717009

[46] Van Gils RHJ Wauben LSGL Helder OK. Body size measuring techniques enabling stress-free growth monitoring of extreme preterm infants inside incubators: a systematic review. PLoS One. (2022) 17(4):e0267285. 10.1371/journal.pone.0267285 35452486 PMC9033282

