# Supplementary material for: Effectiveness of the novel 3D PreemieScanner for preterm infants’ growth monitoring confirmed in a simulated setting
Source: Front Med Technol. 2025 Aug 1;7:1607538. doi: 10.3389/fmedt.2025.1607538 (PMC12354455; doi:10.3389/fmedt.2025.1607538)
Supplement: Supplementary file 1 [file Datasheet1.pdf]

README

3D PreemieScanner Dataset. Available at Dataverse.nl repository: 3D PreemieScanner - Research centre Innovations in Care

Abbreviations:

HC, Head Circumference

CrV, Cranial Volume

BL, Body Length

PrSc, PreemieScanner

GT, Ground Truth = true values, to act as reference values

NICU, Neonatal Intensive Care Unit

Aim of dataset:

The dataset was generated to evaluate a novel 3D scanner, the PreemieScanner (PrSc). This scanner was specially developed for **measuring growth of preterm infants lying in neonatal incubators**. The PrSc can make scans from outside the incubator, through the transparent cover. The study evaluated the clinical usability of **body length (BL), head circumference (HC) and cranial volume (CrV)** measurements using the scanner in a simulated NICU setting.

In this study we use following clinical definitions for body length (BL), head circumference (HC) and cranial volume (CrV):

Body Length (in cm) is the sum of three segments: crown to crotch, crotch to (inside of) knee, knee to heel.

Cranial Volume (in cm^3 , mL) is the volume above a virtual plane through three anatomical points of the head: tragus left, tragus, right, nasion. This is roughly a plane through both ears and nose.

Head Circumference (in cm) is the largest circumference parallel to the tragi-nasion plane, roughly located just above the eyebrows.

The dataset contains five main folders (zipped):

Folder	Description
Ground_Truth_3dMD	**Contains: all data to calculate Ground Truth (GT) values.** GT values are the true BL,HC and CrV values of the dolls, defined using an accurate, commercially available 3D scanner (3dMD head system). The GT values were used in the study to determine the measurement error (ME), the deviation of a measured value from the ground truth (GT) value.
Nurses_Scan_Sessions_1<br>Nurses_Scan_Sessions_2<br>Nurses_Scan_Sessions_3	**Contains: all 3D data and data to calculate BL,HC and CrV values of the scan sessions performed by nurses.** Nurses performed **scan sessions with the PrSc in a simulated NICU setting**. Each nurse scanned one of the three prepared dolls, lying in a neonatal incubator (dolls IDs: Ellen, Sophie, To). On a laptop, **nurses marked anatomical points on the 3D images** of the doll. These anatomical points were needed to calculate BL, HC and CrV from the 3D images.<br>(Nurses_Scan_Sessions data were split into 3 folders due to limited data size upload)<br><br>Subfolder : Nurses_Scan_Sessions _1/ poisson reconstruction files: This folder contains all **3D 'poisson surface reconstruction files'**, copied out of the VPK01…VPK35 folders. The poisson surface reconstruction file was automatically generated by the PrSC calculation script to facilitate calculation, and used to assess if the 3D poisson reconstruction was suitable for a valid calculation of HC and CrV
Technical_Verification	Researchers made scans with the PrSc of the three dolls, lying in an incubator, in a controlled setting. Aim was to **verify technical instrument accuracy and precision** relative to the GT values, to assess possible systematic errors.

Folder: Ground_Truth_3dMD:

Ground Truth (GT) values were derived from accurate 3D scans from the dolls, using a commercially available medical scanner with known high accuracy and precision (3dMD head system, purchased in 2020, 3dMD, Atlanta, USA, with a manufacturer-claimed shape accuracy of 0.2 mm). The anatomical 3D points, needed for the calculation script to calculate the GT values, were constructed from multiple pointsets, marked by four different raters (researchers). For each doll, each of the four raters marked three sets of anatomical 3D points, for both BL

and HC/CrV, with each 3D point represented by its x,y,z, coordinate. From these sets, the average x, y, z coordinates were calculated and stored as **GT pointset**, to be used to calculate GT values for BL, HC and CrV for each doll.

The different file types in the folder are described in the table below, by taking typical examples. Typical identifiers in file names are explained.

**Path: Ground_Truth_3dMD/**	**Contains/ description**	**Identifiers in file/folder name**
GROUND TRUTH FILES	The XYZ coordinates of anatomical points to calculate GT values, calculated as average from the points marked by four researchers (A,B,C,D)	
GROUND TRUTH FILES/ Script for Ground Truth PreemieScanner	Python scripts to calculate the average XYX coordinates of the multiple anatomical pointsets, marked by four researchers (A, B, C, D) on 3D scans, captured with a 3dMD head system 3D scanner, to define ground truth values.	
GROUND TRUTH FILES/ 3dmd_Ellen_A/	Folder with 3D data generated with the 3dMD scanner & XYZ coordinates of anatomical points, marked by researcher	3dmd= type of scanner used (3dMD head system, purchased in 2020, 3dMD, Atlanta, USA);<br>Ellen= ID of doll (Ellen, Sophie, To);<br>A= ID of researcher (A,B,C,D)
20240523_Ellen_000021.ply	Raw 3dMD scan	
20240523_Ellen_000021_reconstruction.ply	Poisson reconstruction file, generated by calculation script	
20240523_Ellen_000021.ply_body_measurement_03-06-2024_12-33-23.json	GT pointset file for BL GT, with the averaged XYZ coordinates of anatomical points. Including calculated GT values of length per segment and BL	20240523= scan date<br>Ellen_000021= ID of doll (Ellen, Sophie, To)_scanID;<br>body_measurement_03-06-2024_12-33-23= BL calculation_timestamp

20240523_Ellen_000021.ply_head_measurement_03-06-2024_12-34-21.json	GT pointset file for HC/CrV GT, with the averaged XYZ coordinates of anatomical points marked on head. Including calculated GT values for HC and CrV	20240523= scan date<br>Ellen_000021= ID of doll (Ellen, Sophie, To)_scanID;<br>head_measurement_03-06-2024_12-34-21= HC/CrV calculation_timestamp

Folders: Nurses_Scan_Sessions _1, Nurses_Scan_Sessions_2, Nurses_Scan_Sessions_3

Collection of the PreemieScanner 3D data of the dolls, needed anatomical landmarks on the 3D images, and the actual BL, HC and CrV derived from the 3D data, consisted of five steps:

1. Capture of raw 3D images:

In the scan sessions, each nurse (VPK01…VPK35) conducted three captures of one of the dolls (Ellen, Sophie or To). To simulate the NICU setting, dolls were lying in a neonatal incubator at a NICU bedspace. Dolls were prepared with a breathing system, feeding tube, ECG electrodes on the chest and blood saturation sensor on a feet. The position of the doll's head was changed in between captures, with the head facing front, right and left. This generated the **raw 3D capture as 3D mesh** in .ply format, with prefix mid, left, right.

2. Marking of anatomical landmarks on 3D image:

On the front raw capture (**mid_xxx_xxx.ply**) anatomical points (or: 'landmarks') were selected and marked on the 3D image of the doll, shown on a laptop screen. Anatomical points for body length: crown-crotch-knee-heel. Anatomical points for HC/CrV: nasion-left tragus-right tragus for HC/CrV. Marking was by done by double clicking on the 3D image, shown in colour on the laptop screen, using a computer mouse. Users could rotate and zoom in/out the 3D image to find the correct location of anatomical points on the body and head. Marking of landmarks was done three times in a row, resulting in three sets of paired 3D points for both BL and HC/CrV. For each pointset a separate .json file was generated. For the body length **mid_xxx_body_measurement_xxx.json**. For the head **mid_xxx_point_selection_xxx.json**),

3. Calculation of BL.

For BL, the script calculates the 3D distance between points placed on the mid_xxx_xxx.ply 3D image. Distance between 3D points is calculate and shown instantly on the laptop screen, as soon as two landmarks are marked op the 3D image. A pair of two consecutively

marked points form a segment, e.g. crown-crotch. The screen shows the distance (cm) between each pair of two points (called segments), and the total sum of all segments, representing the total body length. Each BL measurement was saved as **.json data file** (**mid_xxx_body_measurement_xxx.json**). Each json file contains a set of marked anatomical points as 3D (x,y,z) coordinates ("points":[...]), in the order of when marked, the calculated BL as the sum of segments in xx.xx cm ("body_length": "32.73").

4. Merging of three raw scans (mid, left, right) to one complete, merged head:

For each scan session, a 3D image of the complete head was constructed by merging the three raw captures (mid, left, right) into one. The result was saved as **xxx_xxx_merged_x.ply**. The merging was done at a later moment – not during the scan session, by four researchers, with IDs A,B,C and D.

The merging procedure involved a combination of manual and automatic algorithms, all part of the Meshlab software application software (MeshLab 2023.12, Visual Computing Lab, ISTI-CNR, Pisa, Italy). Input files are the three raw captures (mid, left, right). The raw captures are generated as 3D meshes in the .ply 3D file format. First step is 'cleaning': the head is separated from the body and other unwanted artefacts (such as the blanket, matrass, infant's shoulders) by manually selecting and deleting 3D points. Second step is alignment of the cleaned meshes relative to each other, matching overlapping areas with each other. Alignment is done with the Meshlab 'align' function, using 'point based glueing'. With point based glueing, four 'matching pair of points' are manually selected on the overlapping areas of both meshes. Using the four pair of points, the algorithm roughly aligns meshes to each other in 3D space. After rough alignment, an 'iterative closest point' (ICP) algorithm automatically 'fine tunes' alignment by iteratively calculating the smallest average distances between 3D points of both meshes. After aligning two meshes, a third mesh is added and aligned with the set, etcetera, until all nine captures form one head, that is exported as one unified mesh (**xxx_xxx_merged_x.ply).**

5. Calculation of HC and CrV:

To calculate HC and CrV, the merged head is imported in the PrSc HC/CrV calculation script. Subsequently, the saved mid_xxx_point_selection_xxx.json file, with the XYX coordinates of landmarks marked by the nurse, is imported. After importing the merged 3D head (**xxx_xxx_merged_x.ply**) with the marked points, the script can calculate HC and CrV. To facilitate calculation, the script first reconstructs the merged mesh using a **poisson surface reconstruction filter.** This results in a poisson reconstruction of the mesh **(merged_reconstruction.ply).**

CrV is the volume above the tragi-nasion plane. To calculate CrV the script constructs this plane in the poisson reconstruction mesh, through the three marked anatomical points, imported from the **mid_xxx_point_selection_xxx.json** file. Then, for CrV, the script calculates the volume of the 3D mesh located above the plane.

HC is the largest circumference parallel to the tragi-nasion plane, roughly located just above the eyebrows. For HC, the script slices the mesh above to plane, parallel to the plane, and determines the slice with largest surface area, representing the largest head circumference. Slices are calculates with 1 mm intervals. Slicing starts from an specific offset above the plane, to start slicing above the ears. Slicing should start above the ears to prevent that the extra volume near the ears will lead to define the wrong section with largest circumference.

The results are stored in a .json file (**xxx_xxx_merged_xxx_head_measurement_xxx.json**) with CrV in cm^3 ("cranial_volume": "xxx.xx") and HC in cm ("head_circumference": "xx.xx") and ("points":[...]), in the order of when marked.

To validly derive HC or CrV, the merged head should be assessed as valid. This assessment was done by visual examination of the **poisson surface reconstruction (merged_reconstruction.ply)**, that was automatically generated by the HC/CrV calculation script. Poisson reconstructions with large deformations, were regarded as invalid.

**Path: Nurses_Scan_Sessions _1, Nurses_Scan_Sessions_2, Nurses_Scan_Sessions_3**	**Contains/ description**	**Identifiers in file/folder name**
Nurses_Scan_Sessions _1/ **poisson reconstruction files**	This folder contains all 3D ‘poisson surface reconstruction files’, copied out of the VPK01...VPK35 folders. The poisson surface reconstruction file was automatically generated by the PrSC calculation script to facilitate calculation, and used to assess if the 3D poisson reconstruction was suitable for a valid calculation of HC and CrV	

VPK01….VPK35	Each VPK folder contains 3D data of dolls, as captured with the PreemieScanner by one nurse, content per VLK folder is explained in detail below	VPK01= nurse ID (VPK01…VPK35)
mid_27-05-2024_15-01-53.ply	raw 3D capture #1, from a doll, made by a nurse with the PreemieScanner	mid= front side of head_timestamp
right_27-05-2024_15-05-56.ply	raw 3D capture #2,	right= right side of head_timestamp
left_27-05-2024_15-07-15.ply	raw 3D capture #3	left= left side of head_timestamp
VPK01_Ellen_merged_A.ply	Raw 3D captures merged to one head, by manual postprocessing	VPK01 = nurse ID,<br>Ellen= name of doll, (Ellen, Sophie, To)<br>merged_A= Merged by researcher #A. (A,B,C,D)
VPK01_Ellen_merged_A_reconstruction.ply	'poisson surface reconstruction', automatically generated by the PreemieScanner software, as part of the HC/CrV calculation script	VPK01 = nurse ID,<br>Ellen= name of doll,<br>merged_A= Merged by researcher #A,<br>reconstruction= poisson surface reconstruction
mid_27-05-2024_15-01-53_body_measurement_27-05-2024_15-03-37.json	Output file of the BL calculation script: a nurse marks four anatomical points on the body: crown, crotch, knee, heel. The script instantly calculates 3D distances between points. Always done on mid raw capture. Done three times on a row. Contains: XYZ_coordinates of marked anatomical points, length per segment, total length. Output file of BL	Mid= raw capture_timestamp,<br>body_measurement= BL calculation_timestamp.
mid_27-05-2024_15-01-53_point_selection_27-05-2024_15-04-41.json	Output file of 3D point selection of the head: a nurse marks three anatomical points on the head: tragus left, tragus right, nasion. Always	Mid= raw capture_timestamp,<br>point_selection= head anatomical points_timestamp.

	done on mid raw capture. Done three times on a row. File contains XYZ coordinates of marked points	
VPK01_Ellen_merged_A.ply_head_measurement_29-05-2024_14-38-51.json	Output file of HC/CrV calculation script. To generate this file, the point_selection file, containing the 3D coordinates of points marked on the head buy the nurse: tragus left, tragus right, nasion, is imported into the script. File contains XYZ coordinates of marked points, HC value, CrV value	VPK01 = nurse ID,<br>Ellen= name of doll,<br>merged_A= Merged by researcher #A,<br>head_measurement= HC and CrV calculation_timestamp
Poisson reconstruction files		

Folder: Technical_Verification

Aim of data in this folder was to **verify technical instrument accuracy and precision** relative to the GT values, to assess possible systematic errors. Researchers collected this data by scanning the three dolls, lying in an incubator. This was done, in a controlled setting, meaning that dolls were scanned lying in the incubator, but without breathing system, feeding tube, ECG electrodes and blood saturation sensor.

**Path: Technical_Verification/**	**Contains/ description**	**Identifiers in file/folder name**
Ellen1.....to5	3D data captured with the PreemieScanner by researchers.	
e.g. sophie5/		
left_23-05-2024_12-35-56.ply	Raw 3D capture	Left= orientatation of doll's head, left side of head captured_timestamp
sophie5_merged.ply	Merged 3D head, by manual 3D postprocessing, merging the mid, left and right raw scan to one complete head	
sophie5_merged_aligned.ply	Idem, but **coordinate system aligned** with coordinate system of 3dMD GT scans, in order	

	to use exactly the same anatomical pointsets of GT pointssets for fair comparison of BL. HC. CrV values between PrSC and 3dMD scans	
