# Supplementary material for: Effectiveness of the novel 3D PreemieScanner for preterm infants’ growth monitoring confirmed in a simulated setting
Source: Front Med Technol. 2025 Aug 1;7:1607538. doi: 10.3389/fmedt.2025.1607538 (PMC12354455; doi:10.3389/fmedt.2025.1607538)
Supplement: Supplementary file 2 [file Datasheet2.pdf]

Supplements to the PreemieScanner validation study

Table of contents	Page
Supplement 1: Accuracy and Precision	2
Supplement 2: Nurses’ inter- and intrarater variability	9
Supplement 3: Direct comparison of Measuring Tape to PreemieScanner	13

Supplement 1: Accuracy and Precision

This supplement presents additional figures and clarification to the method used to assess clinical usability. This clinical usability is defined by accuracy and precision, relative to the true, ground truth values of the objects (the dolls) measured, related to clinically allowed limits for the measurement error. These clinical allowed limits are based on the smallest weekly increase the device should detect with sufficient statistical confidence.

Principles of measurement error, accuracy and precision

Figure 1 shows a fictive plot, generated using fictional measurement values, such as those from a fictional instrument measuring head circumference (HC). The measurement values will inevitably deviate from the true values of the doll, the ground truth (GT) values. These GT values remain constant, as the doll will not grow. This deviation from the GT is known as the measurement error (ME). The MEs of observed repeated measurements will show a normal or non-normal distribution. Key elements are the clinically acceptable precision limits, which relate to the expected weekly growth increase. This is explained below.

Clinically acceptable precision limits related to expected weekly growth

For longitudinal growth monitoring, repeated measurements are taken over time, typically on a weekly basis in NICU practice. In real patients, unlike the dolls used in this study, the GT will change over time as the infant grows. Weekly measured values are plotted on growth reference charts (Figure 2), allowing for a comparison between the measured growth curve and the percentile growth trajectory curves (shown in pink). Figure 3 presents a detailed zoom-in (box A) of the growth reference chart with fictional true growth data for a hypothetical preterm infant.

Figures 4-6 present hypothetical plots demonstrating the effect of measurement error (ME) on growth trajectory tracking.

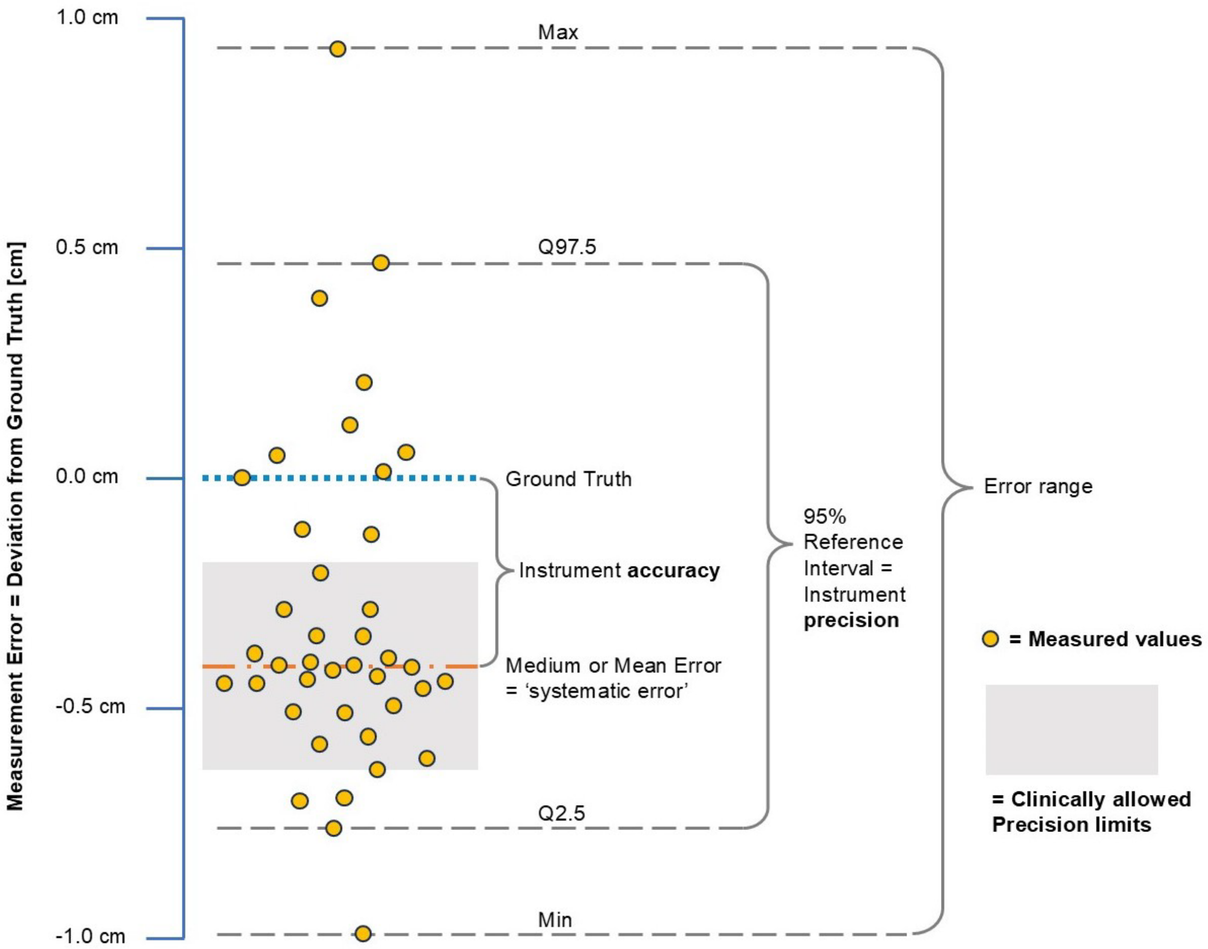

Figure 1. Measurement error, accuracy and precision. Positive MEs indicate measured values larger than the GT values, while negative MRs indicate values smaller than the GT values. Accuracy is defined by the medium of mean error (dashed dotted line), also referred to as systematic error. Precision in this study is defined by the 95% reference interval. The error range contains all ME values, ranging from the min to max ME. Clinical usability is expressed as the percentage of MEs (from the min-max range) within the clinically acceptable precision limits, as +/- margin relative to the median/mean error, visualized by the grey box.

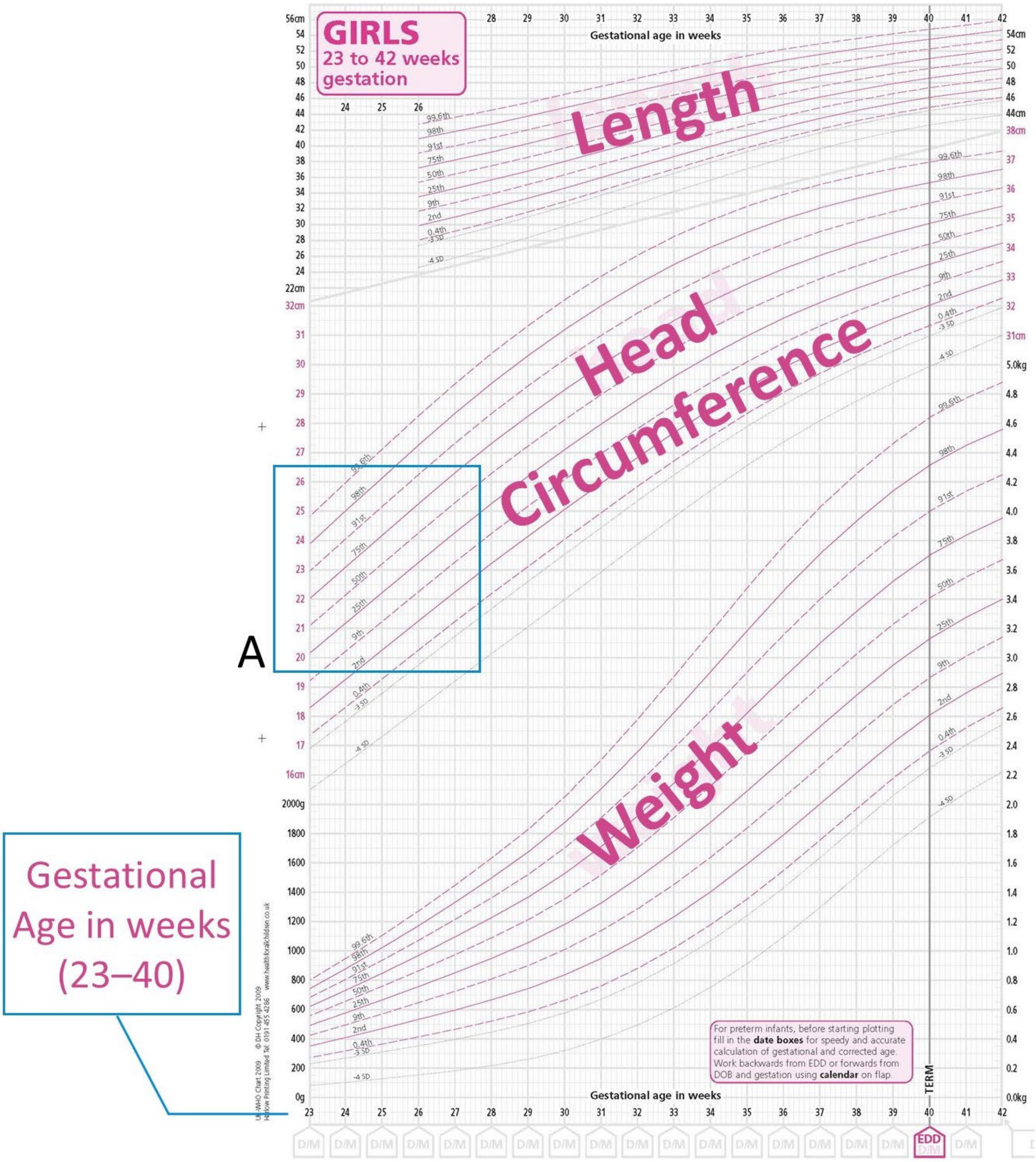

Figure 2. Example of a neonatal growth reference chart. Box A, indicated by a blue solid line, and covering the HC growth of an 23-27 GA preterm, is enlarged in the figure below, with added fictional growth data.

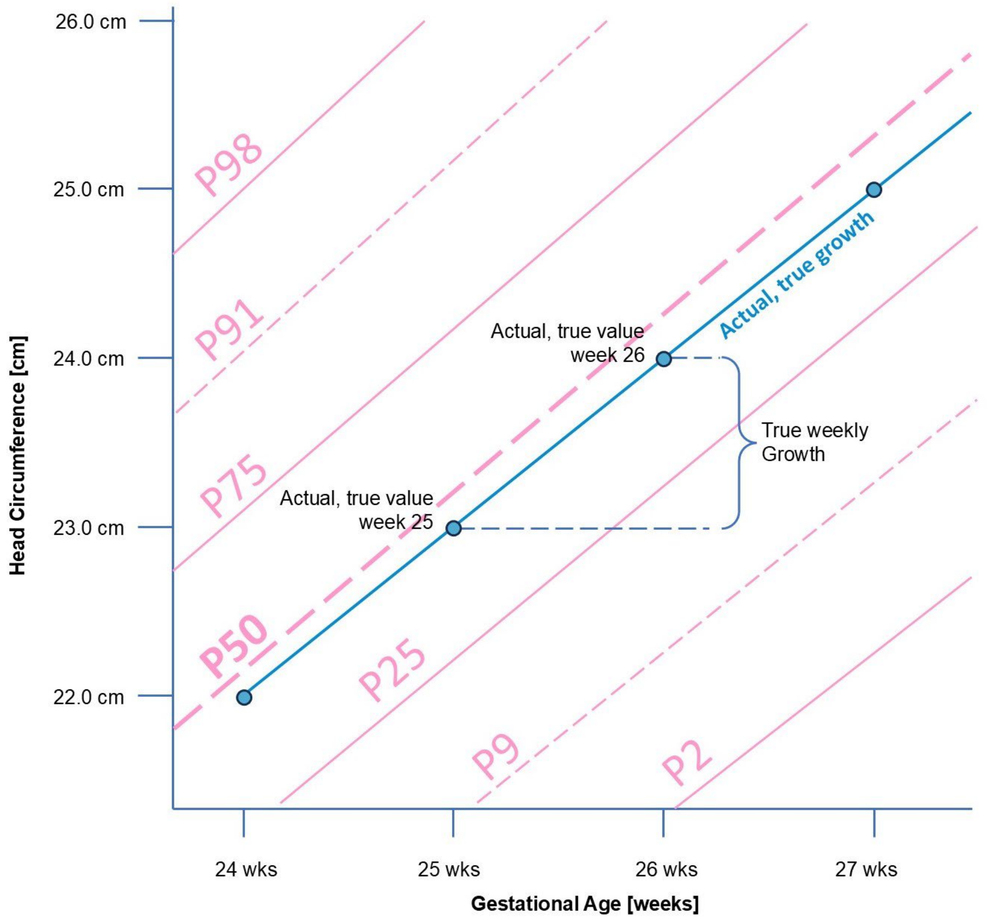

Figure 3.

Enlargement of box A in fig1, with added fictive HC true growth data of a fictive patient. Expected weekly growth, based on the growth charts, defines the neccesary instrument precision.

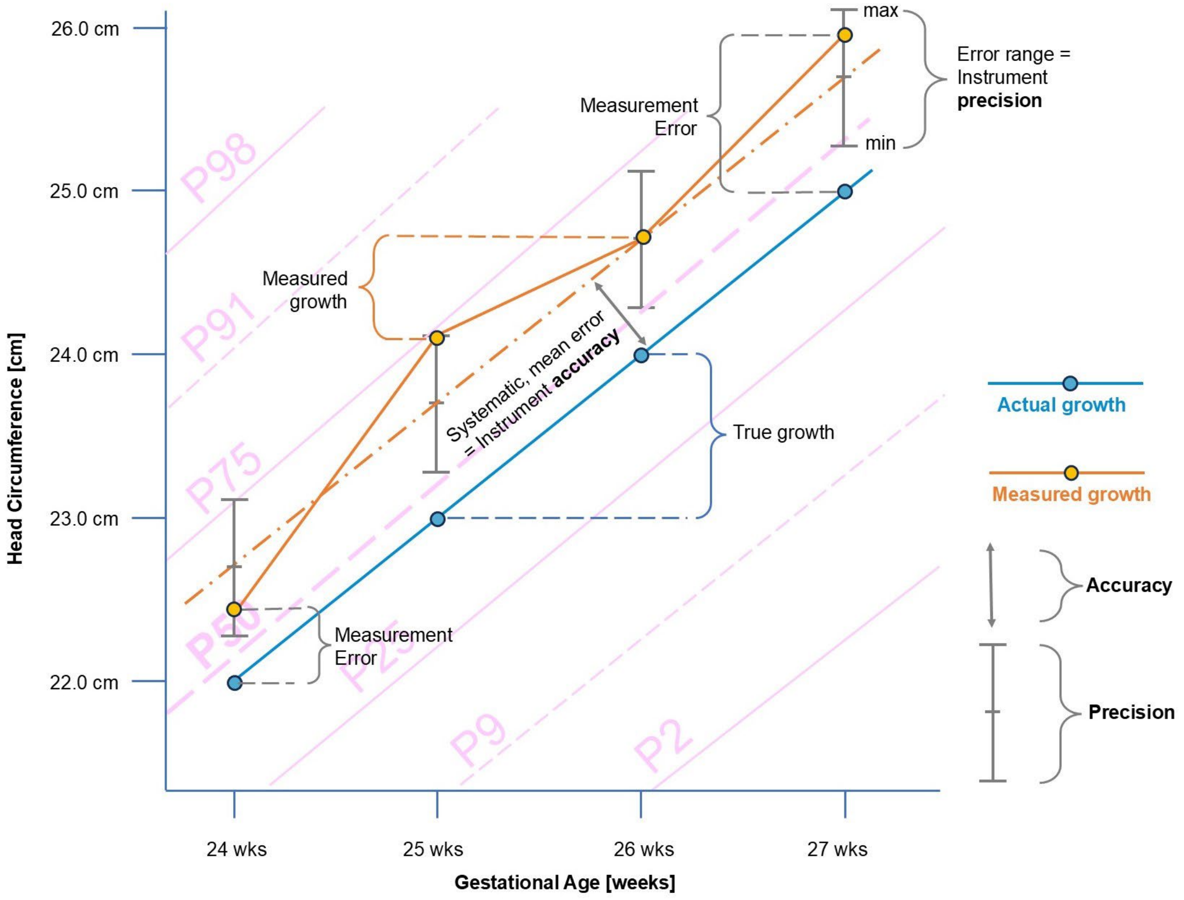

Figure 4. Fictive HC measurements plotted on the growth reference chart. The blue line represents the true growth trajectory, while the yellow line shows the measured values obtained with a hypothetical instrument. The difference between the measured and true growth represents the measurement error. Accuracy is the mean or median deviation from GT, represented by the yellow dashed-dot line. Precision is the spread of the occurring errors, represented by the error range. For longitudinal growth monitoring, instrument precision is more critical for clinical usability than accuracy. Therefore, in our setting, it is appropriate to define clinically allowed precision limits based on an error interval, with the mean or medium 'systematic' error as the midpoint. The instrument's performance can be assessed by the percentage of measurements falling within these limits.

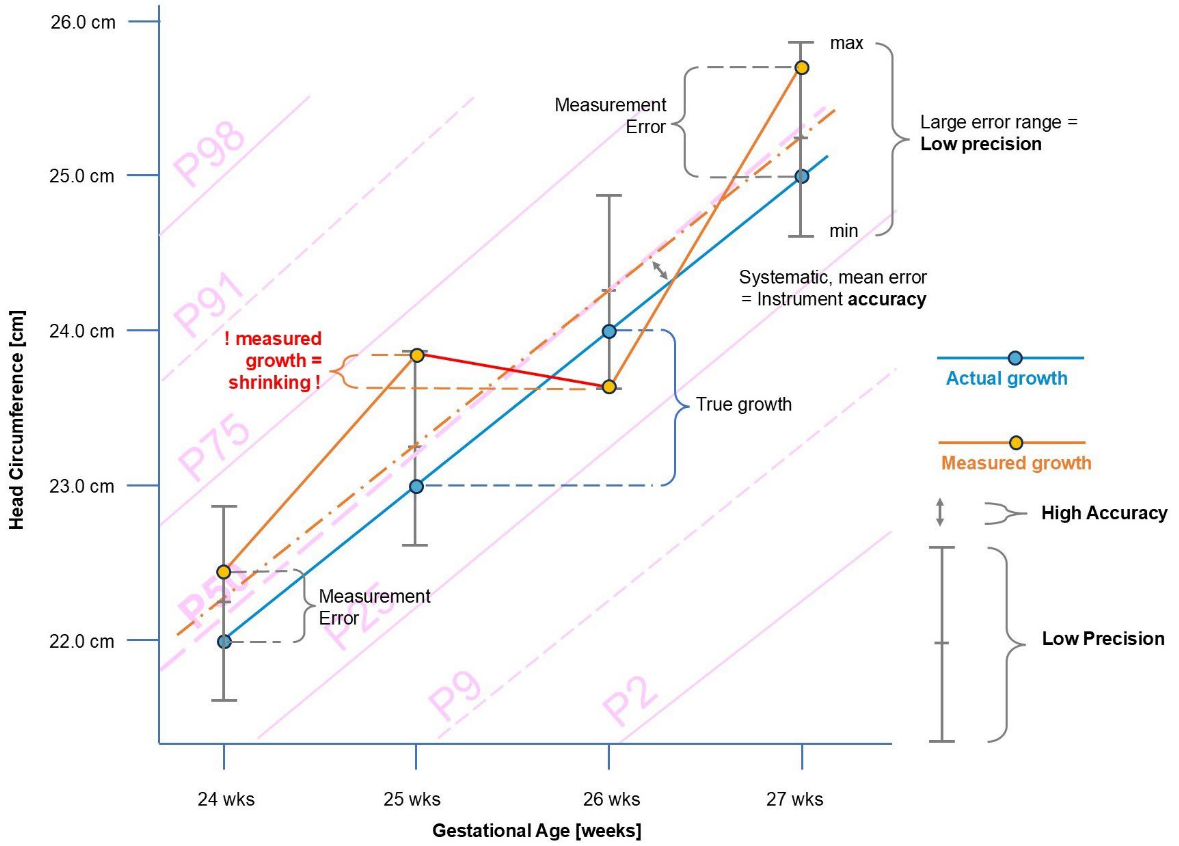

Figure 5. This plot shows an instruments with low precision, but, due how accuracy is defined, high accuracy. Because of the large error intervals, the measured growth seems very unstable. And if, as in this example, the instrument's error range exceeds the true growth, 'shrinkage' may be recorded, even though the true growth follows the percentile reference curves accurately. Therefore, ***to prevent 'shrinkage' being mistakenly measured, the instrument's allowed precision limits should at least be equal to the minimum expected growth in one week.***

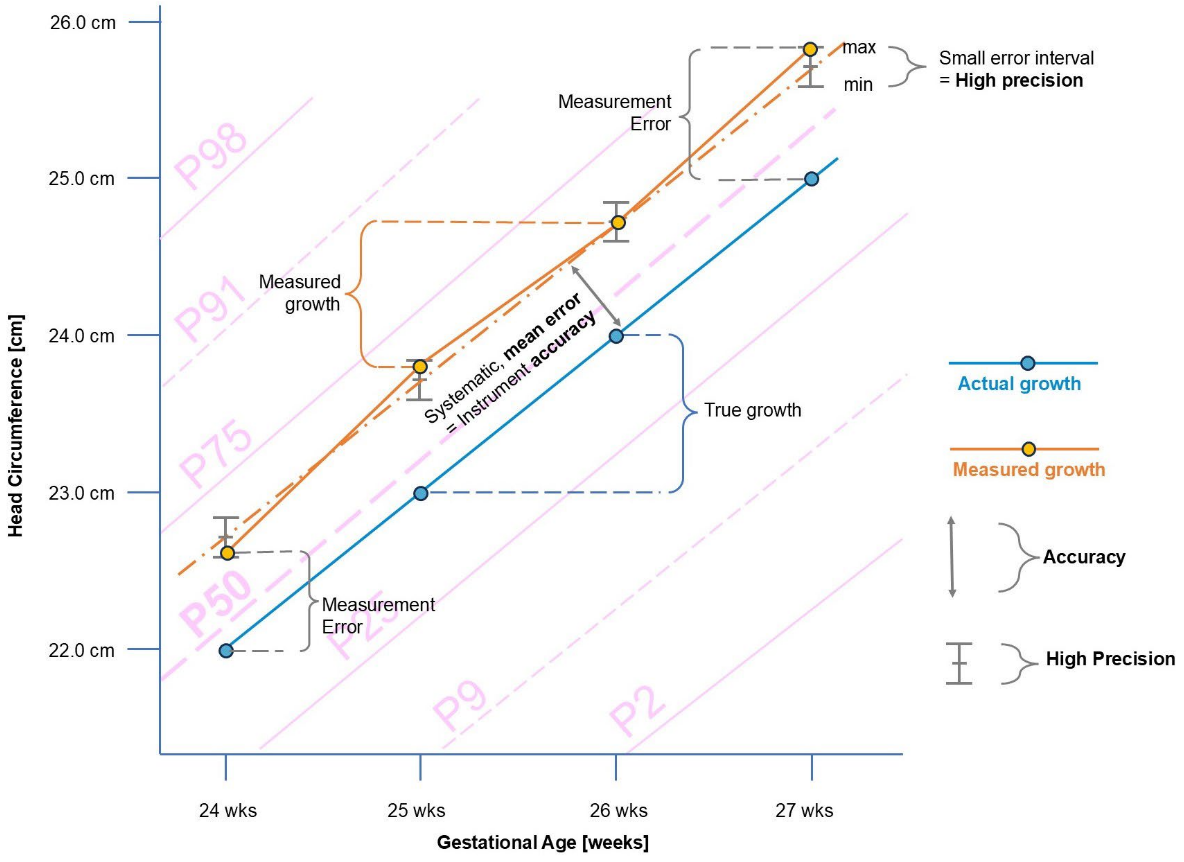

Figure 6. This plots show a hypothetical instrument with high precision but (relatively) low accuracy. The measured growth curve closely follows (one of) the reference growth curve, showing a constant, stable weekly growth. Again, for longitudinal growth monitoring high precision is preferred over high accuracy.

Supplement 2: Nurses' inter- and intrarater variability

METHOD

For the ideal measuring instrument, the rater's influence on the ME should be as little as possible. In our case, rater's influence is the manual marking of the anatomical landmarks on the 3D image, which directly influences BL, HC and CrV measurement outcomes. In other words, the agreement between of different raters, as well as consistency within one rater should be high.

Dataset

In a scan session, one individual nurse did three consecutive 'measurements' of BL, HC and CrV with the PreemieScanner, by marking three consecutive sets of anatomical landmarks on the 3D image of the laptop screen: three sets on the body for BL, and three sets on the head for HC/CrV. After that, on the same doll, the nurse performed three consecutive HC measurements using the standard measuring tape, each time wrapping the tape round the doll's head and reading off the HC on the tape. All 35 scan sessions together resulted in 35 sets of three measurements for each device: three HC with tape, three BL/HC/CrV with PrSc. However, it should be noticed that for seven of the 35 nurses the CrV measurement was missing because the 3D merged head was deemed invalid for CrV measurement.

Data analysis

We assessed consistency of measurements within one nurse by the intra class correlation (ICC) for intrarater variability/reliability, and agreement between different nurses by the ICC for interrater variability/reliability.

Statistical analysis

Intrarater ICC was calculated using a one-way random effects model, interrater ICC by a two-way random effects model.

RESULTS

Inter and intrarater variability

Table 1 shows the interrater ICC to express agreement between nurses' measurements, and intrarater ICC for consistency of the three measurements within the same nurse.

Table 1. Inter and Intrarater variability

**Optiflow method**	**Intrarater ICC[a]**			
	**Length**	**HC PrSc**	**HC tape**	**CrV**
Optiflow Front		0.97	0.79	0.84
Optiflow Back	0.82	0.98	0.78	0.60

**Optiflow method**	**Interrater ICC[b]**			
	**Length**	**HC PrSc**	**HC tape**	**CrV**
Optiflow Front		<0,001	<0,001	<0,001
Optiflow Back	0.01	<0,001	0.02	0.01

ICC, Intra Class Correlation; HC, Head Circumference; CrV, Cranial Volume

[a] one-way random effects model

[b] two-way random effect model

ICC interpretation:

0.0 - 0.5: Poor reliability

0.5 - 0.75: Moderate reliability

0.75 - 0.9: Good reliability

0.9 - 1.0: Excellent reliability

DISCUSSION

Inter and intrarater variability

Interrater ICC ranged from <0.001 to 0.02, indicating poor reliability between nurses' measurements, for PrSc and measuring tape. This was anticipated for the PrSc, because the placement of anatomical landmarks directly influenced measurement values, and we expected a considerable variance in this between the nurses. In contrast with the poor interrater ICC, intrarater ICC showed moderate to excellent reliability, indicating that a nurse tends to select landmarks consistently at repeated measurements. Intrarater ICC ranged from 0.60 for CrV-Optiflow-back, to 0.97 HC-PrSc Optifow-front, 0.98 Optiflow-back.

The interrater ICC was better for CrV than for HC. This is likely because the calculation script is more sensitive to user input for CrV than for HC. This effect could also explain that PrSc's overall accuracy and precision were higher for HC than CrV.

Limiting for assessing intrarater variability was that, due to the simulated setting, nurses selected the three sets of landmarks directly after each other, with no time in between. The high PrSc intrarater reliability can be considered a strength, representing a good technical instrument precision. It is likely that interrater reliability could be improved with training, considering that nurses used the PrSc for the first time in their lives.

Several studies have used marking of the nasion and tragi for deriving HC and CrV from 3D scans and noted user influence on the measurements [1-5]. Tareq Abdel et al. developed CraniumPy software [6] for assessing head morphology and size, validating its intra- and interrater reliability of the method with full 360-degree, highly detailed scans of infants, though not preterm infants. Their study showed that manual marking of landmarks introduces an intra- and interobserver variability of 3%. Ifflaender et. al. measured HC and CrV of preterm infants and found high agreement between

raters, with mean difference between raters of -0.1 ± 0.74 mm (-0.005±0.2%) for HC and 8.1±6.5 mL (1.5±1.1%) for CrV.

1. Ifflaender, S., et al., *Three-Dimensional Digital Capture of Head Size in Neonates - A Method Evaluation.* PLoS ONE, 2013. **8**(4).
2. Barbero-García, I., et al., *Low-Cost Smartphone-Based Photogrammetry for the Analysis of Cranial Deformation in Infants.* World Neurosurgery, 2017. **102**: p. 545-554.
3. Burkhardt, W., et al., *Non-invasive estimation of brain-volume in infants.* Early Hum Dev, 2019. **132**: p. 52-57.
4. Geil, M.D. and A. Smith, *Accuracy and Reliability of a System for the Digital Capture of Infant Head Shapes in the Treatment of Cranial Deformities.* JPO: Journal of Prosthetics and Orthotics, 2008. **20**(2).
5. Weinberg, S.M., et al., *Anthropometric precision and accuracy of digital three-dimensional photogrammetry: comparing the Genex and 3dMD imaging systems with one another and with direct anthropometry.* J Craniofac Surg, 2006. **17**(3): p. 477-83.
6. Abdel-Alim, T., et al., *Reliability and Agreement of Automated Head Measurements From 3-Dimensional Photogrammetry in Young Children.* Journal of Craniofacial Surgery, 2023.

Supplement 3: Direct comparison of Measuring Tape to PreemieScanner

METHOD

In this supplement we present the direct comparison between head circumference (HC) measurements made with the PreemieScanner versus the standard measuring tape as currently used at the NICU involved in this study. To comply with the approach of comparing a new device to the old, we add the direct comparison of HC measurements done with the standard measuring tape, and the PreemieScanner as a supplement.

Dataset

The 35 nurses each performed three consecutive HC measurements with the tape, placing the tape around the dolls head and reading of the HC from the tape. In addition, the same nurse did three consecutive ‘measurements’ with the PreemieScanner, by placing three sets of anatomical landmarks on the head from which, in a later stage - not by the nurse, HC was derived. To create paired Tape-vs-PreemieScanner measurements for each nurse, we took the mean of the three measurements as the resulting measurement value for either tape or PreemieScanner. This resulted in a dataset of 35 paired Tape - PreemieScanner HC measurements.

Data analysis

For direct comparison of Tape versus PreemieScanner we compared HC measurements performed by each individual nurse. The agreement between the two devices can be interpreted by the HC difference between Tape and PreemieScanner in a paired measurement. A Bland-Altman is suitable for comparing an old versus the new method/instrument, measuring (a population of) real patients (that vary in size), in which case the ground truth is represented by the old, existing technique.

Statistical analysis

A Bland-Altman plot was constructed for visual interpretation of the agreement between the paired measurements.

As comparison metrics, the Pearson correlation coefficient (R), intraclass correlation coefficient (ICC, twoway/agreement/single), Standard Error of Measurement ($SEM=SD\_diff*\sqrt{(1-ICC)}$) and Smallest Detectable Change (SDC) were calculated. The Smallest Detectable Change $SDC=1.96*\sqrt{2}*SEM$ is used as estimate of the minimum change the device can interpret as a real difference, beyond measurement error. In other words, the minimum change in a measurement that must occur for an observer to be confident (with 95% certainty) that the change is not due to measurement error alone.

For completeness, we added the nurses' intra- and interrater variability for HC Tape and Preemie Scanner, by the ICC. Nurses' intrarater ICC was calculated using a one-way random effects model, interrater ICC by a two-way random effects model. The nurses' intra- and interrater variability for all measurement types (BL, HC, CrV) is presented in a seperate supplement 'Inter- and intrarater variability'.

RESULTS

Figure 1 presents the Bland-Altman plot of HC measurements with measuring tape versus PreemieScanner. As expected, the three dolls, having different head sizes, are clearly separated in the plot. Table 1 presents the metrics for direct comparison of Tape with PreemieScanner. ICC of 0.956 is regarded as excellent reliability. Pearson correlation coefficient (R) is 0.981 and Standard Error of Measurement (SEM) 0.052. The Smallest Detectable Change, as device property (smaller is better), is 0.75 cm for tape, and 0.71 cm for PreemieScanner.

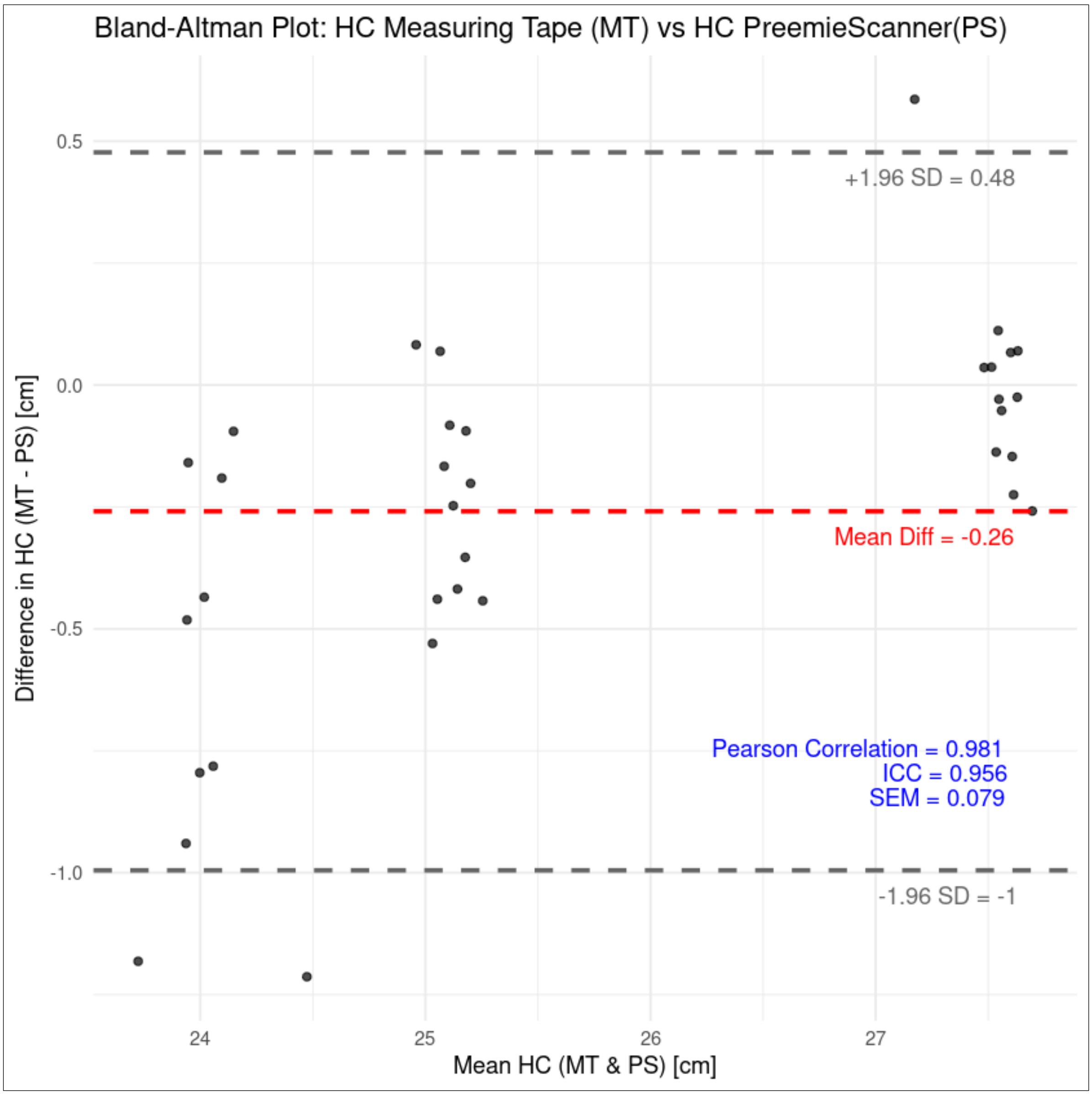

Figure 7. Bland-Altman plot, visualizing the difference between the head circumference (HC) measurement done by the nurses with the measuring tape (MT) and PreemieScanner (PS). Measurements were done on three dolls with different head sizes. Each nurse measured one doll only. Dolls were divided over the nurses, striving for an equal amount of measurements for each doll. Grey dashed lines represent the Limits of Agreement (LoA), representing the range within which 95% of the differences between the two measurement methods are expected to fall, assuming the differences are normally distributed. Each dot represents a nurse's measurement. Pearson Correlation, ICC and Standard Error of Measurement (SEM) were calculated and presented in the plot.

Table 2. Metrics for direct comparison of measuring tape (Tape) and PreemieScanner, for HC measurements.

Single Score Intraclass Correlation[a]	Pearson Correlation (R)	Standard Error of Measurement (SEM=SD_diff*√(1-ICC))	Smallest Detectable Change (SDC=1.96*√2*SEM)
ICC[b]= 0.956<br>95%-Confidence Interval for ICC Population Values: 0.849 < ICC < 0.983	R=0.981	SEM=0.079	SDC Tape= 0.75 cm<br>SDC PreemieScanner= 0.71 cm

[a]Model: twoway, Type : agreement, Subjects (measurements) = 35, Raters (devices) = 2

F-Test, H0: r0 = 0 ; H1: r0 > 0

F(34,8.46) = 64.1 , p = 4.01e-07

[b]ICC interpretation:

0.9 - 1.0: Excellent reliability

Nurses' inter- and intrarater variability Tape vs PreemieScanner

Table 2 shows the interrater ICC to express agreement between nurses' measurements, and intrarater ICC for consistency of the three measurements within the same nurse.

Table 3. Inter and Intrarater variability

Optiflow method	Intrarater ICC[a]		
		HC PreemieScanner	HC tape
Optiflow Front		0.97	0.79
Optiflow Back		0.98	0.78

Optiflow method	Interrater ICC[b]		
		HC PreemieScanner	HC tape
Optiflow Front		<0,001	<0,001
Optiflow Back		<0,001	0.02

ICC, Intra Class Correlation; HC, Head Circumference;

[a] one-way random effects model

[b] two-way random effect model

ICC interpretation:

0.0 - 0.5: Poor reliability

0.5 - 0.75: Moderate reliability

0.75 - 0.9: Good reliability

0.9 - 1.0: Excellent reliability

DISCUSSION

Direct comparison of PreemieScanner and measuring tape performance:

Interpretation of results

Tape and PreemieScanner showed a high correlation of 0.98 and ICC of 0.96, associated with excellent reliability of PreemieScanner to Tape. The spread of error (being the difference between the two paired measurements), represented by the 95% error interval over all dolls, is 1.5 cm. In the Bland-Altman plot it is visible that one of the dolls has a larger error spread than other two dolls. We have no assumable explanation for this, other than the fact that it was the doll with the smallest head, making it more difficult to measure HC relative to larger heads, especially with the tape, which requires manual placement around the head which is more difficult to do on a small head than a larger head. In the Bland-Alt, data points perfectly cluster around the ground truth of the dolls, with doll 2 at the left (GT= 24.1 cm), doll 1 in the middle (GT= 25.1 cm), and doll 3 to the right (GT= 27.8 cm).

Nurses' inter- and intrarater variability for HC Tape and PreemieScanner

Interrater ICC ranged from <0.001 to 0.02, indicating poor reliability between nurses' measurements, for PreemieScanner and measuring tape. This was anticipated for the PreemieScanner, because the placement of anatomical landmarks directly influenced measurement values, and we expected a considerable variance in this between the nurses. In contrast with the poor interrater ICC, intrarater ICC showed moderate to excellent reliability, indicating that a nurse tends to select landmarks consistently at repeated measurements. Intrarater ICC for PreemieScanner showed excellent reliability with 0.97 HC-PreemieScanner Optifow-front, 0.98 Optiflow-back. Intrarater ICC for tape showed good reliability with HC-Tape Optiflow-front 0.79, and Optiflow-back 0.78. In this aspect PreemieScanner outperforms the measuring tape. Limiting for assessing intrarater variability was that, due to the simulated setting, nurses selected the three sets of landmarks directly after each other,

with no time in between. The high PreemieScanner intrarater reliability can be considered a strength, representing a good technical instrument precision. It is likely that interrater reliability could be improved with training, considering that nurses used the PreemieScanner for the first time in their lives.

Direct comparison of techniques versus comparison to ground truth values

Direct comparison of a new technique versus a golden standard, in most cases the commonly used technique in clinical practice is a validation method often used in medical device development. This is understandable when measuring real patients, where often there is no alternative other than the current, standard device, to collect the 'control' measurements to which the new device can be compared to. For our validation study we used dolls instead of real patients, which can be considered a preclinical validation before validation with real patients. In our case, if we had used real preterm infants, these infants should have taken out of their incubators and measured by a medical scanner to acquire accurate ground truth values. This would not be ethical responsible.

To objectively assess clinical accuracy and precision, we strongly believe that comparing a measured value to the true value of the object measured (ground truth), is far preferable than comparing it to another (gold standard) instrument (of which the accuracy and precision is suspected suboptimal). Using dolls allowed us for accurately establishing ground truth values. And with that, comparing the performance of both new and old technique relative to the actual true values.

In the direct comparison we presented Bland-Alt instead of the data plots as presented in the main article. The Bland-Altman plot is often used to compare one measuring instrument to another. The Bland-Altman visualizes the differences between two (paired) measurements, a measurement made with the old, and with the new technique, against the mean of the two values measured. Doing so, the Bland-Altman only shows the performance of the new technique relative to the other, and not relative to the absolute truth, the true value of the object measured. Therefore we did not use Bland-Altman plots in the main article. Instead we visualized the measurement error relative to the fixed

ground truth per doll, and showed the clinically allowed precision limits for a direct interpretation of clinical usability. Also, we bee-swarmed the data points, to enable visual interpretation if data was normally distributed or not.
