# Supplementary material for: Effectiveness of the novel 3D PreemieScanner for preterm infants’ growth monitoring confirmed in a simulated setting
Source: Front Med Technol. 2025 Aug 1;7:1607538. doi: 10.3389/fmedt.2025.1607538 (PMC12354455; doi:10.3389/fmedt.2025.1607538)
Supplement: Supplementary file 6 [file Datasheet6.docx]

Supplement: R-scripts (R v4.4) Data Analysis Preemie Scanner validation

Content

[#R-script BL, HC, CrV average minimum weekly increase of P50 preterm infants & growth data plots 2](#_Toc201744374)

[#R-script statistics, statistical tests and data plots BL,HC, CrV measurements, Tape and PreemieScanner 7](#_Toc201744375)

[#R-Script Tape versus Preemie Scanner direct comparison: BlandAltman, Pearson Correlation, ICC, standard error of measurement SEM, smallest detectable change SDC 65](#_Toc201744376)

[#R-Script Calculation of Standard Error of Measurement SEM and Smallest Detectable Change SDC 69](#_Toc201744377)

# #R-script BL, HC, CrV average minimum weekly increase of P50 preterm infants & growth data plots

#load needed libraries

library(dplyr)

library(ggplot2)

library(readxl)

#summarise the minimum values for weekly increase, per datasource, FOR INFANTS up to 40 wks GA!

weekly_increase %>%

filter(GA < 41) %>% #infants upto 40wks

group_by(Source) %>%

summarise(

min_BL_increase = round(min(BL_Increase, na.rm = TRUE), 3),

min_BL_prop_incr = round(min(BL_Prop_Increase, na.rm = TRUE), 3),

min_HC_increase = round(min(HC_Increase, na.rm = TRUE), 3),

min_HC_prop_incr = round(min(HC_Prop_Increase, na.rm = TRUE), 3),

min_CrV_increase = round(min(CrV_Increase, na.rm = TRUE), 3),

min_CrV_prop_incr = round(min(CrV_Prop_Increase, na.rm = TRUE), 3)

)

#calculate the average of the minimum values per datasource, FOR INFANTS up to 40 wks GA!

weekly_increase %>%

filter(GA < 41) %>% #infants upto 40wks

group_by(Source) %>%

summarise(

min_BL_increase = round(min(BL_Increase, na.rm = TRUE), 3),

min_BL_prop_incr = round(min(BL_Prop_Increase, na.rm = TRUE), 3),

min_HC_increase = round(min(HC_Increase, na.rm = TRUE), 3),

min_HC_prop_incr = round(min(HC_Prop_Increase, na.rm = TRUE), 3),

min_CrV_increase = round(min(CrV_Increase, na.rm = TRUE), 3),

min_CrV_prop_incr = round(min(CrV_Prop_Increase, na.rm = TRUE), 3)

) %>%

# Replace Inf values with NA in the entire dataframe

mutate(across(everything(), ~replace(., is.infinite(.), NA))) %>%

summarise(

mean_min_BL_increase = mean(min_BL_increase, na.rm = TRUE),

mean_min_BL_prop_incr = mean(min_BL_prop_incr, na.rm = TRUE),

mean_min_HC_increase = mean(min_HC_increase, na.rm = TRUE),

mean_min_HC_prop_incr = mean(min_HC_prop_incr, na.rm = TRUE),

mean_min_CrV_increase = mean(min_CrV_increase, na.rm = TRUE),

mean_min_CrV_prop_incr = mean(min_CrV_prop_incr, na.rm = TRUE)

)

#BL P50 data by GA

weekly_increase%>%

#jittered points

ggplot(aes(x=GA, y=BL_P50, color=Source))+geom_jitter()+

#plot title

labs(x = "GA [weeks]", y = "BL P50 [cm]", title = "BL P50 male (M) and female (F), by data source", subtitle="Preterm infant BL data from various sources")+

theme_minimal()+

theme(

legend.position= "bottom",

plot.title = element_text(size = 12, face = "bold"),

plot.subtitle = element_text(size = 10),

plot.caption = element_text(size = 10),

axis.title.x = element_text(size = 10),

axis.title.y = element_text(size = 10),

axis.text.x = element_text(size = 10, face="bold"),

axis.text.y = element_text(size = 10),

text = element_text(family = "sans"))+ facet_wrap(~Sex)

#BL weekly increase by GA

weekly_increase%>%

#jittered points

ggplot(aes(x=GA, y=BL_Increase, color=Source))+geom_jitter()+

#plot title

labs(x = "GA [weeks]", y = "BL weekly increase [cm]", title = "BL weekly increase", subtitle="Preterm infant BL data from various sources")+

theme_minimal()+

theme(

legend.position= "bottom",

plot.title = element_text(size = 12, face = "bold"),

plot.subtitle = element_text(size = 10),

plot.caption = element_text(size = 10),

axis.title.x = element_text(size = 10),

axis.title.y = element_text(size = 10),

axis.text.x = element_text(size = 10, face="bold"),

axis.text.y = element_text(size = 10),

text = element_text(family = "sans"))+ facet_wrap(~Sex)

#PLOTS

#HC P50 data by GA

weekly_increase%>%

#jittered points

ggplot(aes(x=GA, y=HC_P50, color=Source))+geom_jitter()+

#plot title

labs(x = "GA [weeks]", y = "HC P50 [cm]", title = "HC P50 male (M) and female (F), by data source", subtitle="Preterm infant HC data from various sources")+

theme_minimal()+

theme(

legend.position= "bottom",

plot.title = element_text(size = 12, face = "bold"),

plot.subtitle = element_text(size = 10),

plot.caption = element_text(size = 10),

axis.title.x = element_text(size = 10),

axis.title.y = element_text(size = 10),

axis.text.x = element_text(size = 10, face="bold"),

axis.text.y = element_text(size = 10),

text = element_text(family = "sans"))+ facet_wrap(~Sex)

#HC weekly increase by GA

weekly_increase%>%

#jittered points

ggplot(aes(x=GA, y=HC_Increase, color=Source))+geom_jitter()+

#plot title

labs(x = "GA [weeks]", y = "HC weekly increase [cm]", title = "HC weekly increase", subtitle="Preterm infant HC data from various sources")+

theme_minimal()+

theme(

legend.position= "bottom",

plot.title = element_text(size = 12, face = "bold"),

plot.subtitle = element_text(size = 10),

plot.caption = element_text(size = 10),

axis.title.x = element_text(size = 10),

axis.title.y = element_text(size = 10),

axis.text.x = element_text(size = 10, face="bold"),

axis.text.y = element_text(size = 10),

text = element_text(family = "sans"))+ facet_wrap(~Sex)

#CrV P50 data by GA

weekly_increase%>%

#jittered points

ggplot(aes(x=GA, y=CrV_P50, color=Source))+geom_jitter()+

#plot title

labs(x = "GA [weeks]", y = "CrV P50 [mL]", title = "CrV P50 male (M) and female (F), by data source", subtitle="Preterm infant CrV data from various sources")+

theme_minimal()+

theme(

legend.position= "bottom",

plot.title = element_text(size = 12, face = "bold"),

plot.subtitle = element_text(size = 10),

plot.caption = element_text(size = 10),

axis.title.x = element_text(size = 10),

axis.title.y = element_text(size = 10),

axis.text.x = element_text(size = 10, face="bold"),

axis.text.y = element_text(size = 10),

text = element_text(family = "sans"))+ facet_wrap(~Sex)

#CrV weekly increase by GA

weekly_increase%>%

#jittered points

ggplot(aes(x=GA, y=CrV_Increase, color=Source))+geom_jitter()+

#plot title

labs(x = "GA [weeks]", y = "CrV weekly increase [cm]", title = "CrV weekly increase", subtitle="Preterm infant CrV data from various sources")+

theme_minimal()+

theme(

legend.position= "bottom",

plot.title = element_text(size = 12, face = "bold"),

plot.subtitle = element_text(size = 10),

plot.caption = element_text(size = 10),

axis.title.x = element_text(size = 10),

axis.title.y = element_text(size = 10),

axis.text.x = element_text(size = 10, face="bold"),

axis.text.y = element_text(size = 10),

text = element_text(family = "sans"))+ facet_wrap(~Sex)

# #R-script statistics, statistical tests and data plots BL,HC, CrV measurements, Tape and PreemieScanner

# load packages

library(tidyverse)

library(dplyr)

library(here)

library(janitor)

install.packages("ggbeeswarm")

library(ggbeeswarm)

library(readxl)

library(stats)

library(skimr)

install.packages("xlsx")

library(xlsx)

install.packages("writexl")

library(writexl)

library(psych)

#install.packages("lpsolve")

#library(lpSolve)

install.packages("irr")

library(irr)

#library(round)

# define precision border limits

bl_limit<-0.4 #cm

hc_limit<-0.3 #cm

crv_limit<-12 #mL

# Data import ----

Data1 <-read_excel("Data_PreemieScanner.xlsx")

Data2 <-read_excel("Data_PreemieScanner.xlsx", sheet = 2)

groundtruthdata <-read_excel("GroundTruth.xlsx")

# Data filtering ----

cleandata1 <- Data1 %>%

rename(

measurement = `Measurement m#`,

crv_valid = 'CrV At Least 1 Valid Merge',

hc_valid = 'HC At Least 1 Valid Merge',

optiflow_clear = 'Optiflow attached to front/back of the head'

) %>%

select_all(tolower) %>%

clean_names()

cleandata1$hc = round_half_up(cleandata1$hc, digits = 1)

cleandata1$bl = round_half_up(cleandata1$bl, digits = 1)

cleandata2 <- Data2 %>%

select_all(tolower) %>%

clean_names()

groundtruthdata[, 2:4] = round_half_up(groundtruthdata[, 2:4], digits = 1)

PS_alleen <- cleandata1 %>%

filter (device == "PS")

HM_alleen <- cleandata1 %>%

filter (device == "HM")

Ellen_alleen <- cleandata1 %>%

filter (doll == "Ellen")

Sophie_alleen <- cleandata1 %>%

filter (doll == "Sophie")

To_alleen <- cleandata1 %>%

filter (doll == "To")

beademingnaarachter <- cleandata1 %>%

filter (optiflow_clear == "back")

beademingnaarvoren <- cleandata1 %>%

filter (optiflow_clear == "front")

# Mutate: om varibalen te transformeren

# Calculate CrV mean

# Dit is al in de excel gezet die ingeladen wordt, waardoor dit al verwerkt is in Data 1 (en dus de rest van dit script)

crv_mean <- cleandata2 %>%

mutate(crv_mean_valid = (crv_d*crv_d_valid_merge+crv_c*crv_c_valid_merge+crv_b*crv_b_valid_merge+crv_a*crv_a_valid_merge)/(crv_d_valid_merge+crv_c_valid_merge+crv_b_valid_merge+crv_a_valid_merge), na.rm = TRUE) %>%

mutate(crv_mean_valid = round_half_up(crv_mean_valid, digits = 1))

# Write cleaned data to excel

write_xlsx(crv_mean, "crv_mean.xlsx")

# Calculate deviation

combined_data <- merge(cleandata1, groundtruthdata, by = "doll")

Deviation <- combined_data %>%

arrange(id) %>%

mutate(deviation_bl = bl.x - bl.y, na.rm = TRUE) %>%

mutate (deviation_hc = hc.x - hc.y, na.rm = TRUE) %>%

mutate (deviation_crv = crv.x - crv.y, na.rm = TRUE) %>%

mutate (deviation_bl_percentage = (bl.x - bl.y) / bl.y * 100, na.rm = TRUE) %>%

mutate (deviation_hc_percentage = (hc.x - hc.y) / hc.y * 100, na.rm = TRUE) %>%

mutate (deviation_crv_percentage = (crv.x - crv.y) / crv.y * 100, na.rm = TRUE)

#Deviation$deviation_bl_percentage = round_half_up(Deviation$deviation_bl_percentage, digits = 1)

#Deviation$deviation_hc_percentage = round_half_up(Deviation$deviation_hc_percentage, digits = 1)

#Deviation$deviation_crv_percentage = round_half_up(Deviation$deviation_crv_percentage, digits = 1)

# Data sorteren

DeviationPS_alleen <- Deviation %>%

filter (device == "PS")

DeviationHM_alleen <- Deviation %>%

filter (device == "HM")

Deviation_beademingnaarachter <- Deviation %>%

filter (optiflow_clear == "back")

Deviation_beademingnaarvoren <- Deviation %>%

filter (optiflow_clear == "front")

Deviation_PS_beademingnaarachter <- Deviation %>%

filter (device == "PS" & optiflow_clear == "back")

Deviation_HM_beademingnaarachter <- Deviation %>%

filter (device == "HM" & optiflow_clear == "back")

Deviation_PS_beademingnaarvoren <- Deviation %>%

filter (device == "PS" & optiflow_clear == "front")

Deviation_HM_beademingnaarvoren <- Deviation %>%

filter (device == "HM" & optiflow_clear == "front")

Deviation_PS_Ellen <- Deviation %>%

filter (device == "PS" & doll == "Ellen")

Deviation_PS_Sophie <- Deviation %>%

filter (device == "PS" & doll == "Sophie")

Deviation_PS_To <- Deviation %>%

filter (device == "PS" & doll == "To")

PS_alleen_combineddata <- combined_data %>%

filter (device == "PS") %>%

mutate(combined_optiflowclear_doll = paste(optiflow_clear, doll, sep = " "), na.rm = TRUE)

HM_alleen_combineddata <- combined_data %>%

filter (device == "HM") %>%

mutate(combined_optiflowclear_doll = paste(optiflow_clear, doll, sep = " "), na.rm = TRUE)

Deviation_PS_Ellen_beademingnaarvoren <- Deviation_PS_beademingnaarvoren %>%

filter (doll == "Ellen")

Deviation_HM_Ellen_beademingnaarvoren <- Deviation_HM_beademingnaarvoren %>%

filter (doll == "Ellen")

Deviation_PS_Ellen_beademingnaarachter <- Deviation_PS_beademingnaarachter %>%

filter (doll == "Ellen")

Deviation_HM_Ellen_beademingnaarachter <- Deviation_HM_beademingnaarachter %>%

filter (doll == "Ellen")

Deviation_PS_Sophie_beademingnaarvoren <- Deviation_PS_beademingnaarvoren %>%

filter (doll == "Sophie")

Deviation_HM_Sophie_beademingnaarvoren <- Deviation_HM_beademingnaarvoren %>%

filter (doll == "Sophie")

Deviation_PS_Sophie_beademingnaarachter <- Deviation_PS_beademingnaarachter %>%

filter (doll == "Sophie")

Deviation_HM_Sophie_beademingnaarachter <- Deviation_HM_beademingnaarachter %>%

filter (doll == "Sophie")

Deviation_PS_To_beademingnaarvoren <- Deviation_PS_beademingnaarvoren %>%

filter (doll == "To")

Deviation_HM_To_beademingnaarvoren <- Deviation_HM_beademingnaarvoren %>%

filter (doll == "To")

Deviation_PS_To_beademingnaarachter <- Deviation_PS_beademingnaarachter %>%

filter (doll == "To")

Deviation_HM_To_beademingnaarachter <- Deviation_HM_beademingnaarachter %>%

filter (doll == "To")

groundtruthdata_Ellen <- groundtruthdata %>%

filter (doll == "Ellen")

groundtruthdata_Sophie <- groundtruthdata %>%

filter (doll == "Sophie")

groundtruthdata_To <- groundtruthdata %>%

filter (doll == "To")

# summary --------

summary_Deviation <- Deviation %>%

group_by(device, optiflow_clear) %>%

summarise(mean_dev_bl = mean(deviation_bl, na.rm = TRUE),

mean_dev_hc = mean(deviation_hc, na.rm = TRUE),

mean_dev_crv = mean(deviation_crv, na.rm = TRUE),

median_dev_bl = median(deviation_bl, na.rm = TRUE),

median_dev_hc = median(deviation_hc, na.rm = TRUE),

median_dev_crv = median(deviation_crv, na.rm = TRUE),

sd_dev_bl = sd(deviation_bl, na.rm = TRUE),

sd_dev_hc = sd(deviation_hc, na.rm = TRUE),

sd_dev_crv = sd(deviation_crv, na.rm = TRUE),

maximum_dev_bl = max(deviation_bl, na.rm = TRUE),

minimum_dev_bl = min(deviation_bl, na.rm = TRUE),

maximum_dev_hc = max(deviation_hc, na.rm = TRUE),

minimum_dev_hc = min(deviation_hc, na.rm = TRUE),

maximum_dev_crv = max(deviation_crv, na.rm = TRUE),

minimum_dev_crv = min(deviation_crv, na.rm = TRUE))

summary_Deviation_doll <- Deviation %>%

group_by(device, optiflow_clear, doll) %>%

summarise(mean_dev_bl = mean(deviation_bl, na.rm = TRUE),

mean_dev_hc = mean(deviation_hc, na.rm = TRUE),

mean_dev_crv = mean(deviation_crv, na.rm = TRUE),

median_dev_bl = median(deviation_bl, na.rm = TRUE),

median_dev_hc = median(deviation_hc, na.rm = TRUE),

median_dev_crv = median(deviation_crv, na.rm = TRUE),

maximum_dev_bl = max(deviation_bl, na.rm = TRUE),

minimum_dev_bl = min(deviation_bl, na.rm = TRUE),

maximum_dev_hc = max(deviation_hc, na.rm = TRUE),

minimum_dev_hc = min(deviation_hc, na.rm = TRUE),

maximum_dev_crv = max(deviation_crv, na.rm = TRUE),

minimum_dev_crv = min(deviation_crv, na.rm = TRUE))

summary_Deviation_percentage_doll <- Deviation %>%

group_by(device, optiflow_clear, doll) %>%

summarise(mean_dev_bl = mean(deviation_bl_percentage, na.rm = TRUE),

mean_dev_hc = mean(deviation_hc_percentage, na.rm = TRUE),

mean_dev_crv = mean(deviation_crv_percentage, na.rm = TRUE),

median_dev_bl = median(deviation_bl_percentage, na.rm = TRUE),

median_dev_hc = median(deviation_hc_percentage, na.rm = TRUE),

median_dev_crv = median(deviation_crv_percentage,na.rm = TRUE),

maximum_dev_bl = max(deviation_bl_percentage, na.rm = TRUE),

minimum_dev_bl = min(deviation_bl_percentage, na.rm = TRUE),

maximum_dev_hc = max(deviation_hc_percentage, na.rm = TRUE),

minimum_dev_hc = min(deviation_hc_percentage, na.rm = TRUE),

maximum_dev_crv = max(deviation_crv_percentage, na.rm = TRUE),

minimum_dev_crv = min(deviation_crv_percentage, na.rm = TRUE))

summary_Deviation_percentage <- Deviation %>%

group_by(device, doll) %>%

summarise(mean_dev_bl = mean(deviation_bl_percentage, na.rm = TRUE),

mean_dev_hc = mean(deviation_hc_percentage, na.rm = TRUE),

mean_dev_crv = mean(deviation_crv_percentage, na.rm = TRUE),

median_dev_bl = median(deviation_bl_percentage, na.rm = TRUE),

median_dev_hc = median(deviation_hc_percentage, na.rm = TRUE),

median_dev_crv = median(deviation_crv_percentage,na.rm = TRUE),

maximum_dev_bl = max(deviation_bl_percentage, na.rm = TRUE),

minimum_dev_bl = min(deviation_bl_percentage, na.rm = TRUE),

maximum_dev_hc = max(deviation_hc_percentage, na.rm = TRUE),

minimum_dev_hc = min(deviation_hc_percentage, na.rm = TRUE),

maximum_dev_crv = max(deviation_crv_percentage, na.rm = TRUE),

minimum_dev_crv = min(deviation_crv_percentage, na.rm = TRUE))

summary_Deviation_percentage_device <- Deviation %>%

group_by(device) %>%

summarise(mean_dev_bl = mean(deviation_bl_percentage, na.rm = TRUE),

mean_dev_hc = mean(deviation_hc_percentage, na.rm = TRUE),

mean_dev_crv = mean(deviation_crv_percentage, na.rm = TRUE),

median_dev_bl = median(deviation_bl_percentage, na.rm = TRUE),

median_dev_hc = median(deviation_hc_percentage, na.rm = TRUE),

median_dev_crv = median(deviation_crv_percentage,na.rm = TRUE),

maximum_dev_bl = max(deviation_bl_percentage, na.rm = TRUE),

minimum_dev_bl = min(deviation_bl_percentage, na.rm = TRUE),

maximum_dev_hc = max(deviation_hc_percentage, na.rm = TRUE),

minimum_dev_hc = min(deviation_hc_percentage, na.rm = TRUE),

maximum_dev_crv = max(deviation_crv_percentage, na.rm = TRUE),

minimum_dev_crv = min(deviation_crv_percentage, na.rm = TRUE))

summary_Deviation_percentage_beademinggesplitst <- Deviation %>%

group_by(device, optiflow_clear) %>%

summarise(mean_dev_bl = mean(deviation_bl_percentage, na.rm = TRUE),

mean_dev_hc = mean(deviation_hc_percentage, na.rm = TRUE),

mean_dev_crv = mean(deviation_crv_percentage, na.rm = TRUE),

median_dev_bl = median(deviation_bl_percentage, na.rm = TRUE),

median_dev_hc = median(deviation_hc_percentage, na.rm = TRUE),

median_dev_crv = median(deviation_crv_percentage,na.rm = TRUE))

# Shapiro test for normality (p-waarde less than 0.05 means non normally distributed, 0.05 or more normally distributed) ---------

shapiro_test_result <- list(

bl_beademingfront = shapiro.test(Deviation_PS_beademingnaarvoren$deviation_bl),

bl_beademingback = shapiro.test(Deviation_PS_beademingnaarachter$deviation_bl),

hc_PS_beademingfront = shapiro.test(Deviation_PS_beademingnaarvoren$deviation_hc),

hc_PS_beademingback = shapiro.test(Deviation_PS_beademingnaarachter$deviation_hc),

hc_HM_beademingfront = shapiro.test(Deviation_HM_beademingnaarvoren$deviation_hc),

hc_HM_beademingback = shapiro.test(Deviation_HM_beademingnaarachter$deviation_hc),

crv_beademingfront = shapiro.test(Deviation_PS_beademingnaarvoren$deviation_crv),

crv_beademinback = shapiro.test(Deviation_PS_beademingnaarachter$deviation_crv))

shapiro_test_result_percentage <- list(

bl = shapiro.test(DeviationPS_alleen$deviation_bl_percentage),

hc_PS_beademingfront = shapiro.test(Deviation_PS_beademingnaarvoren$deviation_hc_percentage),

hc_PS_beademingback = shapiro.test(Deviation_PS_beademingnaarachter$deviation_hc_percentage),

hc_HM_beademingfront = shapiro.test(Deviation_HM_beademingnaarvoren$deviation_hc_percentage),

hc_HM_beademingback = shapiro.test(Deviation_HM_beademingnaarachter$deviation_hc_percentage),

crv_beademingfront = shapiro.test(Deviation_PS_beademingnaarvoren$deviation_crv_percentage),

crv_beademinback = shapiro.test(Deviation_PS_beademingnaarachter$deviation_crv_percentage))

shapiro_test_result_percentage_perpop <- list(

bl_Ellen = shapiro.test(Deviation_PS_Ellen$deviation_bl_percentage),

bl_Sophie = shapiro.test(Deviation_PS_Sophie$deviation_bl_percentage),

bl_To = shapiro.test(Deviation_PS_To$deviation_bl_percentage),

hc_PS_beademingfront_Ellen = shapiro.test(Deviation_PS_Ellen_beademingnaarvoren$deviation_hc_percentage),

hc_PS_beademingfront_Sophie = shapiro.test(Deviation_PS_Sophie_beademingnaarvoren$deviation_hc_percentage),

hc_PS_beademingfront_To = shapiro.test(Deviation_PS_To_beademingnaarvoren$deviation_hc_percentage),

hc_PS_beademingback_Ellen = shapiro.test(Deviation_PS_Ellen_beademingnaarachter$deviation_hc_percentage),

hc_PS_beademingback_Sophie = shapiro.test(Deviation_PS_Sophie_beademingnaarachter$deviation_hc_percentage),

hc_PS_beademingback_To = shapiro.test(Deviation_PS_To_beademingnaarachter$deviation_hc_percentage),

hc_HM_beademingfront_Ellen = shapiro.test(Deviation_HM_Ellen_beademingnaarvoren$deviation_hc_percentage),

hc_HM_beademingfront_Sophie = shapiro.test(Deviation_HM_Sophie_beademingnaarvoren$deviation_hc_percentage),

hc_HM_beademingfront_To = shapiro.test(Deviation_HM_To_beademingnaarvoren$deviation_hc_percentage),

hc_HM_beademingback_Ellen = shapiro.test(Deviation_HM_Ellen_beademingnaarachter$deviation_hc_percentage),

hc_HM_beademingback_Sophie = shapiro.test(Deviation_HM_Sophie_beademingnaarachter$deviation_hc_percentage),

hc_HM_beademingback_To = shapiro.test(Deviation_HM_To_beademingnaarachter$deviation_hc_percentage),

crv_beademingfront_Ellen = shapiro.test(Deviation_PS_Ellen_beademingnaarvoren$deviation_crv_percentage),

crv_beademingfront_Sophie = shapiro.test(Deviation_PS_Sophie_beademingnaarvoren$deviation_crv_percentage),

crv_beademingfront_To = shapiro.test(Deviation_PS_To_beademingnaarvoren$deviation_crv_percentage),

crv_beademinback_Ellen = shapiro.test(Deviation_PS_Ellen_beademingnaarachter$deviation_crv_percentage),

crv_beademinback_Sophie = shapiro.test(Deviation_PS_Sophie_beademingnaarachter$deviation_crv_percentage),

crv_beademinback_To = shapiro.test(Deviation_PS_To_beademingnaarachter$deviation_crv_percentage))

# t-test, when normal distribution or Wilcoxon signed rank test when not normally ----------

# Het is niet normaal verdeeld, dus: tekentest

# Ook 95% betrouwbaarheidsinterval

# nulhypothese = waarden zijn gelijk aan 0, bij p-waarden minder dan 0.05 betekent dat het significant verschillend is

# wilcoxon test voor de percentage afwijking --------------

tekentest_result_percentage <- list(

bl = wilcox.test(DeviationPS_alleen$deviation_bl_percentage, mu=0,exact = FALSE),

crv_beademingfront = wilcox.test(Deviation_PS_beademingnaarvoren$deviation_crv_percentage, mu=0,exact = FALSE),

crv_beademingback = t.test(Deviation_PS_beademingnaarachter$deviation_crv_percentage, mu=0, conf.int = TRUE, conf.level = 0.95),

hcPS_beademingfront = wilcox.test(Deviation_PS_beademingnaarvoren$deviation_hc_percentage, mu=0,exact = FALSE),

hcPS_beademingback = wilcox.test(Deviation_PS_beademingnaarachter$deviation_hc_percentage, mu=0,exact = FALSE),

hcHM_beademingfront = wilcox.test(Deviation_HM_beademingnaarvoren$deviation_hc_percentage, mu=0,exact = FALSE),

hcHM_beademingback = wilcox.test(Deviation_HM_beademingnaarachter$deviation_hc_percentage, mu=0,exact = FALSE))

print(tekentest_result_percentage)

# wilcoxon test voor

tekentest_result_beademing <- list(

crv = wilcox.test(Deviation_PS_beademingnaarvoren$deviation_crv_percentage, Deviation_PS_beademingnaarachter$deviation_crv_percentage, exact = FALSE),

hcPS = wilcox.test(Deviation_PS_beademingnaarvoren$deviation_hc_percentage, Deviation_PS_beademingnaarachter$deviation_hc_percentage, exact = FALSE),

hcHM = wilcox.test(Deviation_HM_beademingnaarvoren$deviation_hc_percentage, Deviation_HM_beademingnaarachter$deviation_hc_percentage, exact = FALSE)

)

# 95% intervals for normal data ---------------

crv_sd_beademingback <- sd(Deviation_PS_beademingnaarachter$deviation_crv_percentage, na.rm = TRUE)

crv_low_beademingback <- mean(Deviation_PS_beademingnaarachter$deviation_crv_percentage, na.rm = TRUE) - 1.96*crv_sd_beademingback

crv_high_beademingback <- mean(Deviation_PS_beademingnaarachter$deviation_crv_percentage, na.rm = TRUE) + 1.96*crv_sd_beademingback

crv_sd_Ellen_beademingback <- sd(Deviation_PS_Ellen_beademingnaarachter$deviation_crv_percentage, na.rm = TRUE)

crv_low_Ellen_beademingback <- mean(Deviation_PS_Ellen_beademingnaarachter$deviation_crv_percentage, na.rm = TRUE) - 1.96*crv_sd_Ellen_beademingback

crv_high_Ellen_beademingback <- mean(Deviation_PS_Ellen_beademingnaarachter$deviation_crv_percentage, na.rm = TRUE) + 1.96*crv_sd_Ellen_beademingback

crv_sd_Sophie_beademingback <- sd(Deviation_PS_Sophie_beademingnaarachter$deviation_crv_percentage, na.rm = TRUE)

crv_low_Sophie_beademingback <- mean(Deviation_PS_Sophie_beademingnaarachter$deviation_crv_percentage, na.rm = TRUE) - 1.96*crv_sd_Sophie_beademingback

crv_high_Sophie_beademingback <- mean(Deviation_PS_Sophie_beademingnaarachter$deviation_crv_percentage,na.rm = TRUE) + 1.96*crv_sd_Sophie_beademingback

crv_sd_Ellen_beademingfront <- sd(Deviation_PS_Ellen_beademingnaarvoren$deviation_crv_percentage, na.rm = TRUE)

crv_low_Ellen_beademingfront <- mean(Deviation_PS_Ellen_beademingnaarvoren$deviation_crv_percentage, na.rm = TRUE) - 1.96*crv_sd_Ellen_beademingfront

crv_high_Ellen_beademingfront <- mean(Deviation_PS_Ellen_beademingnaarvoren$deviation_crv_percentage, na.rm = TRUE) + 1.96*crv_sd_Ellen_beademingfront

crv_sd_Sophie_beademingfront <- sd(Deviation_PS_Sophie_beademingnaarvoren$deviation_crv_percentage, na.rm = TRUE)

crv_low_Sophie_beademingfront <- mean(Deviation_PS_Sophie_beademingnaarvoren$deviation_crv_percentage, na.rm = TRUE) - 1.96*crv_sd_Sophie_beademingfront

crv_high_Sophie_beademingfront <- mean(Deviation_PS_Sophie_beademingnaarvoren$deviation_crv_percentage,na.rm = TRUE) + 1.96*crv_sd_Sophie_beademingfront

hcHM_sd_Ellen_beademingfront <- sd(Deviation_HM_Ellen_beademingnaarvoren$deviation_hc_percentage, na.rm = TRUE)

hcHM_low_Ellen_beademingfront <- mean(Deviation_HM_Ellen_beademingnaarvoren$deviation_hc_percentage, na.rm = TRUE) - 1.96*hcHM_sd_Ellen_beademingfront

hcHM_high_Ellen_beademingfront <- mean(Deviation_HM_Ellen_beademingnaarvoren$deviation_hc_percentage, na.rm = TRUE) + 1.96*hcHM_sd_Ellen_beademingfront

hcHM_sd_Ellen_beademingback <- sd(Deviation_HM_Ellen_beademingnaarachter$deviation_hc_percentage, na.rm = TRUE)

hcHM_low_Ellen_beademingback <- mean(Deviation_HM_Ellen_beademingnaarachter$deviation_hc_percentage, na.rm = TRUE) - 1.96*hcHM_sd_Ellen_beademingback

hcHM_high_Ellen_beademingback <- mean(Deviation_HM_Ellen_beademingnaarachter$deviation_hc_percentage, na.rm = TRUE) + 1.96*hcHM_sd_Ellen_beademingback

hcHM_sd_Sophie_beademingback <- sd(Deviation_HM_Sophie_beademingnaarachter$deviation_hc_percentage, na.rm = TRUE)

hcHM_low_Sophie_beademingback <- mean(Deviation_HM_Sophie_beademingnaarachter$deviation_hc_percentage, na.rm = TRUE) - 1.96*hcHM_sd_Sophie_beademingback

hcHM_high_Sophie_beademingback <- mean(Deviation_HM_Sophie_beademingnaarachter$deviation_hc_percentage,na.rm = TRUE) + 1.96*hcHM_sd_Sophie_beademingback

bl_sd_Sophie <- sd(Deviation_PS_Sophie$deviation_bl_percentage, na.rm = TRUE)

bl_low_Sophie <- mean(Deviation_PS_Sophie$deviation_bl_percentage, na.rm = TRUE) - 1.96*bl_sd_Sophie

bl_high_Sophie <- mean(Deviation_PS_Sophie$deviation_bl_percentage, na.rm = TRUE) + 1.96*bl_sd_Sophie

# percentielen / 95% CI ---------------------

percentiel_resultaten <- list(

bl = quantile(DeviationPS_alleen$deviation_bl_percentage, probs = c(0.025, 0.975), na.rm = TRUE),

bl_beademingback = quantile(Deviation_PS_beademingnaarachter$deviation_bl_percentage, probs = c(0.025, 0.975), na.rm = TRUE),

crv_beademingfront = quantile(Deviation_PS_beademingnaarvoren$deviation_crv_percentage, probs = c(0.025, 0.975), na.rm = TRUE),

crv_beademingback = c(crv_low_beademingback, crv_high_beademingback),

hcPS_beademingfront = quantile(Deviation_PS_beademingnaarvoren$deviation_hc_percentage, probs = c(0.025, 0.975), na.rm = TRUE),

hcPS_beademingback = quantile(Deviation_PS_beademingnaarachter$deviation_hc_percentage, probs = c(0.025, 0.975), na.rm = TRUE),

hcHM_beademingfront = quantile(Deviation_HM_beademingnaarvoren$deviation_hc_percentage, probs = c(0.025, 0.975), na.rm = TRUE),

hcHM_beademingback = quantile(Deviation_HM_beademingnaarachter$deviation_hc_percentage, probs = c(0.025, 0.975), na.rm = TRUE))

percentiel_resultaten_pop <- list(

bl_Ellen = quantile(Deviation_PS_Ellen$deviation_bl_percentage, probs = c(0.025, 0.975), na.rm = TRUE),

crv_beademingfrontEllen = c(crv_low_Ellen_beademingfront, crv_high_Ellen_beademingfront),

crv_beademingbackEllen = c(crv_low_Ellen_beademingback, crv_high_Ellen_beademingback),

hcPS_beademingfrontEllen = quantile(Deviation_PS_Ellen_beademingnaarvoren$deviation_hc_percentage, probs = c(0.025, 0.975), na.rm = TRUE),

hcPS_beademingbackEllen = quantile(Deviation_PS_Ellen_beademingnaarachter$deviation_hc_percentage, probs = c(0.025, 0.975), na.rm = TRUE),

hcHM_beademingfrontEllen = c(hcHM_low_Ellen_beademingfront, hcHM_high_Ellen_beademingfront),

hcHM_beademingbackEllen = c(hcHM_low_Ellen_beademingback, hcHM_high_Ellen_beademingback),

bl_Sophie = c(bl_low_Sophie, bl_high_Sophie),

crv_beademingfrontSophie = c(crv_low_Sophie_beademingfront, crv_high_Sophie_beademingfront),

crv_beademingbackSophie = c(crv_low_Sophie_beademingback, crv_high_Sophie_beademingback),

hcPS_beademingfrontSophie = quantile(Deviation_PS_Sophie_beademingnaarvoren$deviation_hc_percentage, probs = c(0.025, 0.975), na.rm = TRUE),

hcPS_beademingbackSophie = quantile(Deviation_PS_Sophie_beademingnaarachter$deviation_hc_percentage, probs = c(0.025, 0.975), na.rm = TRUE),

hcHM_beademingfrontSophie = quantile(Deviation_HM_Sophie_beademingnaarvoren$deviation_hc_percentage, probs = c(0.025, 0.975), na.rm = TRUE),

hcHM_beademingbackSophie = c(hcHM_low_Sophie_beademingback, hcHM_high_Sophie_beademingback),

bl_To = quantile(Deviation_PS_To$deviation_bl_percentage, probs = c(0.025, 0.975), na.rm = TRUE),

bl_beademingbackTo = quantile(Deviation_PS_To_beademingnaarachter$deviation_bl_percentage, probs = c(0.025, 0.975), na.rm = TRUE),

crv_beademingfrontTo = quantile(Deviation_PS_To_beademingnaarvoren$deviation_crv_percentage, probs = c(0.025, 0.975), na.rm = TRUE),

crv_beademingbackTo = quantile(Deviation_PS_To_beademingnaarachter$deviation_crv_percentage, probs = c(0.025, 0.975), na.rm = TRUE),

hcPS_beademingfrontTo = quantile(Deviation_PS_To_beademingnaarvoren$deviation_hc_percentage, probs = c(0.025, 0.975), na.rm = TRUE),

hcPS_beademingbackTo = quantile(Deviation_PS_To_beademingnaarachter$deviation_hc_percentage, probs = c(0.025, 0.975), na.rm = TRUE),

hcHM_beademingfrontTo = quantile(Deviation_HM_To_beademingnaarvoren$deviation_hc_percentage, probs = c(0.025, 0.975), na.rm = TRUE),

hcHM_beademingbackTo = quantile(Deviation_HM_To_beademingnaarachter$deviation_hc_percentage, probs = c(0.025, 0.975), na.rm = TRUE))

# plotten measured data en ground truth per doll -------------

# plot measured Body Length

PS_alleen_combineddata$doll <- factor(PS_alleen_combineddata$doll, levels = c("Sophie", "Ellen", "To"))

PS_alleen_combineddata %>%

ggplot(aes(x = doll)) +

geom_beeswarm(aes(y = bl.x, shape = "bl.x", color = "bl.x"), cex = 1.5, size=1.5) +

geom_point(aes(y = bl.y, shape = "bl.y", color = "bl.y"), size=3) +

theme_classic() +

labs(

title = "Body Length PreemieScanner",

x = "Dolls",

y = "Measured length (cm)",

shape = "Measurement type",

color = "Measurement type") +

scale_x_discrete(labels = c("Sophie" = "Doll 1",

"Ellen" = "Doll 2",

"To" = "Doll 3 ")) +

scale_shape_manual(

values = c("bl.x" = 16, "bl.y" = 17),

labels = c("bl.x" = "Nurses", "bl.y" = "Ground truth"),

name = "Measurement type") +

scale_color_manual(

values = c("bl.x" = "tomato1", "bl.y" = "black"),

labels = c("bl.x" = "Nurses", "bl.y" = "Ground truth"),

name = "Measurement type") +

guides(

color = guide_legend(

override.aes = list(

shape = c(16, 17),

color = c("tomato1", "black")),

title = "Measurement type",

labels = c("Nurses", "Ground truth")))+

scale_y_continuous (

limits = c(28, 36),

breaks = c(28, 29, 30, 31, 32, 33, 34, 35, 36)

) +

theme(

panel.grid.minor = element_line(color = "lightgray", size = 0.1),

panel.grid.major = element_line(color = "lightgray", size = 0.1),

legend.box = "vertical"

)

# plot measured Head Circumference PreemieScanner

PS_alleen_combineddata$combined_optiflowclear_doll <- factor(PS_alleen_combineddata$combined_optiflowclear_doll, levels = c("back Sophie", "front Sophie", "back Ellen", "front Ellen", "back To", "front To"))

PS_alleen_combineddata %>%

ggplot(aes(x = combined_optiflowclear_doll)) +

geom_beeswarm(aes(y = hc.x, shape = "hc.x", color = "hc.x"), cex = 1.5, size=1.5) +

geom_point(aes(y = hc.y, shape = "hc.y", color = "hc.y"),size=3) +

theme_classic() +

labs(

title = "Head Circumference PreemieScanner",

x = "Doll and breathing tube system",

y = "Measured Head Circumference (cm)",

shape = "Measurement type",

color = "Measurement type") +

scale_x_discrete(labels = c("back Sophie" = "Doll\u00A01,\noptiflow \nback",

"back Ellen" = "Doll\u00A02, \noptiflow \nback",

"back To" = "Doll\u00A03, \noptiflow \nback",

"front Sophie" = "Doll\u00A01, \noptiflow \nfront",

"front Ellen" = "Doll\u00A02, \noptiflow \nfront",

"front To" = "Doll\u00A03, \noptiflow \nfront")) +

scale_shape_manual(

values = c("hc.x" = 16, "hc.y" = 17),

labels = c("hc.x" = "Nurses", "hc.y" = "Ground truth"),

name = "Measurement type") +

scale_color_manual(

values = c("hc.x" = "deepskyblue", "hc.y" = "black"),

labels = c("hc.x" = "Nurses", "hc.y" = "Ground truth"),

name = "Measurement type") +

guides(

color = guide_legend(

override.aes = list(

shape = c(16, 17),

color = c("deepskyblue", "black")),

title = "Measurement type",

labels = c("Nurses", "Ground truth")))+

scale_y_continuous (

limits = c(23, 29),

breaks = c(23, 24, 25, 26, 27, 28, 29)

) +

theme(

panel.grid.minor = element_line(color = "lightgray", size = 0.1),

panel.grid.major = element_line(color = "lightgray", size = 0.1),

legend.box = "vertical"

)

# plot measured Head Circumference Measuring Tape

HM_alleen_combineddata$combined_optiflowclear_doll <- factor(HM_alleen_combineddata$combined_optiflowclear_doll, levels = c("back Sophie", "front Sophie", "back Ellen", "front Ellen", "back To", "front To"))

HM_alleen_combineddata %>%

ggplot(aes(x = combined_optiflowclear_doll)) +

geom_beeswarm(aes(y = hc.x, shape = "hc.x", color = "hc.x"), cex = 1.1, size=1.5) +

geom_point(aes(y = hc.y, shape = "hc.y", color = "hc.y"),size=3) +

theme_classic() +

labs(

title = "Head Circumference Measuring Tape",

x = "Doll and breathing tube system",

y = "Measured Head Circumference (cm)",

shape = "Measurement type",

color = "Measurement type") +

scale_x_discrete(labels = c("back Sophie" = "Doll\u00A01,\noptiflow \nback",

"back Ellen" = "Doll\u00A02, \noptiflow \nback",

"back To" = "Doll\u00A03, \noptiflow \nback",

"front Sophie" = "Doll\u00A01, \noptiflow \nfront",

"front Ellen" = "Doll\u00A02, \noptiflow \nfront",

"front To" = "Doll\u00A03, \noptiflow \nfront")) +

scale_shape_manual(

values = c("hc.x" = 16, "hc.y" = 17),

labels = c("hc.x" = "Nurses", "hc.y" = "Ground truth"),

name = "Measurement type") +

scale_color_manual(

values = c("hc.x" = "plum", "hc.y" = "black"),

labels = c("hc.x" = "Nurses", "hc.y" = "Ground truth"),

name = "Measurement type") +

guides(

color = guide_legend(

override.aes = list(

shape = c(16, 17),

color = c("plum", "black")),

title = "Measurement type",

labels = c("Nurses", "Ground truth")))+

scale_y_continuous (

limits = c(22, 29),

breaks = c(22, 23, 24, 25, 26, 27, 28, 29)

) +

theme(

panel.grid.minor = element_line(color = "lightgray", size = 0.1),

panel.grid.major = element_line(color = "lightgray", size = 0.1),

legend.box = "vertical"

)

# plot measured Cranial Volume PreemieScanner

PS_alleen_combineddata$combined_optiflowclear_doll <- factor(PS_alleen_combineddata$combined_optiflowclear_doll, levels = c("back Sophie", "front Sophie", "back Ellen", "front Ellen", "back To", "front To"))

PS_alleen_combineddata %>%

ggplot(aes(x = combined_optiflowclear_doll)) +

geom_beeswarm(aes(y = crv.x, shape = "crv.x", color = "crv.x"), cex = 1.2, size=1.5) +

geom_point(aes(y = crv.y, shape = "crv.y", color = "crv.y"),size=3) +

theme_classic() +

labs(

title = "Cranial Volume PreemieScanner",

x = "Doll and breathing tube system",

y = "Measured Cranial Volume (mL)",

shape = "Measurement type",

color = "Measurement type") +

scale_x_discrete(labels = c("back Sophie" = "Doll\u00A01,\noptiflow \nback",

"back Ellen" = "Doll\u00A02, \noptiflow \nback",

"back To" = "Doll\u00A03, \noptiflow \nback",

"front Sophie" = "Doll\u00A01, \noptiflow \nfront",

"front Ellen" = "Doll\u00A02, \noptiflow \nfront",

"front To" = "Doll\u00A03, \noptiflow \nfront")) +

scale_shape_manual(

values = c("crv.x" = 16, "crv.y" = 17),

labels = c("crv.x" = "Nurses", "crv.y" = "Ground truth"),

name = "Measurement type") +

scale_color_manual(

values = c("crv.x" = "orange", "crv.y" = "black"),

labels = c("crv.x" = "Nurses", "crv.y" = "Ground truth"),

name = "Measurement type") +

guides(

color = guide_legend(

override.aes = list(

shape = c(16, 17),

color = c("orange", "black")),

title = "Measurement type",

labels = c("Nurses", "Ground truth")))+

scale_y_continuous (

limits = c(175, 350),

breaks = c(175, 200, 225, 250, 275, 300, 325, 350)

) +

theme(

panel.grid.minor = element_line(color = "lightgray", size = 0.1),

panel.grid.major = element_line(color = "lightgray", size = 0.1),

legend.box = "vertical"

)

# plot of percentiles

# Print de percentiel resultaten

print(percentiel_resultaten)

#Plotten van resultaten

# Zet de percentielen om in een data frame

percentiel_df <- do.call(rbind, lapply(names(percentiel_resultaten), function(name) {

data.frame(

test = name,

lower = percentiel_resultaten[[name]][1],

upper = percentiel_resultaten[[name]][2]

)

}))

# Maak de plot met ggplot2

ggplot(percentiel_df, aes(x = test, ymin = lower, ymax = upper)) +

geom_errorbar(width = 0.2) +

geom_point(aes(y = (lower + upper) / 2)) +

labs(title = "Percentielen van Deviaties", y = "Deviatie Percentage", x = "Test Conditie") +

theme(axis.text.x = element_text(angle = 45, hjust = 1))

# voor samenvoegen van errorbar en data

front_back <- data.frame (

optiflow_clear = c("front", "back", "front", "back", "front", "back", "front", "back"),

test = c("bl_beademingfront", "bl_beademingback", "crv_beademingfront", "crv_beademingback", "hcPS_beademingfront", "hcPS_beademingback", "hcHM_beademingfront","hcHM_beademingback"))

errorbar_test <- merge(percentiel_df, front_back, by = "test")

# plots deviation from ground truth, in percentage of ground truth, by doll -----------------

percentiel_df_pop <- do.call(rbind, lapply(names(percentiel_resultaten_pop), function(name) {

data.frame(

test = name,

lower = percentiel_resultaten_pop[[name]][1],

upper = percentiel_resultaten_pop[[name]][2]

)

}))

dolls <- data.frame (

doll = c("Ellen", "Ellen", "Ellen", "Ellen", "Ellen", "Ellen","Ellen", "Sophie", "Sophie", "Sophie", "Sophie", "Sophie", "Sophie", "Sophie", "To", "To", "To", "To", "To", "To", "To"),

test = c("bl_Ellen", "crv_beademingfrontEllen", "crv_beademingbackEllen", "hcPS_beademingfrontEllen", "hcPS_beademingbackEllen", "hcHM_beademingfrontEllen","hcHM_beademingbackEllen",

"bl_Sophie", "crv_beademingfrontSophie", "crv_beademingbackSophie", "hcPS_beademingfrontSophie", "hcPS_beademingbackSophie", "hcHM_beademingfrontSophie","hcHM_beademingbackSophie",

"bl_To", "crv_beademingfrontTo", "crv_beademingbackTo", "hcPS_beademingfrontTo", "hcPS_beademingbackTo", "hcHM_beademingfrontTo","hcHM_beademingbackTo"))

errorbar_test1 <- merge(percentiel_df_pop, dolls, by = "test")

# PLOTS PER DOLL, proportional deviation from GT-----------------------------------------------

# Body length

medianbl <- data.frame(

xmin = 0.5,

xmax = 1.5,

ymin = mean(Deviation_PS_Sophie$deviation_bl_percentage) - (bl_limit / groundtruthdata_Sophie$bl * 100),

ymax = mean(Deviation_PS_Sophie$deviation_bl_percentage) + (bl_limit / groundtruthdata_Sophie$bl * 100),

xmin1 = 1.5,

xmax1 = 2.5,

ymin1 = median(Deviation_PS_Ellen$deviation_bl_percentage) - (bl_limit / groundtruthdata_Ellen$bl * 100),

ymax1 = median(Deviation_PS_Ellen$deviation_bl_percentage) + (bl_limit / groundtruthdata_Ellen$bl * 100),

xmin2 = 2.5,

xmax2 = 3.5,

ymin2 = median(Deviation_PS_To$deviation_bl_percentage) - (bl_limit / groundtruthdata_To$bl * 100),

ymax2 = median(Deviation_PS_To$deviation_bl_percentage) + (bl_limit / groundtruthdata_To$bl * 100))

# Legend data for lines

legend_data <- data.frame(

x = c(-Inf, -Inf, -Inf),

xend = c(Inf, Inf, Inf),

y = c(NA, median(DeviationPS_alleen$deviation_bl_percentage), 0),

yend = c(NA, median(DeviationPS_alleen$deviation_bl_percentage), 0),

linetype = c("dotdash", "solid", "dotted"),

color = c("darkgrey", "red", "black"),

label = c("Median or \nmean per doll", "Median", "Ground truth")

)

medianbl$fill_dummy <- "Median Rect"

# Plot with specific legend for lines

DeviationPS_alleen$doll <- factor(DeviationPS_alleen$doll, levels = c("Sophie", "Ellen", "To"))

DeviationPS_alleen %>%

ggplot(aes(x = doll)) +

geom_segment(aes(x = -Inf, xend = Inf, y = median(deviation_bl_percentage), yend = median(deviation_bl_percentage), linetype = "Median"),

color = "red", size = 1) +

geom_segment(aes(x = -Inf, xend = Inf, y = 0, yend = 0, linetype = "Ground truth"),

color = "black", size = 1) +

geom_beeswarm(aes(y = deviation_bl_percentage, shape = doll, color = doll), cex =3, size=3) +

geom_rect(data = medianbl, aes(xmin = xmin, xmax = xmax, ymin = ymin, ymax = ymax, fill = fill_dummy),

inherit.aes = FALSE, alpha = 0.3) +

geom_rect(data = medianbl, aes(xmin = xmin1, xmax = xmax1, ymin = ymin1, ymax = ymax1),

inherit.aes = FALSE, fill = "grey", alpha = 0.3) +

geom_rect(data = medianbl, aes(xmin = xmin2, xmax = xmax2, ymin = ymin2, ymax = ymax2),

inherit.aes = FALSE, fill = "grey", alpha = 0.3) +

geom_segment(aes(x = 0.5, xend = 1.5, y = mean(Deviation_PS_Sophie$deviation_bl_percentage), yend = mean(Deviation_PS_Sophie$deviation_bl_percentage)),

linetype = "dotdash", color = "darkgrey", size = 1) +

geom_segment(aes(x = 1.5, xend = 2.5, y = median(Deviation_PS_Ellen$deviation_bl_percentage), yend = median(Deviation_PS_Ellen$deviation_bl_percentage)),

linetype = "dotdash", color = "darkgrey", size = 1) +

geom_segment(aes(x = 2.5, xend = 3.5, y = median(Deviation_PS_To$deviation_bl_percentage), yend = median(Deviation_PS_To$deviation_bl_percentage), linetype = "Median or \nmean per doll"),

color = "darkgrey", size = 1) +

geom_errorbar(data = errorbar_test1[c(2,1,3) ,],

aes(x = doll, ymin = lower, ymax = upper),

inherit.aes = FALSE,

width = 0.2,

position = position_dodge(width = 0.3)) +

theme_classic() +

scale_x_discrete(labels = c("Sophie" = "Doll 1", "Ellen" = "Doll 2", "To" = "Doll 3")) +

labs(

title = "Body Length PreemieScanner",

x = "Optiflow front and back",

y = "Deviation Body Length from ground truth (%)",

shape = "Dolls",

color = "Dolls",

linetype = "Lines"

) +

scale_shape_manual(

values = c("Sophie" = 16, "Ellen" = 8, "To" = 15),

labels = c("Doll 1", "Doll 2", "Doll 3"),

name = "Dolls"

) +

scale_color_manual(

values = c("Sophie" = 'chartreuse4', "Ellen" = "purple3", "To" = "darkorange"),

labels = c("Doll 1", "Doll 2", "Doll 3"),

name = "Dolls"

) +

scale_linetype_manual(

values = c("Median or \nmean per doll" = "dotdash", "Median" = "solid", "Ground truth" = "dotted"),

name = "Line type",

breaks = c("Median or \nmean per doll", "Median", "Ground truth"),

labels = c("Median or \nmean per doll", "Median", "Ground truth"),

guide = guide_legend(override.aes = list(fill = NA))

) +

scale_fill_manual (

values = c("Median Rect" = "grey"),

labels = c("Median Rect" = "Precision limits"),

name = NULL,

guide = guide_legend(override.aes = list(linetype = 0))

) +

guides(

fill = guide_legend(order = 2),

linetype = guide_legend(order = 1)

) +

scale_y_continuous (

limits = c(-10, 10),

breaks = c(-10, -8, -6, -4, -2, 0, 2, 4, 6, 8, 10)

) +

theme(

panel.grid.minor = element_line(color = "lightgray", size = 0.1),

panel.grid.major = element_line(color = "lightgray", size = 0.1),

legend.box = "vertical"

)

# PLOT HC OPTIFLOW FRONT

medianhcPS_beademingnaarvoren <- data.frame(

xmin = 0.5,

xmax = 1.5,

ymin = median(Deviation_PS_Sophie_beademingnaarvoren$deviation_hc_percentage) - (hc_limit / groundtruthdata_Sophie$hc * 100),

ymax = median(Deviation_PS_Sophie_beademingnaarvoren$deviation_hc_percentage) + (hc_limit / groundtruthdata_Sophie$hc * 100),

xmin1 = 1.5,

xmax1 = 2.5,

ymin1 = median(Deviation_PS_Ellen_beademingnaarvoren$deviation_hc_percentage) - (hc_limit / groundtruthdata_Ellen$hc * 100),

ymax1 = median(Deviation_PS_Ellen_beademingnaarvoren$deviation_hc_percentage) + (hc_limit / groundtruthdata_Ellen$hc * 100),

xmin2 = 2.5,

xmax2 = 3.5,

ymin2 = median(Deviation_PS_To_beademingnaarvoren$deviation_hc_percentage) - (hc_limit / groundtruthdata_To$hc * 100),

ymax2 = median(Deviation_PS_To_beademingnaarvoren$deviation_hc_percentage) + (hc_limit / groundtruthdata_To$hc * 100))

# Legend data for lines

legend_data <- data.frame(

x = c(-Inf, -Inf, -Inf),

xend = c(Inf, Inf, Inf),

y = c(NA, median(Deviation_PS_beademingnaarvoren$deviation_hc_percentage), 0),

yend = c(NA, median(Deviation_PS_beademingnaarvoren$deviation_hc_percentage), 0),

linetype = c("dotdash", "solid", "dotted"),

color = c("darkgrey", "red", "black"),

label = c("Median or \nmean per doll", "Median", "Ground truth")

)

medianhcPS_beademingnaarvoren$fill_dummy <- "Median Rect"

# Plot with specific legend for lines, beademing naar voren

Deviation_PS_beademingnaarvoren$doll <- factor(Deviation_PS_beademingnaarvoren$doll, levels = c("Sophie", "Ellen", "To"))

Deviation_PS_beademingnaarvoren %>%

ggplot(aes(x = doll)) +

geom_segment(aes(x = -Inf, xend = Inf, y = median(Deviation_PS_beademingnaarvoren$deviation_hc_percentage), yend = median(Deviation_PS_beademingnaarvoren$deviation_hc_percentage), linetype = "Median"),

color = "red", size = 1) +

geom_segment(aes(x = -Inf, xend = Inf, y = 0, yend = 0, linetype = "Ground truth"),

color = "black", size = 1) +

geom_beeswarm(aes(y = deviation_hc_percentage, shape = doll, color = doll), cex = 3, size=3) +

geom_rect(data = medianhcPS_beademingnaarvoren, aes(xmin = xmin, xmax = xmax, ymin = ymin, ymax = ymax, fill = fill_dummy),

inherit.aes = FALSE, alpha = 0.3) +

geom_rect(data = medianhcPS_beademingnaarvoren, aes(xmin = xmin1, xmax = xmax1, ymin = ymin1, ymax = ymax1),

inherit.aes = FALSE, fill = "grey", alpha = 0.3) +

geom_rect(data = medianhcPS_beademingnaarvoren, aes(xmin = xmin2, xmax = xmax2, ymin = ymin2, ymax = ymax2),

inherit.aes = FALSE, fill = "grey", alpha = 0.3) +

geom_segment(aes(x = 0.5, xend = 1.5, y = median(Deviation_PS_Sophie_beademingnaarvoren$deviation_hc_percentage), yend = median(Deviation_PS_Sophie_beademingnaarvoren$deviation_hc_percentage)),

linetype = "dotdash", color = "darkgrey", size = 1) +

geom_segment(aes(x = 1.5, xend = 2.5, y = median(Deviation_PS_Ellen_beademingnaarvoren$deviation_hc_percentage), yend = median(Deviation_PS_Ellen_beademingnaarvoren$deviation_hc_percentage)),

linetype = "dotdash", color = "darkgrey", size = 1) +

geom_segment(aes(x = 2.5, xend = 3.5, y = median(Deviation_PS_To_beademingnaarvoren$deviation_hc_percentage), yend = median(Deviation_PS_To_beademingnaarvoren$deviation_hc_percentage), linetype = "Median per doll"),

color = "darkgrey", size = 1) +

geom_errorbar(data = errorbar_test1[c(20,19,21) ,],

aes(x = doll, ymin = lower, ymax = upper),

inherit.aes = FALSE,

width = 0.2,

position = position_dodge(width = 0.3)) +

theme_classic() +

scale_x_discrete(labels = c("Sophie" = "Doll 1",

"Ellen" = "Doll 2",

"To" = "Doll 3")) +

labs(

title = "Head Circumference PreemieScanner",

x = "Optiflow front",

y = "Deviation Head Circumference from ground truth (%)",

shape = "Dolls",

color = "Dolls",

linetype = "Lines"

) +

scale_shape_manual(

values = c("Sophie" = 16, "Ellen" = 8, "To" = 15),

labels = c("Doll 1", "Doll 2", "Doll 3"),

name = "Dolls"

) +

scale_color_manual(

values = c("Sophie" = 'chartreuse4', "Ellen" = "purple3", "To" = "darkorange"),

labels = c("Doll 1", "Doll 2", "Doll 3"),

name = "Dolls"

) +

scale_linetype_manual(

values = c("Median per doll" = "dotdash", "Median" = "solid", "Ground truth" = "dotted"),

name = "Line type",

breaks = c("Median per doll", "Median", "Ground truth"),

labels = c("Median per doll", "Median", "Ground truth"),

guide = guide_legend(override.aes = list(fill = NA))

) +

scale_fill_manual (

values = c("Median Rect" = "grey"),

labels = c("Median Rect" = "Precision limits"),

name = NULL,

guide = guide_legend(override.aes = list(linetype = 0))

) +

guides(

fill = guide_legend(order = 2),

linetype = guide_legend(order = 1)

) +

scale_y_continuous (

limits = c(-10, 10),

breaks = c(-10, -8, -6, -4, -2, 0, 2, 4, 6, 8, 10)

) +

theme(

panel.grid.minor = element_line(color = "lightgray", size = 0.1),

panel.grid.major = element_line(color = "lightgray", size = 0.1),

legend.box = "vertical"

)

# optiflow achterlangs

medianhcPS_beademingnaarachter<- data.frame(

xmin = 0.5,

xmax = 1.5,

ymin = median(Deviation_PS_Sophie_beademingnaarachter$deviation_hc_percentage) - (hc_limit / groundtruthdata_Sophie$hc * 100),

ymax = median(Deviation_PS_Sophie_beademingnaarachter$deviation_hc_percentage) + (hc_limit / groundtruthdata_Sophie$hc * 100),

xmin1 = 1.5,

xmax1 = 2.5,

ymin1 = median(Deviation_PS_Ellen_beademingnaarachter$deviation_hc_percentage, na.rm = TRUE) - (hc_limit / groundtruthdata_Ellen$hc * 100),

ymax1 = median(Deviation_PS_Ellen_beademingnaarachter$deviation_hc_percentage, na.rm = TRUE) + (hc_limit / groundtruthdata_Ellen$hc * 100),

xmin2 = 2.5,

xmax2 = 3.5,

ymin2 = median(Deviation_PS_To_beademingnaarachter$deviation_hc_percentage) - (hc_limit / groundtruthdata_To$hc * 100),

ymax2 = median(Deviation_PS_To_beademingnaarachter$deviation_hc_percentage) + (hc_limit / groundtruthdata_To$hc * 100))

# Legend data for lines

legend_data <- data.frame(

x = c(-Inf, -Inf, -Inf),

xend = c(Inf, Inf, Inf),

y = c(NA, median(Deviation_PS_beademingnaarachter$deviation_hc_percentage, na.rm = TRUE), 0),

yend = c(NA, median(Deviation_PS_beademingnaarachter$deviation_hc_percentage, na.rm = TRUE), 0),

linetype = c("dotdash", "solid", "dotted"),

color = c("darkgrey", "red", "black"),

label = c("Median or \nmean per doll", "Median", "Ground truth")

)

medianhcPS_beademingnaarachter$fill_dummy <- "Median Rect"

# Plot with specific legend for lines, beademing naar voren

Deviation_PS_beademingnaarachter$doll <- factor(Deviation_PS_beademingnaarachter$doll, levels = c("Sophie", "Ellen", "To"))

Deviation_PS_beademingnaarachter %>%

ggplot(aes(x = doll)) +

geom_segment(aes(x = -Inf, xend = Inf, y = median(deviation_hc_percentage, na.rm = TRUE), yend = median(deviation_hc_percentage, na.rm = TRUE), linetype = "Median"),

color = "red", size = 1) +

geom_segment(aes(x = -Inf, xend = Inf, y = 0, yend = 0, linetype = "Ground truth"),

color = "black", size = 1) +

geom_beeswarm(aes(y = deviation_hc_percentage, shape = doll, color = doll), cex = 3, size=3) +

geom_rect(data = medianhcPS_beademingnaarachter, aes(xmin = xmin, xmax = xmax, ymin = ymin, ymax = ymax, fill = fill_dummy),

inherit.aes = FALSE, alpha = 0.3) +

geom_rect(data = medianhcPS_beademingnaarachter, aes(xmin = xmin1, xmax = xmax1, ymin = ymin1, ymax = ymax1),

inherit.aes = FALSE, fill = "grey", alpha = 0.3) +

geom_rect(data = medianhcPS_beademingnaarachter, aes(xmin = xmin2, xmax = xmax2, ymin = ymin2, ymax = ymax2),

inherit.aes = FALSE, fill = "grey", alpha = 0.3) +

geom_segment(aes(x = 0.5, xend = 1.5, y = median(Deviation_PS_Sophie_beademingnaarachter$deviation_hc_percentage), yend = median(Deviation_PS_Sophie_beademingnaarachter$deviation_hc_percentage)),

linetype = "dotdash", color = "darkgrey", size = 1) +

geom_segment(aes(x = 1.5, xend = 2.5, y = median(Deviation_PS_Ellen_beademingnaarachter$deviation_hc_percentage, na.rm = TRUE), yend = median(Deviation_PS_Ellen_beademingnaarachter$deviation_hc_percentage, na.rm = TRUE)),

linetype = "dotdash", color = "darkgrey", size = 1) +

geom_segment(aes(x = 2.5, xend = 3.5, y = median(Deviation_PS_To_beademingnaarachter$deviation_hc_percentage), yend = median(Deviation_PS_To_beademingnaarachter$deviation_hc_percentage), linetype = "Median per doll"),

color = "darkgrey", size = 1) +

geom_errorbar(data = errorbar_test1[c(17,16,18) ,],

aes(x = doll, ymin = lower, ymax = upper),

inherit.aes = FALSE,

width = 0.2,

position = position_dodge(width = 0.3)) +

theme_classic() +

scale_x_discrete(labels = c("Sophie" = "Doll 1",

"Ellen" = "Doll 2",

"To" = "Doll 3")) +

labs(

title = "Head Circumference PreemieScanner",

x = "Optiflow back",

y = "Deviation Head Circumference from ground truth (%)",

shape = "Dolls",

color = "Dolls",

linetype = "Lines"

) +

scale_shape_manual(

values = c("Sophie" = 16, "Ellen" = 8, "To" = 15),

labels = c("Doll 1", "Doll 2", "Doll 3"),

name = "Dolls"

) +

scale_color_manual(

values = c("Sophie" = 'chartreuse4', "Ellen" = "purple3", "To" = "darkorange"),

labels = c("Doll 1", "Doll 2", "Doll 3"),

name = "Dolls"

) +

scale_linetype_manual(

values = c("Median per doll" = "dotdash", "Median" = "solid", "Ground truth" = "dotted"),

name = "Line type",

breaks = c("Median per doll", "Median", "Ground truth"),

labels = c("Median per doll", "Median", "Ground truth"),

guide = guide_legend(override.aes = list(fill = NA))

) +

scale_fill_manual (

values = c("Median Rect" = "grey"),

labels = c("Median Rect" = "Precision limits"),

name = NULL,

guide = guide_legend(override.aes = list(linetype = 0))

) +

guides(

fill = guide_legend(order = 2),

linetype = guide_legend(order = 1)

) +

scale_y_continuous (

limits = c(-10, 10),

breaks = c(-10, -8, -6, -4, -2, 0, 2, 4, 6, 8, 10)

) +

theme(

panel.grid.minor = element_line(color = "lightgray", size = 0.1),

panel.grid.major = element_line(color = "lightgray", size = 0.1),

legend.box = "vertical"

)

# Plot HC Measuring tape by doll ------------

# optiflow front

medianhcHM_beademingnaarvoren <- data.frame(

xmin = 0.5,

xmax = 1.5,

ymin = median(Deviation_HM_Sophie_beademingnaarvoren$deviation_hc_percentage) - (hc_limit / groundtruthdata_Sophie$hc * 100),

ymax = median(Deviation_HM_Sophie_beademingnaarvoren$deviation_hc_percentage) + (hc_limit / groundtruthdata_Sophie$hc * 100),

xmin1 = 1.5,

xmax1 = 2.5,

ymin1 = mean(Deviation_HM_Ellen_beademingnaarvoren$deviation_hc_percentage) - (hc_limit / groundtruthdata_Ellen$hc * 100),

ymax1 = mean(Deviation_HM_Ellen_beademingnaarvoren$deviation_hc_percentage) + (hc_limit / groundtruthdata_Ellen$hc * 100),

xmin2 = 2.5,

xmax2 = 3.5,

ymin2 = median(Deviation_HM_To_beademingnaarvoren$deviation_hc_percentage) - (hc_limit / groundtruthdata_To$hc * 100),

ymax2 = median(Deviation_HM_To_beademingnaarvoren$deviation_hc_percentage) + (hc_limit / groundtruthdata_To$hc * 100))

# Legend data for lines

legend_data <- data.frame(

x = c(-Inf, -Inf, -Inf),

xend = c(Inf, Inf, Inf),

y = c(NA, median(Deviation_HM_beademingnaarvoren$deviation_hc_percentage), 0),

yend = c(NA, median(Deviation_HM_beademingnaarvoren$deviation_hc_percentage), 0),

linetype = c("dotdash", "solid", "dotted"),

color = c("darkgrey", "red", "black"),

label = c("Median or \nmean per doll", "Median", "Ground truth")

)

medianhcHM_beademingnaarvoren$fill_dummy <- "Median Rect"

# Plot with specific legend for lines, beademing naar voren

Deviation_HM_beademingnaarvoren$doll <- factor(Deviation_HM_beademingnaarvoren$doll, levels = c("Sophie", "Ellen", "To"))

Deviation_HM_beademingnaarvoren %>%

ggplot(aes(x = doll)) +

geom_segment(aes(x = -Inf, xend = Inf, y = median(Deviation_HM_beademingnaarvoren$deviation_hc_percentage), yend = median(Deviation_HM_beademingnaarvoren$deviation_hc_percentage), linetype = "Median"),

color = "red", size = 1) +

geom_segment(aes(x = -Inf, xend = Inf, y = 0, yend = 0, linetype = "Ground truth"),

color = "black", size = 1) +

geom_beeswarm(aes(y = deviation_hc_percentage, shape = doll, color = doll), cex = 3, size=3) +

geom_rect(data = medianhcHM_beademingnaarvoren, aes(xmin = xmin, xmax = xmax, ymin = ymin, ymax = ymax, fill = fill_dummy),

inherit.aes = FALSE, alpha = 0.3) +

geom_rect(data = medianhcHM_beademingnaarvoren, aes(xmin = xmin1, xmax = xmax1, ymin = ymin1, ymax = ymax1),

inherit.aes = FALSE, fill = "grey", alpha = 0.3) +

geom_rect(data = medianhcHM_beademingnaarvoren, aes(xmin = xmin2, xmax = xmax2, ymin = ymin2, ymax = ymax2),

inherit.aes = FALSE, fill = "grey", alpha = 0.3) +

geom_segment(aes(x = 0.5, xend = 1.5, y = median(Deviation_HM_Sophie_beademingnaarvoren$deviation_hc_percentage), yend = median(Deviation_HM_Sophie_beademingnaarvoren$deviation_hc_percentage)),

linetype = "dotdash", color = "darkgrey", size = 1) +

geom_segment(aes(x = 1.5, xend = 2.5, y = mean(Deviation_HM_Ellen_beademingnaarvoren$deviation_hc_percentage), yend = mean(Deviation_HM_Ellen_beademingnaarvoren$deviation_hc_percentage)),

linetype = "dotdash", color = "darkgrey", size = 1) +

geom_segment(aes(x = 2.5, xend = 3.5, y = median(Deviation_HM_To_beademingnaarvoren$deviation_hc_percentage), yend = median(Deviation_HM_To_beademingnaarvoren$deviation_hc_percentage), linetype = "Median or \nmean per doll"),

color = "darkgrey", size = 1) +

geom_errorbar(data = errorbar_test1[c(14,13,15) ,],

aes(x = doll, ymin = lower, ymax = upper),

inherit.aes = FALSE,

width = 0.2,

position = position_dodge(width = 0.3)) +

theme_classic() +

scale_x_discrete(labels = c("Sophie" = "Doll 1",

"Ellen" = "Doll 2",

"To" = "Doll 3")) +

labs(

title = "Head Circumference Measuring Tape",

x = "Optiflow front",

y = "Deviation Head Circumference (Tape) from ground truth (%)",

shape = "Dolls",

color = "Dolls",

linetype = "Lines"

) +

scale_shape_manual(

values = c("Sophie" = 16, "Ellen" = 8, "To" = 15),

labels = c("Doll 1", "Doll 2", "Doll 3"),

name = "Dolls"

) +

scale_color_manual(

values = c("Sophie" = 'chartreuse4', "Ellen" = "purple3", "To" = "darkorange"),

labels = c("Doll 1", "Doll 2", "Doll 3"),

name = "Dolls"

) +

scale_linetype_manual(

values = c("Median or \nmean per doll" = "dotdash", "Median" = "solid", "Ground truth" = "dotted"),

name = "Lijnen",

breaks = c("Median or \nmean per doll", "Median", "Ground truth"),

labels = c("Median or \nmean per doll", "Median", "Ground truth"),

guide = guide_legend(override.aes = list(fill = NA))

) +

scale_fill_manual (

values = c("Median Rect" = "grey"),

labels = c("Median Rect" = "Precision limits"),

name = NULL,

guide = guide_legend(override.aes = list(linetype = 0))

) +

guides(

fill = guide_legend(order = 2),

linetype = guide_legend(order = 1)

) +

scale_y_continuous (

limits = c(-10, 10),

breaks = c(-10, -8, -6, -4, -2, 0, 2, 4, 6, 8, 10)

) +

theme(

panel.grid.minor = element_line(color = "lightgray", size = 0.1),

panel.grid.major = element_line(color = "lightgray", size = 0.1),

legend.box = "vertical"

)

# optiflow achterlangs

medianhcHM_beademingnaarachter<- data.frame(

xmin = 0.5,

xmax = 1.5,

ymin = mean(Deviation_HM_Sophie_beademingnaarachter$deviation_hc_percentage) - (hc_limit / groundtruthdata_Sophie$hc * 100),

ymax = mean(Deviation_HM_Sophie_beademingnaarachter$deviation_hc_percentage) + (hc_limit / groundtruthdata_Sophie$hc * 100),

xmin1 = 1.5,

xmax1 = 2.5,

ymin1 = mean(Deviation_HM_Ellen_beademingnaarachter$deviation_hc_percentage, na.rm = TRUE) - (hc_limit / groundtruthdata_Ellen$hc * 100),

ymax1 = mean(Deviation_HM_Ellen_beademingnaarachter$deviation_hc_percentage, na.rm = TRUE) + (hc_limit / groundtruthdata_Ellen$hc * 100),

xmin2 = 2.5,

xmax2 = 3.5,

ymin2 = median(Deviation_HM_To_beademingnaarachter$deviation_hc_percentage) - (hc_limit / groundtruthdata_To$hc * 100),

ymax2 = median(Deviation_HM_To_beademingnaarachter$deviation_hc_percentage) + (hc_limit / groundtruthdata_To$hc * 100))

# Legend data for lines

legend_data <- data.frame(

x = c(-Inf, -Inf, -Inf),

xend = c(Inf, Inf, Inf),

y = c(NA, median(Deviation_HM_beademingnaarachter$deviation_hc_percentage, na.rm = TRUE), 0),

yend = c(NA, median(Deviation_HM_beademingnaarachter$deviation_hc_percentage, na.rm = TRUE), 0),

linetype = c("dotdash", "solid", "dotted"),

color = c("darkgrey", "red", "black"),

label = c("Median or \nmean per doll", "Median", "Ground truth")

)

medianhcHM_beademingnaarachter$fill_dummy <- "Median Rect"

# Plot with specific legend for lines, beademing naar voren

Deviation_HM_beademingnaarachter$doll <- factor(Deviation_HM_beademingnaarachter$doll, levels = c("Sophie", "Ellen", "To"))

Deviation_HM_beademingnaarachter %>%

ggplot(aes(x = doll)) +

geom_segment(aes(x = -Inf, xend = Inf, y = median(deviation_hc_percentage, na.rm = TRUE), yend = median(deviation_hc_percentage, na.rm = TRUE), linetype = "Median"),

color = "red", size = 1) +

geom_segment(aes(x = -Inf, xend = Inf, y = 0, yend = 0, linetype = "Ground truth"),

color = "black", size = 1) +

geom_beeswarm(aes(y = deviation_hc_percentage, shape = doll, color = doll), cex = 3, size=3) +

geom_rect(data = medianhcHM_beademingnaarachter, aes(xmin = xmin, xmax = xmax, ymin = ymin, ymax = ymax, fill = fill_dummy),

inherit.aes = FALSE, alpha = 0.3) +

geom_rect(data = medianhcHM_beademingnaarachter, aes(xmin = xmin1, xmax = xmax1, ymin = ymin1, ymax = ymax1),

inherit.aes = FALSE, fill = "grey", alpha = 0.3) +

geom_rect(data = medianhcHM_beademingnaarachter, aes(xmin = xmin2, xmax = xmax2, ymin = ymin2, ymax = ymax2),

inherit.aes = FALSE, fill = "grey", alpha = 0.3) +

geom_segment(aes(x = 0.5, xend = 1.5, y = mean(Deviation_HM_Sophie_beademingnaarachter$deviation_hc_percentage), yend = mean(Deviation_HM_Sophie_beademingnaarachter$deviation_hc_percentage)),

linetype = "dotdash", color = "darkgrey", size = 1) +

geom_segment(aes(x = 1.5, xend = 2.5, y = mean(Deviation_HM_Ellen_beademingnaarachter$deviation_hc_percentage, na.rm = TRUE), yend = mean(Deviation_HM_Ellen_beademingnaarachter$deviation_hc_percentage, na.rm = TRUE)),

linetype = "dotdash", color = "darkgrey", size = 1) +

geom_segment(aes(x = 2.5, xend = 3.5, y = median(Deviation_HM_To_beademingnaarachter$deviation_hc_percentage), yend = median(Deviation_HM_To_beademingnaarachter$deviation_hc_percentage), linetype = "Median or \nmean per doll"),

color = "darkgrey", size = 1) +

geom_errorbar(data = errorbar_test1[c(11,10,12) ,],

aes(x = doll, ymin = lower, ymax = upper),

inherit.aes = FALSE,

width = 0.2,

position = position_dodge(width = 0.3)) +

theme_classic() +

scale_x_discrete(labels = c("Sophie" = "Doll 1",

"Ellen" = "Doll 2",

"To" = "Doll 3")) +

labs(

title = "Head Circumference Measuring Tape",

x = "Optiflow back",

y = "Deviation Head Circumference (Tape) from ground truth (%)",

shape = "Dolls",

color = "Dolls",

linetype = "Lines"

) +

scale_shape_manual(

values = c("Sophie" = 16, "Ellen" = 8, "To" = 15),

labels = c("Doll 1", "Doll 2", "Doll 3"),

name = "Dolls"

) +

scale_color_manual(

values = c("Sophie" = 'chartreuse4', "Ellen" = "purple3", "To" = "darkorange"),

labels = c("Doll 1", "Doll 2", "Doll 3"),

name = "Dolls"

) +

scale_linetype_manual(

values = c("Median or \nmean per doll" = "dotdash", "Median" = "solid", "Ground truth" = "dotted"),

name = "Line type",

breaks = c("Median or \nmean per doll", "Median", "Ground truth"),

labels = c("Median or \nmean per doll", "Median", "Ground truth"),

guide = guide_legend(override.aes = list(fill = NA))

) +

scale_fill_manual (

values = c("Median Rect" = "grey"),

labels = c("Median Rect" = "Precision limits"),

name = NULL,

guide = guide_legend(override.aes = list(linetype = 0))

) +

guides(

fill = guide_legend(order = 2),

linetype = guide_legend(order = 1)

) +

scale_y_continuous (

limits = c(-10, 10),

breaks = c(-10, -8, -6, -4, -2, 0, 2, 4, 6, 8, 10)

) +

theme(

panel.grid.minor = element_line(color = "lightgray", size = 0.1),

panel.grid.major = element_line(color = "lightgray", size = 0.1),

legend.box = "vertical"

)

# crv plot per doll ------------

# crv optiflow voorlangs

mediancrvPS_beademingnaarvoren <- data.frame(

xmin = 0.5,

xmax = 1.5,

ymin = mean(Deviation_PS_Sophie_beademingnaarvoren$deviation_crv_percentage, na.rm = TRUE) - (crv_limit / groundtruthdata_Sophie$crv * 100),

ymax = mean(Deviation_PS_Sophie_beademingnaarvoren$deviation_crv_percentage, na.rm = TRUE) + (crv_limit / groundtruthdata_Sophie$crv * 100),

xmin1 = 1.5,

xmax1 = 2.5,

ymin1 = mean(Deviation_PS_Ellen_beademingnaarvoren$deviation_crv_percentage, na.rm = TRUE) - (crv_limit / groundtruthdata_Ellen$crv * 100),

ymax1 = mean(Deviation_PS_Ellen_beademingnaarvoren$deviation_crv_percentage, na.rm = TRUE) + (crv_limit / groundtruthdata_Ellen$crv * 100),

xmin2 = 2.5,

xmax2 = 3.5,

ymin2 = median(Deviation_PS_To_beademingnaarvoren$deviation_crv_percentage) - (crv_limit / groundtruthdata_To$crv * 100),

ymax2 = median(Deviation_PS_To_beademingnaarvoren$deviation_crv_percentage) + (crv_limit / groundtruthdata_To$crv * 100))

# Legend data for lines

legend_data <- data.frame(

x = c(-Inf, -Inf, -Inf),

xend = c(Inf, Inf, Inf),

y = c(NA, median(Deviation_PS_beademingnaarvoren$deviation_crv_percentage, na.rm = TRUE), 0),

yend = c(NA, median(Deviation_PS_beademingnaarvoren$deviation_crv_percentage, na.rm = TRUE), 0),

linetype = c("dotdash", "solid", "dotted"),

color = c("darkgrey", "red", "black"),

label = c("Median or \nmean per doll", "Median", "Ground truth")

)

mediancrvPS_beademingnaarvoren$fill_dummy <- "Median Rect"

# Plot with specific legend for lines, beademing naar voren

Deviation_PS_beademingnaarvoren$doll <- factor(Deviation_PS_beademingnaarvoren$doll, levels = c("Sophie", "Ellen", "To"))

Deviation_PS_beademingnaarvoren %>%

ggplot(aes(x = doll)) +

geom_segment(aes(x = -Inf, xend = Inf, y = median(deviation_crv_percentage, na.rm = TRUE), yend = median(deviation_crv_percentage, na.rm = TRUE), linetype = "Median"),

color = "red", size = 1) +

geom_segment(aes(x = -Inf, xend = Inf, y = 0, yend = 0, linetype = "Ground truth"),

color = "black", size = 1) +

geom_beeswarm(aes(y = deviation_crv_percentage, shape = doll, color = doll), cex = 3, size=3) +

geom_rect(data = mediancrvPS_beademingnaarvoren, aes(xmin = xmin, xmax = xmax, ymin = ymin, ymax = ymax, fill = fill_dummy),

inherit.aes = FALSE, alpha = 0.3) +

geom_rect(data = mediancrvPS_beademingnaarvoren, aes(xmin = xmin1, xmax = xmax1, ymin = ymin1, ymax = ymax1),

inherit.aes = FALSE, fill = "grey", alpha = 0.3) +

geom_rect(data = mediancrvPS_beademingnaarvoren, aes(xmin = xmin2, xmax = xmax2, ymin = ymin2, ymax = ymax2),

inherit.aes = FALSE, fill = "grey", alpha = 0.3) +

geom_segment(aes(x = 0.5, xend = 1.5, y = mean(Deviation_PS_Sophie_beademingnaarvoren$deviation_crv_percentage, na.rm = TRUE), yend = mean(Deviation_PS_Sophie_beademingnaarvoren$deviation_crv_percentage, na.rm = TRUE)),

linetype = "dotdash", color = "darkgrey", size = 1) +

geom_segment(aes(x = 1.5, xend = 2.5, y = mean(Deviation_PS_Ellen_beademingnaarvoren$deviation_crv_percentage, na.rm = TRUE), yend = mean(Deviation_PS_Ellen_beademingnaarvoren$deviation_crv_percentage, na.rm = TRUE)),

linetype = "dotdash", color = "darkgrey", size = 1) +

geom_segment(aes(x = 2.5, xend = 3.5, y = median(Deviation_PS_To_beademingnaarvoren$deviation_crv_percentage, na.rm = TRUE), yend = median(Deviation_PS_To_beademingnaarvoren$deviation_crv_percentage, na.rm = TRUE), linetype = "Median or \nmean per doll"),

color = "darkgrey", size = 1) +

geom_errorbar(data = errorbar_test1[c(8,7,9) ,],

aes(x = doll, ymin = lower, ymax = upper),

inherit.aes = FALSE,

width = 0.2,

position = position_dodge(width = 0.3)) +

theme_classic() +

scale_x_discrete(labels = c("Sophie" = "Doll 1",

"Ellen" = "Doll 2",

"To" = "Doll 3")) +

labs(

title = "Cranial Volume PreemieScanner",

x = "Optiflow front",

y = "Deviation Cranial Volume from ground truth (%)",

shape = "Dolls",

color = "Dolls",

linetype = "Lines"

) +

scale_shape_manual(

values = c("Sophie" = 16, "Ellen" = 8, "To" = 15),

labels = c("Doll 1", "Doll 2", "Doll 3"),

name = "Dolls"

) +

scale_color_manual(

values = c("Sophie" = 'chartreuse4', "Ellen" = "purple3", "To" = "darkorange"),

labels = c("Doll 1", "Doll 2", "Doll 3"),

name = "Dolls"

) +

scale_linetype_manual(

values = c("Median or \nmean per doll" = "dotdash", "Median" = "solid", "Ground truth" = "dotted"),

name = "Line type",

breaks = c("Median or \nmean per doll", "Median", "Ground truth"),

labels = c("Median or \nmean per doll", "Median", "Ground truth"),

guide = guide_legend(override.aes = list(fill = NA))

) +

scale_fill_manual (

values = c("Median Rect" = "grey"),

labels = c("Median Rect" = "Precision limits"),

name = NULL,

guide = guide_legend(override.aes = list(linetype = 0))

) +

guides(

fill = guide_legend(order = 2),

linetype = guide_legend(order = 1)

) +

scale_y_continuous (

limits = c(-10, 10),

breaks = c(-10, -8, -6, -4, -2, 0, 2, 4, 6, 8, 10)

) +

theme(

panel.grid.minor = element_line(color = "lightgray", size = 0.1),

panel.grid.major = element_line(color = "lightgray", size = 0.1),

legend.box = "vertical"

)

# crv optiflow back

mediancrvPS_beademingnaarachter<- data.frame(

xmin = 0.5,

xmax = 1.5,

ymin = mean(Deviation_PS_Sophie_beademingnaarachter$deviation_crv_percentage, na.rm = TRUE) - (crv_limit / groundtruthdata_Sophie$crv * 100),

ymax = mean(Deviation_PS_Sophie_beademingnaarachter$deviation_crv_percentage, na.rm = TRUE) + (crv_limit / groundtruthdata_Sophie$crv * 100),

xmin1 = 1.5,

xmax1 = 2.5,

ymin1 = mean(Deviation_PS_Ellen_beademingnaarachter$deviation_crv_percentage, na.rm = TRUE) - (crv_limit / groundtruthdata_Ellen$crv * 100),

ymax1 = mean(Deviation_PS_Ellen_beademingnaarachter$deviation_crv_percentage, na.rm = TRUE) + (crv_limit / groundtruthdata_Ellen$crv * 100),

xmin2 = 2.5,

xmax2 = 3.5,

ymin2 = median(Deviation_PS_To_beademingnaarachter$deviation_crv_percentage) - (crv_limit / groundtruthdata_To$crv * 100),

ymax2 = median(Deviation_PS_To_beademingnaarachter$deviation_crv_percentage) + (crv_limit / groundtruthdata_To$crv * 100))

# Legend data for lines

legend_data <- data.frame(

x = c(-Inf, -Inf, -Inf),

xend = c(Inf, Inf, Inf),

y = c(NA, mean(Deviation_PS_beademingnaarachter$deviation_crv_percentage, na.rm = TRUE), 0),

yend = c(NA, mean(Deviation_PS_beademingnaarachter$deviation_crv_percentage, na.rm = TRUE), 0),

linetype = c("dotdash", "solid", "dotted"),

color = c("darkgrey", "red", "black"),

label = c("Median or \nmean per doll", "Median or \nmean", "Ground truth")

)

mediancrvPS_beademingnaarachter$fill_dummy <- "Median Rect"

# Plot with specific legend for lines, crv optiflow back

Deviation_PS_beademingnaarachter$doll <- factor(Deviation_PS_beademingnaarachter$doll, levels = c("Sophie", "Ellen", "To"))

Deviation_PS_beademingnaarachter %>%

ggplot(aes(x = doll)) +

geom_segment(aes(x = -Inf, xend = Inf, y = mean(deviation_crv_percentage, na.rm = TRUE), yend = mean(deviation_crv_percentage, na.rm = TRUE), linetype = "Median or \nmean"),

color = "red", size = 1) +

geom_segment(aes(x = -Inf, xend = Inf, y = 0, yend = 0, linetype = "Ground truth"),

color = "black", size = 1) +

geom_beeswarm(aes(y = deviation_crv_percentage, shape = doll, color = doll), cex = 3, size=3) +

geom_rect(data = mediancrvPS_beademingnaarachter, aes(xmin = xmin, xmax = xmax, ymin = ymin, ymax = ymax, fill = fill_dummy),

inherit.aes = FALSE, alpha = 0.3) +

geom_rect(data = mediancrvPS_beademingnaarachter, aes(xmin = xmin1, xmax = xmax1, ymin = ymin1, ymax = ymax1),

inherit.aes = FALSE, fill = "grey", alpha = 0.3) +

geom_rect(data = mediancrvPS_beademingnaarachter, aes(xmin = xmin2, xmax = xmax2, ymin = ymin2, ymax = ymax2),

inherit.aes = FALSE, fill = "grey", alpha = 0.3) +

geom_segment(aes(x = 0.5, xend = 1.5, y = mean(Deviation_PS_Sophie_beademingnaarachter$deviation_crv_percentage, na.rm = TRUE), yend = mean(Deviation_PS_Sophie_beademingnaarachter$deviation_crv_percentage, na.rm = TRUE)),

linetype = "dotdash", color = "darkgrey", size = 1) +

geom_segment(aes(x = 1.5, xend = 2.5, y = mean(Deviation_PS_Ellen_beademingnaarachter$deviation_crv_percentage, na.rm = TRUE), yend = mean(Deviation_PS_Ellen_beademingnaarachter$deviation_crv_percentage, na.rm = TRUE)),

linetype = "dotdash", color = "darkgrey", size = 1) +

geom_segment(aes(x = 2.5, xend = 3.5, y = median(Deviation_PS_To_beademingnaarachter$deviation_crv_percentage), yend = median(Deviation_PS_To_beademingnaarachter$deviation_crv_percentage), linetype = "Median or \nmean per doll"),

color = "darkgrey", size = 1) +

geom_errorbar(data = errorbar_test1[c(5,4,6) ,],

aes(x = doll, ymin = lower, ymax = upper),

inherit.aes = FALSE,

width = 0.2,

position = position_dodge(width = 0.3)) +

theme_classic() +

scale_x_discrete(labels = c("Sophie" = "Doll 1",

"Ellen" = "Doll 2",

"To" = "Doll 3")) +

labs(

title = "Cranial Volume PreemieScanner",

x = "Optiflow back",

y = "Deviation Cranial Volume from ground truth (%)",

shape = "Dolls",

color = "Dolls",

linetype = "Lines"

) +

scale_shape_manual(

values = c("Sophie" = 16, "Ellen" = 8, "To" = 15),

labels = c("Doll 1", "Doll 2", "Doll 3"),

name = "Dolls"

) +

scale_color_manual(

values = c("Sophie" = 'chartreuse4', "Ellen" = "purple3", "To" = "darkorange"),

labels = c("Doll 1", "Doll 2", "Doll 3"),

name = "Dolls"

) +

scale_linetype_manual(

values = c("Median or \nmean per doll" = "dotdash", "Median or \nmean" = "solid", "Ground truth" = "dotted"),

name = "Line type",

breaks = c("Median or \nmean per doll", "Median or \nmean", "Ground truth"),

labels = c("Median or \nmean per doll", "Median or \nmean", "Ground truth"),

guide = guide_legend(override.aes = list(fill = NA))

) +

scale_fill_manual (

values = c("Median Rect" = "grey"),

labels = c("Median Rect" = "Precision limits"),

name = NULL,

guide = guide_legend(override.aes = list(linetype = 0))

) +

guides(

fill = guide_legend(order = 2),

linetype = guide_legend(order = 1)

) +

scale_y_continuous (

limits = c(-10, 10),

breaks = c(-10, -8, -6, -4, -2, 0, 2, 4, 6, 8, 10)

) +

theme(

panel.grid.minor = element_line(color = "lightgray", size = 0.1),

panel.grid.major = element_line(color = "lightgray", size = 0.1),

legend.box = "vertical"

)

# how many within border of precision, nu afwijking, maar eigenlijk willen we nauwkeurigheid op 0 hebben om dit te berekenen -------------

border_data_PS_beademingfront <- Deviation_PS_beademingnaarvoren %>%

mutate(withinborder_bl = deviation_bl >= -bl_limit & deviation_bl <= bl_limit, na.rm = TRUE) %>%

mutate(withinborder_hcPS = deviation_hc>= -hc_limit & deviation_hc <= hc_limit, na.rm = TRUE) %>%

mutate(withinborder_crv = deviation_crv>= -crv_limit & deviation_crv <= crv_limit, na.rm = TRUE)

border_data_PS_beademingback <- Deviation_PS_beademingnaarachter %>%

mutate(withinborder_bl = deviation_bl >= -bl_limit & deviation_bl <= bl_limit, na.rm = TRUE) %>%

mutate(withinborder_hcPS = deviation_hc>= -hc_limit & deviation_hc <= hc_limit, na.rm = TRUE) %>%

mutate(withinborder_crv = deviation_crv>= -crv_limit & deviation_crv <= crv_limit, na.rm = TRUE)

border_data_HM_beademingfront <- Deviation_HM_beademingnaarvoren %>%

mutate(withinborder_hcHM = deviation_hc >= -hc_limit & deviation_hc <= hc_limit, na.rm = TRUE)

border_data_HM_beademingback <- Deviation_HM_beademingnaarachter %>%

mutate(withinborder_hcHM = deviation_hc >= -hc_limit & deviation_hc <= hc_limit, na.rm = TRUE)

count_bl_beademingfront <- sum(border_data_PS_beademingfront$withinborder_bl, na.rm = TRUE)

count_bl_beademingback <- sum(border_data_PS_beademingback$withinborder_bl, na.rm = TRUE)

count_hcPS_beademingfront <- sum(border_data_PS_beademingfront$withinborder_hcPS, na.rm = TRUE)

count_hcPS_beademingback <- sum(border_data_PS_beademingback$withinborder_hcPS, na.rm = TRUE)

count_crv_beademingfront <- sum(border_data_PS_beademingfront$withinborder_crv, na.rm = TRUE)

count_crv_beademingback <- sum(border_data_PS_beademingback$withinborder_crv, na.rm = TRUE)

count_hcHM_beademingfront <- sum(border_data_HM_beademingfront$withinborder_hcHM, na.rm = TRUE)

count_hcHM_beademingback <- sum(border_data_HM_beademingback$withinborder_hcHM, na.rm = TRUE)

# count van hierboven, maar dan gecorrigeerd voor nauwkeurigheid

border_data_PS_beademingfront_nauwkeurigheid <- Deviation_PS_beademingnaarvoren %>%

mutate(deviation_bl_gecorrigeerd = deviation_bl - -1.2300000) %>% # getal is mean_deviation_bl_front uit summary

mutate(deviation_hc_gecorrigeerd = deviation_hc - -0.004385965) %>% # getal is mean_deviation_hc_front uit summary

mutate(deviation_crv_gecorrigeerd = deviation_crv - -3.264306) # getal is mean_deviation_crv_front uit summary

border_data_PS_beademingfront_nauwkeurigheid <- border_data_PS_beademingfront_nauwkeurigheid %>%

mutate(withinborder_bl = deviation_bl_gecorrigeerd >= -bl_limit & deviation_bl_gecorrigeerd <= bl_limit, na.rm = TRUE) %>%

mutate(withinborder_hcPS = deviation_hc_gecorrigeerd >= -hc_limit & deviation_hc_gecorrigeerd <= hc_limit, na.rm = TRUE) %>%

mutate(withinborder_crv = deviation_crv_gecorrigeerd>= -crv_limit & deviation_crv_gecorrigeerd <= crv_limit, na.rm = TRUE)

border_data_PS_beademingback_nauwkeurigheid <- Deviation_PS_beademingnaarachter %>%

mutate(deviation_bl_gecorrigeerd = deviation_bl - -0.5258333) %>% # getal is mean_deviation_bl_back uit summary

mutate(deviation_hc_gecorrigeerd = deviation_hc - -0.033586957) %>% # getal is mean_deviation_hc_back uit summary

mutate(deviation_crv_gecorrigeerd = deviation_crv - -3.589873) # getal is mean_deviation_crv_back uit summary

border_data_PS_beademingback_nauwkeurigheid <- border_data_PS_beademingback_nauwkeurigheid %>%

mutate(withinborder_bl = deviation_bl_gecorrigeerd >= -bl_limit & deviation_bl_gecorrigeerd <= bl_limit, na.rm = TRUE) %>%

mutate(withinborder_hcPS = deviation_hc_gecorrigeerd >= -hc_limit & deviation_hc_gecorrigeerd <= hc_limit, na.rm = TRUE) %>%

mutate(withinborder_crv = deviation_crv_gecorrigeerd >= -crv_limit & deviation_crv_gecorrigeerd <= crv_limit, na.rm = TRUE)

border_data_HM_beademingfront_nauwkeurigheid <- Deviation_HM_beademingnaarvoren %>%

mutate(deviation_hc_gecorrigeerd = deviation_hc - -0.222982456) # getal is mean_deviation_hc_HM front uit summary

border_data_HM_beademingfront_nauwkeurigheid <- border_data_HM_beademingfront_nauwkeurigheid %>%

mutate(withinborder_hcHM = deviation_hc_gecorrigeerd >= -hc_limit & deviation_hc_gecorrigeerd <= hc_limit, na.rm = TRUE)

border_data_HM_beademingback_nauwkeurigheid <- Deviation_HM_beademingnaarachter %>%

mutate(deviation_hc_gecorrigeerd = deviation_hc - -0.333541667) # getal is mean_deviation_hc_HM back uit summary

border_data_HM_beademingback_nauwkeurigheid <- border_data_HM_beademingback_nauwkeurigheid %>%

mutate(withinborder_hcHM = deviation_hc_gecorrigeerd >= -hc_limit & deviation_hc_gecorrigeerd <= hc_limit, na.rm = TRUE)

count_bl_beademingfront_nauwkeurigheid <- sum(border_data_PS_beademingfront_nauwkeurigheid$withinborder_bl, na.rm = TRUE)

count_bl_beademingback_nauwkeurigheid <- sum(border_data_PS_beademingback_nauwkeurigheid$withinborder_bl, na.rm = TRUE)

count_hcPS_beademingfront_nauwkeurigheid <- sum(border_data_PS_beademingfront_nauwkeurigheid$withinborder_hcPS, na.rm = TRUE)

count_hcPS_beademingback_nauwkeurigheid <- sum(border_data_PS_beademingback_nauwkeurigheid$withinborder_hcPS, na.rm = TRUE)

count_crv_beademingfront_nauwkeurigheid <- sum(border_data_PS_beademingfront_nauwkeurigheid$withinborder_crv, na.rm = TRUE)

count_crv_beademingback_nauwkeurigheid <- sum(border_data_PS_beademingback_nauwkeurigheid$withinborder_crv, na.rm = TRUE)

count_hcHM_beademingfront_nauwkeurigheid <- sum(border_data_HM_beademingfront_nauwkeurigheid$withinborder_hcHM, na.rm = TRUE)

count_hcHM_beademingback_nauwkeurigheid <- sum(border_data_HM_beademingback_nauwkeurigheid$withinborder_hcHM, na.rm = TRUE)

# count van hierboven, maar dan gecorrigeerd voor nauwkeurigheid, in percentage en per pop opgeteld

border_data_PS_bl_Sophie <- Deviation_PS_Sophie %>%

mutate(withinborder_bl = deviation_bl_percentage >= medianbl$ymin & deviation_bl_percentage <= medianbl$ymax, na.rm = TRUE)

border_data_PS_bl_Ellen <- Deviation_PS_Ellen %>%

mutate(withinborder_bl = deviation_bl_percentage >= medianbl$ymin1 & deviation_bl_percentage <= medianbl$ymax1, na.rm = TRUE)

border_data_PS_bl_To <- Deviation_PS_To %>%

mutate(withinborder_bl = deviation_bl_percentage >= medianbl$ymin2 & deviation_bl_percentage <= medianbl$ymax2, na.rm = TRUE)

border_data_PS_beademingback_Sophie <- Deviation_PS_Sophie_beademingnaarachter %>%

mutate(withinborder_hc = deviation_hc_percentage >= medianhcPS_beademingnaarachter$ymin & deviation_hc_percentage <= medianhcPS_beademingnaarachter$ymax, na.rm = TRUE) %>%

mutate(withinborder_crv = deviation_crv_percentage >= mediancrvPS_beademingnaarachter$ymin & deviation_crv_percentage <= mediancrvPS_beademingnaarachter$ymax, na.rm = TRUE)

border_data_PS_beademingback_Ellen <- Deviation_PS_Ellen_beademingnaarachter %>%

mutate(withinborder_hc = deviation_hc_percentage >= medianhcPS_beademingnaarachter$ymin1 & deviation_hc_percentage <= medianhcPS_beademingnaarachter$ymax1, na.rm = TRUE) %>%

mutate(withinborder_crv = deviation_crv_percentage >= mediancrvPS_beademingnaarachter$ymin1 & deviation_crv_percentage <= mediancrvPS_beademingnaarachter$ymax1, na.rm = TRUE)

border_data_PS_beademingback_To <- Deviation_PS_To_beademingnaarachter %>%

mutate(withinborder_hc = deviation_hc_percentage >= medianhcPS_beademingnaarachter$ymin2 & deviation_hc_percentage <= medianhcPS_beademingnaarachter$ymax2, na.rm = TRUE) %>%

mutate(withinborder_crv = deviation_crv_percentage >= mediancrvPS_beademingnaarachter$ymin2 & deviation_crv_percentage <= mediancrvPS_beademingnaarachter$ymax2, na.rm = TRUE)

border_data_PS_beademingfront_Sophie <- Deviation_PS_Sophie_beademingnaarvoren %>%

mutate(withinborder_hc = deviation_hc_percentage >= medianhcPS_beademingnaarvoren$ymin & deviation_hc_percentage <= medianhcPS_beademingnaarvoren$ymax, na.rm = TRUE) %>%

mutate(withinborder_crv = deviation_crv_percentage >= mediancrvPS_beademingnaarvoren$ymin & deviation_crv_percentage <= mediancrvPS_beademingnaarvoren$ymax, na.rm = TRUE)

border_data_PS_beademingfront_Ellen <- Deviation_PS_Ellen_beademingnaarvoren %>%

mutate(withinborder_hc = deviation_hc_percentage >= medianhcPS_beademingnaarvoren$ymin1 & deviation_hc_percentage <= medianhcPS_beademingnaarvoren$ymax1, na.rm = TRUE) %>%

mutate(withinborder_crv = deviation_crv_percentage >= mediancrvPS_beademingnaarvoren$ymin1 & deviation_crv_percentage <= mediancrvPS_beademingnaarvoren$ymax1, na.rm = TRUE)

border_data_PS_beademingfront_To <- Deviation_PS_To_beademingnaarvoren %>%

mutate(withinborder_hc = deviation_hc_percentage >= medianhcPS_beademingnaarvoren$ymin2 & deviation_hc_percentage <= medianhcPS_beademingnaarvoren$ymax2, na.rm = TRUE) %>%

mutate(withinborder_crv = deviation_crv_percentage >= mediancrvPS_beademingnaarvoren$ymin2 & deviation_crv_percentage <= mediancrvPS_beademingnaarvoren$ymax2, na.rm = TRUE)

border_data_HM_beademingback_Sophie <- Deviation_HM_Sophie_beademingnaarachter %>%

mutate(withinborder_hc = deviation_hc_percentage >= medianhcHM_beademingnaarachter$ymin & deviation_hc_percentage <= medianhcHM_beademingnaarachter$ymax, na.rm = TRUE)

border_data_HM_beademingback_Ellen <- Deviation_HM_Ellen_beademingnaarachter %>%

mutate(withinborder_hc = deviation_hc_percentage >= medianhcHM_beademingnaarachter$ymin1 & deviation_hc_percentage <= medianhcHM_beademingnaarachter$ymax1, na.rm = TRUE)

border_data_HM_beademingback_To <- Deviation_HM_To_beademingnaarachter %>%

mutate(withinborder_hc = deviation_hc_percentage >= medianhcHM_beademingnaarachter$ymin2 & deviation_hc_percentage <= medianhcHM_beademingnaarachter$ymax2, na.rm = TRUE)

border_data_HM_beademingfront_Sophie <- Deviation_HM_Sophie_beademingnaarvoren %>%

mutate(withinborder_hc = deviation_hc_percentage >= medianhcHM_beademingnaarvoren$ymin & deviation_hc_percentage <= medianhcHM_beademingnaarvoren$ymax, na.rm = TRUE)

border_data_HM_beademingfront_Ellen <- Deviation_HM_Ellen_beademingnaarvoren %>%

mutate(withinborder_hc = deviation_hc_percentage >= medianhcHM_beademingnaarvoren$ymin1 & deviation_hc_percentage <= medianhcHM_beademingnaarvoren$ymax1, na.rm = TRUE)

border_data_HM_beademingfront_To <- Deviation_HM_To_beademingnaarvoren %>%

mutate(withinborder_hc = deviation_hc_percentage >= medianhcHM_beademingnaarvoren$ymin2 & deviation_hc_percentage <= medianhcHM_beademingnaarvoren$ymax2, na.rm = TRUE)

# optellen

echtecountAlles_bl <- sum(border_data_PS_bl_Sophie$withinborder_bl, border_data_PS_bl_Ellen$withinborder_bl, border_data_PS_bl_To$withinborder_bl, na.rm = TRUE)

echtecountAlles_hcPS_beademingfront <- sum(border_data_PS_beademingfront_Sophie$withinborder_hc, border_data_PS_beademingfront_Ellen$withinborder_hc, border_data_PS_beademingfront_To$withinborder_hc, na.rm = TRUE)

echtecountAlles_hcPS_beademingback <- sum(border_data_PS_beademingback_Sophie$withinborder_hc, border_data_PS_beademingback_Ellen$withinborder_hc, border_data_PS_beademingback_To$withinborder_hc, na.rm = TRUE)

echtecountAlles_crv_beademingfront <- sum(border_data_PS_beademingfront_Sophie$withinborder_crv, border_data_PS_beademingfront_Ellen$withinborder_crv, border_data_PS_beademingfront_To$withinborder_crv, na.rm = TRUE)

echtecountAlles_crv_beademingback <- sum(border_data_PS_beademingback_Sophie$withinborder_crv, border_data_PS_beademingback_Ellen$withinborder_crv, border_data_PS_beademingback_To$withinborder_crv, na.rm = TRUE)

echtecountAlles_hcHM_beademingfront <- sum(border_data_HM_beademingfront_Sophie$withinborder_hc, border_data_HM_beademingfront_Ellen$withinborder_hc, border_data_HM_beademingfront_To$withinborder_hc, na.rm = TRUE)

echtecountAlles_hcHM_beademingback <- sum(border_data_HM_beademingback_Sophie$withinborder_hc, border_data_HM_beademingback_Ellen$withinborder_hc, border_data_HM_beademingback_To$withinborder_hc, na.rm = TRUE)

# per doll

# Sophie, doll1

echtecountSophie_bl <- sum(border_data_PS_bl_Sophie$withinborder_bl, na.rm = TRUE)

echtecountSophie_hcPS_beademingfront <- sum(border_data_PS_beademingfront_Sophie$withinborder_hc, na.rm = TRUE)

echtecountSophie_hcPS_beademingback <- sum(border_data_PS_beademingback_Sophie$withinborder_hc, na.rm = TRUE)

echtecountSophie_crv_beademingfront <- sum(border_data_PS_beademingfront_Sophie$withinborder_crv, na.rm = TRUE)

echtecountSophie_crv_beademingback <- sum(border_data_PS_beademingback_Sophie$withinborder_crv, na.rm = TRUE)

echtecountSophie_hcHM_beademingfront <- sum(border_data_HM_beademingfront_Sophie$withinborder_hc, na.rm = TRUE)

echtecountSophie_hcHM_beademingback <- sum(border_data_HM_beademingback_Sophie$withinborder_hc, na.rm = TRUE)

"BL within limits doll 1:"

echtecountSophie_bl

"HC PS front within limits doll 1:"

echtecountSophie_hcPS_beademingfront

"HC PS back within limits doll 1:"

echtecountSophie_hcPS_beademingback

"CrV front within limits doll 1:"

echtecountSophie_crv_beademingfront

"CrV back within limits doll 1:"

echtecountSophie_crv_beademingback

"HC tape front within limits doll 1:"

echtecountSophie_hcHM_beademingfront

"HC tape back within limits doll 1:"

echtecountSophie_hcHM_beademingback

# Ellen, doll2

echtecountEllen_bl <- sum(border_data_PS_bl_Ellen$withinborder_bl, na.rm = TRUE)

echtecountEllen_hcPS_beademingfront <- sum(border_data_PS_beademingfront_Ellen$withinborder_hc, na.rm = TRUE)

echtecountEllen_hcPS_beademingback <- sum(border_data_PS_beademingback_Ellen$withinborder_hc, na.rm = TRUE)

echtecountEllen_crv_beademingfront <- sum(border_data_PS_beademingfront_Ellen$withinborder_crv, na.rm = TRUE)

echtecountEllen_crv_beademingback <- sum(border_data_PS_beademingback_Ellen$withinborder_crv, na.rm = TRUE)

echtecountEllen_hcHM_beademingfront <- sum(border_data_HM_beademingfront_Ellen$withinborder_hc, na.rm = TRUE)

echtecountEllen_hcHM_beademingback <- sum(border_data_HM_beademingback_Ellen$withinborder_hc, na.rm = TRUE)

"BL within limits doll 2:"

echtecountEllen_bl

"HC PS front within limits doll 2:"

echtecountEllen_hcPS_beademingfront

"HC PS back within limits doll 2:"

echtecountEllen_hcPS_beademingback

"CrV front within limits doll 2:"

echtecountEllen_crv_beademingfront

"CrV back within limits doll 2:"

echtecountEllen_crv_beademingback

"HC tape front within limits doll 2:"

echtecountEllen_hcHM_beademingfront

"HC tape back within limits doll 2:"

echtecountEllen_hcHM_beademingback

# To, doll3

echtecountTo_bl <- sum(border_data_PS_bl_To$withinborder_bl, na.rm = TRUE)

echtecountTo_hcPS_beademingfront <- sum(border_data_PS_beademingfront_To$withinborder_hc, na.rm = TRUE)

echtecountTo_hcPS_beademingback <- sum(border_data_PS_beademingback_To$withinborder_hc, na.rm = TRUE)

echtecountTo_crv_beademingfront <- sum(border_data_PS_beademingfront_To$withinborder_crv, na.rm = TRUE)

echtecountTo_crv_beademingback <- sum(border_data_PS_beademingback_To$withinborder_crv, na.rm = TRUE)

echtecountTo_hcHM_beademingfront <- sum(border_data_HM_beademingfront_To$withinborder_hc, na.rm = TRUE)

echtecountTo_hcHM_beademingback <- sum(border_data_HM_beademingback_To$withinborder_hc, na.rm = TRUE)

"BL within limits doll 3:"

echtecountTo_bl

"HC PS front within limits doll 3:"

echtecountTo_hcPS_beademingfront

"HC PS back within limits doll 3:"

echtecountTo_hcPS_beademingback

"CrV PS front within limits doll 3:"

echtecountTo_crv_beademingfront

"CrV PS back within limits doll 3:"

echtecountTo_crv_beademingback

"HC tape front within limits doll 3:"

echtecountTo_hcHM_beademingfront

"HC tape back within limits doll 3:"

echtecountTo_hcHM_beademingback

# ICC ------------

# inter --> eerste meting en wel of niet afwijking gebruiken? Afwijking gebruiken kan denk ik niet

# intra --> niet de afwijking gebruiken

# Intra ---------------

m1_PS_alleen_beademingfront <- Deviation %>%

filter (measurement == "m1" & device == "PS" & optiflow_clear == "front")

m1_PS_alleen_beademingback <- Deviation %>%

filter (measurement == "m1" & device == "PS" & optiflow_clear == "back")

m1_HM_alleen_beademingfront <- Deviation %>%

filter (measurement == "m1" & device == "HM" & optiflow_clear == "front")

m1_HM_alleen_beademingback <- Deviation %>%

filter (measurement == "m1" & device == "HM" & optiflow_clear == "back")

m2_PS_alleen_beademingfront <- Deviation %>%

filter (measurement == "m2" & device == "PS" & optiflow_clear == "front")

m2_PS_alleen_beademingback <- Deviation %>%

filter (measurement == "m2" & device == "PS" & optiflow_clear == "back")

m2_HM_alleen_beademingfront <- Deviation %>%

filter (measurement == "m2" & device == "HM" & optiflow_clear == "front")

m2_HM_alleen_beademingback <- Deviation %>%

filter (measurement == "m2" & device == "HM" & optiflow_clear == "back")

m3_PS_alleen_beademingfront <- Deviation %>%

filter (measurement == "m3" & device == "PS" & optiflow_clear == "front")

m3_PS_alleen_beademingback <- Deviation %>%

filter (measurement == "m3" & device == "PS" & optiflow_clear == "back")

m3_HM_alleen_beademingfront <- Deviation %>%

filter (measurement == "m3" & device == "HM" & optiflow_clear == "front")

m3_HM_alleen_beademingback <- Deviation %>%

filter (measurement == "m3" & device == "HM" & optiflow_clear == "back")

m1_PS_alleen <- Deviation %>%

filter (measurement == "m1" & device == "PS")

m2_PS_alleen <- Deviation %>%

filter (measurement == "m2" & device == "PS")

m3_PS_alleen <- Deviation %>%

filter (measurement == "m3" & device == "PS")

# bl intra optiflow back icc --------------

selectbl_intra_1 <- m1_PS_alleen %>%

select(deviation_bl_percentage)

selectbl_intra_1 <- t(selectbl_intra_1)

selectbl_intra_1 <- as.data.frame(selectbl_intra_1)

selectbl_intra_2 <- m2_PS_alleen %>%

select(deviation_bl_percentage)

selectbl_intra_2 <- t(selectbl_intra_2)

selectbl_intra_2 <- as.data.frame(selectbl_intra_2)

selectbl_intra_3 <- m3_PS_alleen %>%

select(deviation_bl_percentage)

selectbl_intra_3 <- t(selectbl_intra_3)

selectbl_intra_3 <- as.data.frame(selectbl_intra_3)

align_raters <- function(df, max_cols) {

if (ncol(df) < max_cols) {

missing_cols <- max_cols - ncol(df)

df[, (ncol(df) + 1):(ncol(df) + missing_cols)] <- NA

colnames(df)[(ncol(df) - missing_cols + 1):ncol(df)] <- paste0("rater", (ncol(df) - missing_cols + 1):ncol(df))

}

return(df)

}

max_raters_bl <- max(ncol(selectbl_intra_1), ncol(selectbl_intra_2), ncol(selectbl_intra_3))

selectbl_intra_1 <- align_raters(selectbl_intra_1, max_raters_bl)

selectbl_intra_2 <- align_raters(selectbl_intra_2, max_raters_bl)

selectbl_intra_3 <- align_raters(selectbl_intra_3, max_raters_bl)

colnames(selectbl_intra_1) <- colnames(selectbl_intra_2) <- colnames(selectbl_intra_3) <- paste0("rater", 1:max_raters_bl)

combined_data_bl_intra <- rbind(selectbl_intra_1, selectbl_intra_2, selectbl_intra_3)

combined_data_bl_intra_t <- t(combined_data_bl_intra)

combined_data_bl_intra_t <- as.data.frame(combined_data_bl_intra_t)

icc_intra_bl <- icc(combined_data_bl_intra_t, model = "twoway", type = "agreement", unit = "single")

# crv intra optiflow front icc --------------

selectcrvfr_intra_1 <- m1_PS_alleen_beademingfront %>%

select(deviation_crv_percentage)

selectcrvfr_intra_1 <- na.omit(selectcrvfr_intra_1)

selectcrvfr_intra_1 <- t(selectcrvfr_intra_1)

selectcrvfr_intra_1 <- as.data.frame(selectcrvfr_intra_1)

selectcrvfr_intra_2 <- m2_PS_alleen_beademingfront %>%

select(deviation_crv_percentage)

selectcrvfr_intra_2 <- na.omit(selectcrvfr_intra_2)

selectcrvfr_intra_2 <- t(selectcrvfr_intra_2)

selectcrvfr_intra_2 <- as.data.frame(selectcrvfr_intra_2)

selectcrvfr_intra_3 <- m3_PS_alleen_beademingfront %>%

select(deviation_crv_percentage)

selectcrvfr_intra_3 <- na.omit(selectcrvfr_intra_3)

selectcrvfr_intra_3 <- t(selectcrvfr_intra_3)

selectcrvfr_intra_3 <- as.data.frame(selectcrvfr_intra_3)

align_raters <- function(df, max_cols) {

if (ncol(df) < max_cols) {

missing_cols <- max_cols - ncol(df)

df[, (ncol(df) + 1):(ncol(df) + missing_cols)] <- NA

colnames(df)[(ncol(df) - missing_cols + 1):ncol(df)] <- paste0("rater", (ncol(df) - missing_cols + 1):ncol(df))

}

return(df)

}

max_raters_crvfr <- max(ncol(selectcrvfr_intra_1), ncol(selectcrvfr_intra_2), ncol(selectcrvfr_intra_3))

selectcrvfr_intra_1 <- align_raters(selectcrvfr_intra_1, max_raters_crvfr)

selectcrvfr_intra_2 <- align_raters(selectcrvfr_intra_2, max_raters_crvfr)

selectcrvfr_intra_3 <- align_raters(selectcrvfr_intra_3, max_raters_crvfr)

colnames(selectcrvfr_intra_1) <- colnames(selectcrvfr_intra_2) <- colnames(selectcrvfr_intra_3) <- paste0("rater", 1:max_raters_crvfr)

combined_data_crvfr_intra <- rbind(selectcrvfr_intra_1, selectcrvfr_intra_2, selectcrvfr_intra_3)

combined_data_crvfr_intra_t <- t(combined_data_crvfr_intra)

combined_data_crvfr_intra_t <- as.data.frame(combined_data_crvfr_intra_t)

icc_intra_crv_beademingfront <- icc(combined_data_crvfr_intra_t, model = "twoway", type = "agreement", unit = "single")

# crv intra optiflow back icc --------------

selectcrvba_intra_1 <- m1_PS_alleen_beademingback %>%

select(deviation_crv_percentage)

selectcrvba_intra_1 <- na.omit(selectcrvba_intra_1)

selectcrvba_intra_1 <- t(selectcrvba_intra_1)

selectcrvba_intra_1 <- as.data.frame(selectcrvba_intra_1)

selectcrvba_intra_2 <- m2_PS_alleen_beademingback %>%

select(deviation_crv_percentage)

selectcrvba_intra_2 <- na.omit(selectcrvba_intra_2)

selectcrvba_intra_2 <- t(selectcrvba_intra_2)

selectcrvba_intra_2 <- as.data.frame(selectcrvba_intra_2)

selectcrvba_intra_3 <- m3_PS_alleen_beademingback %>%

select(deviation_crv_percentage)

selectcrvba_intra_3 <- na.omit(selectcrvba_intra_3)

selectcrvba_intra_3 <- t(selectcrvba_intra_3)

selectcrvba_intra_3 <- as.data.frame(selectcrvba_intra_3)

selectcrvba_intra_3 <- selectcrvba_intra_3[, -15]

align_raters <- function(df, max_cols) {

if (ncol(df) < max_cols) {

missing_cols <- max_cols - ncol(df)

df[, (ncol(df) + 1):(ncol(df) + missing_cols)] <- NA

colnames(df)[(ncol(df) - missing_cols + 1):ncol(df)] <- paste0("rater", (ncol(df) - missing_cols + 1):ncol(df))

}

return(df)

}

max_raters_crvba <- max(ncol(selectcrvba_intra_1), ncol(selectcrvba_intra_2), ncol(selectcrvba_intra_3))

selectcrvba_intra_1 <- align_raters(selectcrvba_intra_1, max_raters_crvba)

selectcrvba_intra_2 <- align_raters(selectcrvba_intra_2, max_raters_crvba)

selectcrvba_intra_3 <- align_raters(selectcrvba_intra_3, max_raters_crvba)

colnames(selectcrvba_intra_1) <- colnames(selectcrvba_intra_2) <- colnames(selectcrvba_intra_3) <- paste0("rater", 1:max_raters_crvba)

combined_data_crvba_intra <- rbind(selectcrvba_intra_1, selectcrvba_intra_2, selectcrvba_intra_3)

combined_data_crvba_intra_t <- t(combined_data_crvba_intra)

combined_data_crvba_intra_t <- as.data.frame(combined_data_crvba_intra_t)

icc_intra_crv_beademingback <- icc(combined_data_crvba_intra_t, model = "twoway", type = "agreement", unit = "single")

# HC PrSc intra optiflow front icc --------------

selecthcPSfr_intra_1 <- m1_PS_alleen_beademingfront %>%

select(deviation_hc_percentage)

selecthcPSfr_intra_1 <- na.omit(selecthcPSfr_intra_1)

selecthcPSfr_intra_1 <- t(selecthcPSfr_intra_1)

selecthcPSfr_intra_1 <- as.data.frame(selecthcPSfr_intra_1)

selecthcPSfr_intra_2 <- m2_PS_alleen_beademingfront %>%

select(deviation_hc_percentage)

selecthcPSfr_intra_2 <- na.omit(selecthcPSfr_intra_2)

selecthcPSfr_intra_2 <- t(selecthcPSfr_intra_2)

selecthcPSfr_intra_2 <- as.data.frame(selecthcPSfr_intra_2)

selecthcPSfr_intra_3 <- m3_PS_alleen_beademingfront %>%

select(deviation_hc_percentage)

selecthcPSfr_intra_3 <- na.omit(selecthcPSfr_intra_3)

selecthcPSfr_intra_3 <- t(selecthcPSfr_intra_3)

selecthcPSfr_intra_3 <- as.data.frame(selecthcPSfr_intra_3)

align_raters <- function(df, max_cols) {

if (ncol(df) < max_cols) {

missing_cols <- max_cols - ncol(df)

df[, (ncol(df) + 1):(ncol(df) + missing_cols)] <- NA

colnames(df)[(ncol(df) - missing_cols + 1):ncol(df)] <- paste0("rater", (ncol(df) - missing_cols + 1):ncol(df))

}

return(df)

}

max_raters_hcPSfr <- max(ncol(selecthcPSfr_intra_1), ncol(selecthcPSfr_intra_2), ncol(selecthcPSfr_intra_3))

selecthcPSfr_intra_1 <- align_raters(selecthcPSfr_intra_1, max_raters_hcPSfr)

selecthcPSfr_intra_2 <- align_raters(selecthcPSfr_intra_2, max_raters_hcPSfr)

selecthcPSfr_intra_3 <- align_raters(selecthcPSfr_intra_3, max_raters_hcPSfr)

colnames(selecthcPSfr_intra_1) <- colnames(selecthcPSfr_intra_2) <- colnames(selecthcPSfr_intra_3) <- paste0("rater", 1:max_raters_hcPSfr)

combined_data_hcPSfr_intra <- rbind(selecthcPSfr_intra_1, selecthcPSfr_intra_2, selecthcPSfr_intra_3)

combined_data_hcPSfr_intra_t <- t(combined_data_hcPSfr_intra)

combined_data_hcPSfr_intra_t <- as.data.frame(combined_data_hcPSfr_intra_t)

icc_intra_hcPS_beademingfront <- icc(combined_data_hcPSfr_intra_t, model = "twoway", type = "agreement", unit = "single")

# HC PrSc intra optiflow back icc --------------

selecthcPSba_intra_1 <- m1_PS_alleen_beademingback %>%

select(deviation_hc_percentage)

selecthcPSba_intra_1 <- na.omit(selecthcPSba_intra_1)

selecthcPSba_intra_1 <- t(selecthcPSba_intra_1)

selecthcPSba_intra_1 <- as.data.frame(selecthcPSba_intra_1)

selecthcPSba_intra_2 <- m2_PS_alleen_beademingback %>%

select(deviation_hc_percentage)

selecthcPSba_intra_2 <- na.omit(selecthcPSba_intra_2)

selecthcPSba_intra_2 <- t(selecthcPSba_intra_2)

selecthcPSba_intra_2 <- as.data.frame(selecthcPSba_intra_2)

selecthcPSba_intra_3 <- m3_PS_alleen_beademingback %>%

select(deviation_hc_percentage)

selecthcPSba_intra_3 <- na.omit(selecthcPSba_intra_3)

selecthcPSba_intra_3 <- t(selecthcPSba_intra_3)

selecthcPSba_intra_3 <- as.data.frame(selecthcPSba_intra_3)

selecthcPSba_intra_3 <- selecthcPSba_intra_3[, -15]

align_raters <- function(df, max_cols) {

if (ncol(df) < max_cols) {

missing_cols <- max_cols - ncol(df)

df[, (ncol(df) + 1):(ncol(df) + missing_cols)] <- NA

colnames(df)[(ncol(df) - missing_cols + 1):ncol(df)] <- paste0("rater", (ncol(df) - missing_cols + 1):ncol(df))

}

return(df)

}

max_raters_hcPSba <- max(ncol(selecthcPSba_intra_1), ncol(selecthcPSba_intra_2), ncol(selecthcPSba_intra_3))

selecthcPSba_intra_1 <- align_raters(selecthcPSba_intra_1, max_raters_hcPSba)

selecthcPSba_intra_2 <- align_raters(selecthcPSba_intra_2, max_raters_hcPSba)

selecthcPSba_intra_3 <- align_raters(selecthcPSba_intra_3, max_raters_hcPSba)

colnames(selecthcPSba_intra_1) <- colnames(selecthcPSba_intra_2) <- colnames(selecthcPSba_intra_3) <- paste0("rater", 1:max_raters_hcPSba)

combined_data_hcPSba_intra <- rbind(selecthcPSba_intra_1, selecthcPSba_intra_2, selecthcPSba_intra_3)

combined_data_hcPSba_intra_t <- t(combined_data_hcPSba_intra)

combined_data_hcPSba_intra_t <- as.data.frame(combined_data_hcPSba_intra_t)

icc_intra_hcPS_beademingback <- icc(combined_data_hcPSba_intra_t, model = "twoway", type = "agreement", unit = "single")

# hcHM intra beademing front icc --------------

selecthcHMfr_intra_1 <- m1_HM_alleen_beademingfront %>%

select(deviation_hc_percentage)

selecthcHMfr_intra_1 <- na.omit(selecthcHMfr_intra_1)

selecthcHMfr_intra_1 <- t(selecthcHMfr_intra_1)

selecthcHMfr_intra_1 <- as.data.frame(selecthcHMfr_intra_1)

selecthcHMfr_intra_2 <- m2_HM_alleen_beademingfront %>%

select(deviation_hc_percentage)

selecthcHMfr_intra_2 <- na.omit(selecthcHMfr_intra_2)

selecthcHMfr_intra_2 <- t(selecthcHMfr_intra_2)

selecthcHMfr_intra_2 <- as.data.frame(selecthcHMfr_intra_2)

selecthcHMfr_intra_3 <- m3_HM_alleen_beademingfront %>%

select(deviation_hc_percentage)

selecthcHMfr_intra_3 <- na.omit(selecthcHMfr_intra_3)

selecthcHMfr_intra_3 <- t(selecthcHMfr_intra_3)

selecthcHMfr_intra_3 <- as.data.frame(selecthcHMfr_intra_3)

align_raters <- function(df, max_cols) {

if (ncol(df) < max_cols) {

missing_cols <- max_cols - ncol(df)

df[, (ncol(df) + 1):(ncol(df) + missing_cols)] <- NA

colnames(df)[(ncol(df) - missing_cols + 1):ncol(df)] <- paste0("rater", (ncol(df) - missing_cols + 1):ncol(df))

}

return(df)

}

max_raters_hcHMfr <- max(ncol(selecthcHMfr_intra_1), ncol(selecthcHMfr_intra_2), ncol(selecthcHMfr_intra_3))

selecthcHMfr_intra_1 <- align_raters(selecthcHMfr_intra_1, max_raters_hcHMfr)

selecthcHMfr_intra_2 <- align_raters(selecthcHMfr_intra_2, max_raters_hcHMfr)

selecthcHMfr_intra_3 <- align_raters(selecthcHMfr_intra_3, max_raters_hcHMfr)

colnames(selecthcHMfr_intra_1) <- colnames(selecthcHMfr_intra_2) <- colnames(selecthcHMfr_intra_3) <- paste0("rater", 1:max_raters_hcHMfr)

combined_data_hcHMfr_intra <- rbind(selecthcHMfr_intra_1, selecthcHMfr_intra_2, selecthcHMfr_intra_3)

combined_data_hcHMfr_intra_t <- t(combined_data_hcHMfr_intra)

combined_data_hcHMfr_intra_t <- as.data.frame(combined_data_hcHMfr_intra_t)

icc_intra_hcHM_beademingfront <- icc(combined_data_hcHMfr_intra_t, model = "twoway", type = "agreement", unit = "single")

# hcHM intra beademing back icc --------------

selecthcHMba_intra_1 <- m1_HM_alleen_beademingback %>%

select(deviation_hc_percentage)

selecthcHMba_intra_1 <- na.omit(selecthcHMba_intra_1)

selecthcHMba_intra_1 <- t(selecthcHMba_intra_1)

selecthcHMba_intra_1 <- as.data.frame(selecthcHMba_intra_1)

selecthcHMba_intra_2 <- m2_HM_alleen_beademingback %>%

select(deviation_hc_percentage)

selecthcHMba_intra_2 <- na.omit(selecthcHMba_intra_2)

selecthcHMba_intra_2 <- t(selecthcHMba_intra_2)

selecthcHMba_intra_2 <- as.data.frame(selecthcHMba_intra_2)

selecthcHMba_intra_3 <- m3_HM_alleen_beademingback %>%

select(deviation_hc_percentage)

selecthcHMba_intra_3 <- na.omit(selecthcHMba_intra_3)

selecthcHMba_intra_3 <- t(selecthcHMba_intra_3)

selecthcHMba_intra_3 <- as.data.frame(selecthcHMba_intra_3)

align_raters <- function(df, max_cols) {

if (ncol(df) < max_cols) {

missing_cols <- max_cols - ncol(df)

df[, (ncol(df) + 1):(ncol(df) + missing_cols)] <- NA

colnames(df)[(ncol(df) - missing_cols + 1):ncol(df)] <- paste0("rater", (ncol(df) - missing_cols + 1):ncol(df))

}

return(df)

}

max_raters_hcHMba <- max(ncol(selecthcHMba_intra_1), ncol(selecthcHMba_intra_2), ncol(selecthcHMba_intra_3))

selecthcHMba_intra_1 <- align_raters(selecthcHMba_intra_1, max_raters_hcHMba)

selecthcHMba_intra_2 <- align_raters(selecthcHMba_intra_2, max_raters_hcHMba)

selecthcHMba_intra_3 <- align_raters(selecthcHMba_intra_3, max_raters_hcHMba)

colnames(selecthcHMba_intra_1) <- colnames(selecthcHMba_intra_2) <- colnames(selecthcHMba_intra_3) <- paste0("rater", 1:max_raters_hcHMba)

combined_data_hcHMba_intra <- rbind(selecthcHMba_intra_1, selecthcHMba_intra_2, selecthcHMba_intra_3)

combined_data_hcHMba_intra_t <- t(combined_data_hcHMba_intra)

combined_data_hcHMba_intra_t <- as.data.frame(combined_data_hcHMba_intra_t)

icc_intra_hcHM_beademingback <- icc(combined_data_hcHMba_intra_t, model = "twoway", type = "agreement", unit = "single")

# Intra totaal ------------

Intraobserver <- list(

icc_intra_bl = icc(combined_data_bl_intra_t, model = "twoway", type = "agreement", unit = "single"),

icc_intra_crv_beademingfront = icc(combined_data_crvfr_intra_t, model = "twoway", type = "agreement", unit = "single"),

icc_intra_crv_beademingback = icc(combined_data_crvba_intra_t, model = "twoway", type = "agreement", unit = "single"),

icc_intra_hcPS_beademingfront = icc(combined_data_hcPSfr_intra_t, model = "twoway", type = "agreement", unit = "single"),

icc_intra_hcPS_beademingback = icc(combined_data_hcPSba_intra_t, model = "twoway", type = "agreement", unit = "single"),

icc_intra_hcHM_beademingfront = icc(combined_data_hcHMfr_intra_t, model = "twoway", type = "agreement", unit = "single"),

icc_intra_hcHM_beademingback = icc(combined_data_hcHMba_intra_t, model = "twoway", type = "agreement", unit = "single"))

# Inter -------------

Interobserver <- list(

icc_inter_bl = icc(combined_data_bl_intra, model = "twoway", type = "agreement", unit = "single"),

icc_inter_crv_beademingfront = icc(combined_data_crvfr_intra, model = "twoway", type = "agreement", unit = "single"),

icc_inter_crv_beademingback = icc(combined_data_crvba_intra, model = "twoway", type = "agreement", unit = "single"),

icc_inter_hcPS_beademingfront = icc(combined_data_hcPSfr_intra, model = "twoway", type = "agreement", unit = "single"),

icc_inter_hcPS_beademingback = icc(combined_data_hcPSba_intra, model = "twoway", type = "agreement", unit = "single"),

icc_inter_hcHM_beademingfront = icc(combined_data_hcHMfr_intra, model = "twoway", type = "agreement", unit = "single"),

icc_inter_hcHM_beademingback = icc(combined_data_hcHMba_intra, model = "twoway", type = "agreement", unit = "single"))

# #R-Script Tape versus Preemie Scanner direct comparison: BlandAltman, Pearson Correlation, ICC, standard error of measurement SEM, smallest detectable change SDC

# Load necessary libraries

library(dplyr)

library(ggplot2)

library(readxl)

library(tidyr)

install.packages("irr")

library(irr)

# Load the data

data <- read_excel("Data_PreemieScanner.xlsx")

# Clean and prepare the data

data <- data %>%

rename(

measurement = `Measurement m#`,

hc = HC,

device = Device,

id = ID

) %>%

filter(!is.na(hc)) %>%

group_by(id, device) %>%

summarise(mean_hc = mean(hc, na.rm = TRUE), .groups = "drop") %>%

pivot_wider(names_from = device, values_from = mean_hc) %>%

filter(!is.na(HM) & !is.na(PS)) %>%

mutate(

mean_hc = (HM + PS) / 2,

diff_hc = HM - PS

)

data

# Prepare data for ICC: two columns, one for each device

icc_data <- data %>%

select(HM, PS)

# Calculate ICC (two-way mixed, absolute agreement)

icc_result <- icc(icc_data, model = "twoway", type = "agreement", unit = "single")

# Print ICC value

print(icc_result)

# Calculate mean and limits of agreement

mean_diff <- mean(data$diff_hc)

sd_diff <- sd(data$diff_hc)

loa_upper <- mean_diff + 1.96 * sd_diff

loa_lower <- mean_diff - 1.96 * sd_diff# Calculate correlation coefficient

correlation <- cor(data$HM, data$PS)

print(paste("Pearson Correlation:", round(correlation, 3)))

# Calculate ICC (two-way mixed, absolute agreement)

icc_result <- icc(icc_data, model = "twoway", type = "agreement", unit = "single")

icc_value <- icc_result$value

print(paste("ICC:", round(icc_value, 3)))

# Calculate SEM using ICC

sem <- sd_diff * sqrt(1 - icc_value)

print(paste("Standard Error of Measurement (SEM) using ICC:", round(sem, 3)))

# Create Bland-Altman plot with ICC and SEM

ggplot(data, aes(x = mean_hc, y = diff_hc)) +

geom_point(alpha = 0.7) +

geom_hline(yintercept = mean_diff, color = "red", linetype = "dashed", linewidth = 1) +

geom_hline(yintercept = loa_upper, color = "gray40", linetype = "dashed", linewidth = 1) +

geom_hline(yintercept = loa_lower, color = "gray40", linetype = "dashed", linewidth = 1) +

annotate("text", x = max(data$mean_hc), y = mean_diff - 0.05,

label = paste("Mean Diff =", round(mean_diff, 2)),

hjust = 1.1, color = "red") +

annotate("text", x = max(data$mean_hc), y = loa_upper - 0.05,

label = paste("+1.96 SD =", round(loa_upper, 2)),

hjust = 1.1, color = "gray40") +

annotate("text", x = max(data$mean_hc), y = loa_lower - 0.05,

label = paste("-1.96 SD =", round(loa_lower, 2)),

hjust = 1.1, color = "gray40") +

annotate("text", x = max(data$mean_hc), y = loa_lower + 0.2,

label = paste("ICC =", round(icc_value, 3)),

hjust = 1.2, color = "blue") +

annotate("text", x = max(data$mean_hc), y = loa_lower + 0.15,

label = paste("SEM =", round(sem, 3)),

hjust = 1.2, color = "blue") +

annotate("text", x = max(data$mean_hc), y = loa_lower + 0.25,

label = paste("Pearson Correlation =", round(correlation, 3)),

hjust = 1.1, color = "blue") +

labs(

title = "Bland-Altman Plot: HC Measuring Tape (MT) vs HC PreemieScanner(PS)",

x = "Mean HC (MT & PS) [cm]",

y = "Difference in HC (MT - PS) [cm]"

) +

theme_minimal()

# #R-Script Calculation of Standard Error of Measurement SEM and Smallest Detectable Change SDC

**# Load necessary libraries**

**library(dplyr)**

**library(readxl)**

**# Load the data**

**device_data <- read_excel("Data_PreemieScanner.xlsx") %>%**

**rename(**

**measurement = `Measurement m#`,**

**hc = HC,**

**bl = BL,**

**crv = CrV,**

**device = Device,**

**id = ID,**

**doll = Doll,**

**optiflow = "Optiflow attached to front/back of the head"**

**)**

**ground_truth <- read_excel("GroundTruth.xlsx") %>%**

**rename(hc_truth = hc, bl_truth = bl, crv_truth = crv)**

**# Merge device data with ground truth by 'doll'**

**merged_data <- device_data %>%**

**inner_join(ground_truth, by = "doll") %>%**

**mutate(**

**error_hc = hc - hc_truth,**

**error_crv = crv - crv_truth,**

**error_bl = bl - bl_truth**

**)**

**merged_data # show merged_data**

**# Calculate SEM and SDC for each device**

**sem_sdc_results <- merged_data %>%**

**group_by(device) %>%**

**summarise(**

**SEM_hc = sd(error_hc, na.rm = TRUE),**

**SDC_hc = 1.96 * sqrt(2) * SEM_hc,**

**SEM_bl = sd(error_bl, na.rm = TRUE),**

**SDC_bl = 1.96 * sqrt(2) * SEM_bl,**

**SEM_crv = sd(error_crv, na.rm = TRUE),**

**SDC_crv = 1.96 * sqrt(2) * SEM_crv,**

**.groups = "drop"**

**)**

**# View results**

**sem_sdc_results**

**print(sem_sdc_results)**

**# Calculate SEM and SDC grouped by device, doll, and optiflow**

**sem_sdc_by_group <- merged_data %>%**

**group_by(device, doll, optiflow) %>%**

**summarise(**

**SEM_hc = sd(error_hc, na.rm = TRUE),**

**SDC_hc = 1.96 * sqrt(2) * SEM_hc,**

**SEM_crv = sd(error_crv, na.rm = TRUE),**

**SDC_crv = 1.96 * sqrt(2) * SEM_crv,**

**SEM_bl = sd(error_bl, na.rm = TRUE),**

**SDC_bl = 1.96 * sqrt(2) * SEM_bl,**

**.groups = "drop"**

**)**

**# View results**

**print(sem_sdc_by_group)**

**sem_sdc_by_group**

**# Calculate SEM and SDC for BL, only PS, optiflow front and back combined, grouped by doll and device**

**sem_sdc_bl_by_doll_device <- merged_data %>%**

**filter(device=="PS")%>%**

**group_by(doll, device) %>%**

**summarise(**

**SEM_hc = sd(error_hc, na.rm = TRUE),**

**SDC_hc = 1.96 * sqrt(2) * SEM_hc,**

**SEM_crv = sd(error_crv, na.rm = TRUE),**

**SDC_crv = 1.96 * sqrt(2) * SEM_crv,**

**SEM_bl = sd(error_bl, na.rm = TRUE),**

**SDC_bl = 1.96 * sqrt(2) * SEM_bl,**

**.groups = "drop"**

**)**

**# View results**

**print(sem_sdc_bl_by_doll_device)**

**sem_sdc_bl_by_doll_device**

**# Calculate SEM and SDC groupedby optiflow, device, all dolls combined**

**sem_sdc_by_device_optiflow <- merged_data %>%**

**group_by(device, optiflow) %>%**

**summarise(**

**SEM_hc = sd(error_hc, na.rm = TRUE),**

**SDC_hc = 1.96 * sqrt(2) * SEM_hc,**

**SEM_crv = sd(error_crv, na.rm = TRUE),**

**SDC_crv = 1.96 * sqrt(2) * SEM_crv,**

**SEM_bl = sd(error_bl, na.rm = TRUE),**

**SDC_bl = 1.96 * sqrt(2) * SEM_bl,**

**.groups = "drop"**

**)**

**# View results**

**print(sem_sdc_by_device_optiflow)**

**sem_sdc_by_device_optiflow**
